# Transcriptional response of *Fusarium oxysporum* and *Neocosmospora solani* challenged with amphotericin B or posaconazole

**DOI:** 10.1099/mic.0.000927

**Published:** 2020-07-09

**Authors:** A. Castillo-Castañeda, S. J. Cañas-Duarte, M. Guevara-Suarez, J. Guarro, S. Restrepo, A. M. Celis Ramírez

**Affiliations:** ^1^​ Grupo de Investigación Celular y Molecular de Microorganismos Patógenos (CeMoP), Departamento de Ciencias Biológicas, Universidad de Los Andes, Bogotá, Colombia; ^2^​ Laboratorio de Micología y Fitopatología (LAMFU), Facultad de Ingeniería, Universidad de Los Andes, Bogotá, Colombia; ^3^​ Department of Systems Biology, Blavatnik Institute at Harvard Medical School, Harvard University, Boston, MA, USA; ^4^​ Facultat de Medicina I Ciéncies de la Salut, Departament de Ciéncies Médiques Básiques, Unitat de Microbiología. Universitat de Rovira I Virgili, Reus, España

**Keywords:** fusaria, amphotericin B, posaconazole, transcriptional changes, RNA-Seq

## Abstract

Some species of fusaria are well-known pathogens of humans, animals and plants. *Fusarium oxysporum* and *Neocosmospora solani* (formerly *Fusarium solani*) cause human infections that range from onychomycosis or keratitis to severe disseminated infections. In general, these infections are difficult to treat due to poor therapeutic responses in immunocompromised patients. Despite that, little is known about the molecular mechanisms and transcriptional changes responsible for the antifungal resistance in fusaria. To shed light on the transcriptional response to antifungals, we carried out the first reported high-throughput RNA-seq analysis for *F. oxysporum* and *N. solani* that had been exposed to amphotericin B (AMB) and posaconazole (PSC). We detected significant differences between the transcriptional profiles of the two species and we found that some oxidation-reduction, metabolic, cellular and transport processes were regulated differentially by both fungi. The same was found with several genes from the ergosterol synthesis, efflux pumps, oxidative stress response and membrane biosynthesis pathways. A significant up-regulation of the C-22 sterol desaturase (*ERG5*), the sterol 24-C-methyltransferase (*ERG6*) gene, the glutathione S-transferase (*GST*) gene and of several members of the major facilitator superfamily (*MSF*) was demonstrated in this study after treating *F. oxysporum* with AMB. These results offer a good overview of transcriptional changes after exposure to commonly used antifungals, highlights the genes that are related to resistance mechanisms of these fungi, which will be a valuable tool for identifying causes of failure of treatments.

## Introduction

Some members of the genera *Fusarium* and *Neocosmospora* are well known as human and plant pathogens [1]. In recent decades, human fusariosis cases have increased, becoming the second most common infections caused by molds after aspergillosis [2]. The illnesses caused by these fungi range from superficial to more severe and invasive, and disseminated infections that affect both immunocompetent and immunocompromised patients [3]. The main species responsible for fusariosis infections belong to the *F. solani* species complex (FSSC), recently reclassified into the *Neocosmospora* genus [4] and *F. oxysporum* species complex (FOSC) [5, 6]. These infections are very difficult to manage and show poor therapeutic response. The survival rate is very low, particularly in patients who have persistent neutropenia [7]. The general conditions of the host and the broad spectrum of antifungal resistance of *Fusarium* are factors that explain the clinical resistance. As a therapeutic management, voriconazole and lipid formulations of amphotericin B (AMB) are the recommended drugs for fusariosis treatment [8]. Nevertheless, other antifungals, such as itraconazole (ITC) and posaconazole (PSC) are also used, mainly in refractory cases or in patients with an intolerance to the other therapies [9, 10].

Currently, it is known that the cytochrome P450 lanosterol 14-α demethylase (*EGR11 syn Cyp51*) is the target of azoles. This enzyme is responsible for the oxidative removal of the 14α-methyl group of lanosterol, an essential step in ergosterol biosynthesis [2]. Ergosterol, which is not in human cells, is an important compound of the fungal cell membrane. This molecule is the target for polyenes like AMB. When the AMB binds to ergosterol, the membrane is weakened and then causes a pore formation that generates an ionic imbalance and oxidative damage, and subsequent cell death [2, 8].

Several resistance mechanisms have been identified in fungal pathogens [11]. For the last antifungal compound, resistance has been linked to the production of enzymes, such as catalase and dehydrogenases, in response to oxidative stress [12], as well as SNPs and mutations in *ERG3* and *ERG6* genes [13], which lead to a lower synthesis of ergosterol and, as a result, therapeutic target loss [14, 15].

In azole resistance, chromosome duplications, promoter modifications and mutations in the *CYP51* gene have been reported in both molds and yeasts [16–18]. These changes in the gene that codes for the therapeutic target, implies an increase in the lanosterol synthesis for molds and modifications in yeasts. In several species of *Fusarium*, including *F. oxysporum*, the presence of three *CYP51* paralogues was found, with *CYP51c* being unique for this genus [19]. In addition, the presence of efflux pumps, such as those of the major facilitator superfamily (MFS) and the superfamily of the ATP-binding cassette (*MDR syn. ABC*) transporters, has been associated with azole resistance [14, 20, 21]. This resistance mechanism aims to reduce the intracellular antifungal and thus avoid reaching the lethal dose. In *Fusarium*, *s*everal putative *ABC* transporters and associated proteins [22] as well as efflux pumps are useful for expulsing xenobiotics [23] and antifungal compounds, especially azoles [1]. Furthermore, the enzyme produced in response to oxidative stress has been reported to be peroxidase [24, 25].

To improve the knowledge of the molecular mechanisms of antifungal resistance in *Fusarium* and related genera, we aimed to identify the differentially expressed genes (DEGs) in two reference pathogenic strains, i.e. *F. oxysporum* FMR 9788 and *N. solani* FMR 4391 (formerly *F. solani*), both exposed to AMB and PSC, to detect genes previously associated with resistance to these compounds in other human and plant pathogenic fungi.

## Methods

### Fungal strains

Two previously identified fungal strains were used in this study: *N. solani* (formerly *F. solani*), clinical (FMR 4391 human blood, USA) [26] and *F. oxysporum*, agricultural (FMR 9788=NRRL 25429=CBS 174.30 Plant, *Gossypium hirsutum*, Egypt) [27]. The susceptibility profiles reported previously (Table S1, available in the online version of this article) were used to determine the sublethal concentrations of the antifungals that were tested in this study (AMB and PSC). These concentrations corresponded to half of the MIC and were used to induce changes in the transcriptional profiles without causing the death of the fungal strains. The strains were provided by the culture collection at the Medicine Faculty of Reus (FMR; Spain) and deposited in the Grupo de Investigación Celular y Molecular de Microorganismos Patógenos (CeMoP), Departamento de Ciencias Biológicas, as well as conserved in the Museo de Historia Natural of Universidad de los Andes, Colombia.

### Culture conditions and inoculum production

Strains were grown on Potato Dextrose Agar (PDA; Oxoid, Basingstoke, UK) at 25 °C for 10 days. Cultures were flooded in a sterile saline solution and filtered to remove cellular and mycelial clumps. The conidial suspensions were adjusted to the desired concentration of 1×10^5^ conidia ml^−1^ using a Neubauer camera. The conidial concentration and viability of the inoculum were verified by subsequent serial plating on PDA plates.

### Antifungal treatments with AMB and PSC

A suspension of 10^5^ conidia ml^−1^ was inoculated into 50 ml of yeast peptone glucose broth (YPD; yeast extract 10 g l^−1^, peptone 10 g l^−1^, dextrose 20 g l^−1^) and incubated at 25 °C for 7 days with agitation at 150 r.p.m. [28]. Mycelia were then collected by filtration and incubated in fresh YPD broth supplemented with sublethal concentrations of each antifungal. For AMB concentrations of 0.5 mg l^−1^ and 1 mg l^−1^ were used for *N. solani* and *F. oxysporum* respectively, while for PSC 8 mg l^−1^ concentrations were used for both fungi, which corresponded to half of the MIC (Table S1).

Antifungals were diluted to obtain a stock solution of 1600 mg l^−1^ in DMSO (Merck, Darmstadt, Germany), which was stored at −20 °C. For the control treatment (NCT), YPG broth was used, supplemented with DMSO to a final concentration of 1 %. Samples were then incubated for 48 h with the antifungals, as previously described. Each treatment was carried out in triplicate (technical replicates), and the whole experiment was repeated three times (biological replicates). Technical replicates were pooled before RNA extraction, which was performed independently for each of the biological replicates.

### Total RNA extraction of mycelia

Mycelia were collected by filtration and macerated in liquid nitrogen using a mortar and pestle and the total RNA was then extracted using the hot acidic phenol method [29]. The quantity and purity of nucleic acids were determined using a NanoDrop ND-1000 UV-VIS spectrophotometer (Thermo Scientific, MA, USA), agarose gel electrophoresis, and a BioAnalyzer 2100 (Agilent Technologies, CA, USA).

### RNA sequencing

RNA sequencing for each sample was performed at BGI on an Illumina HiSeq 2000 instrument using paired-end tags and strand-specific chemistry. A total of 18 libraries were constructed to identify the gene expression profiles of *N. solani* FMR 4391 and *F. oxysporum* FMR 9788 when exposed to AMB or PSC. These libraries included three replicates of (i) *N. solani* and *F. oxysporum* isolates exposed to amphotericin B; (ii) *N. solani* and *F. oxysporum* isolates exposed to posaconazole; and (iii) *N. solani* and *F. oxysporum* isolates exposed to DMSO as a negative control.

To visually inspect the raw reads, we performed quality control using FastQC [30]. Reads were then clipped, quality-trimmed and quality-filtered (with a minimum read length of 60 bps and a quality threshold of 20) using FLEXBAR [31]. After the clean-up process, we used TopHat2 [32] to map all paired-end tag (PET) reads to the corresponding reference genomes. More specifically, reads from *Fusarium oxysporum*. sp. *lycopersici* strain 4287 were mapped to the current version of the genome [33], and *N. solani* reads were mapped to the latest version of the genomes of *Nectria haematococca* (currently *Neocosmospora haematococca*) MPVI isolates 77-13-4 (FGSC 9596, Fungal Genetics Stock Center) and 77-13-7 [34].

Considering the quality of the sequences, two independent replicas of each condition were used to perform all further analyses. Expression levels were presented as fragments per kilobase of exon per million reads (FPKMs), and differential gene-expression analyses were performed using the CuffDiff2 pipeline [35, 36]. Differentially expressed genes between the treated samples (PSC and AMB) and the control (DMSO) were calculated using a false discovery rate (FDR)≤10^−3^, an absolute fold change value (log_2_) ≥2 and a statistical significance threshold of *P*-value<0.05. The pooled method was used for cross-replicate dispersion estimation.

### Data analysis

The database obtained from RNA-seq analyses from each treatment were used to determine gene-ontology (GO) mapping, annotation, enrichment analysis and functional interpretation. First, we filtered in the base the genes with a statistically significant log_2_ fold change (*P*-value<0.005), DEGs with –inf or inf values were replaced for the lower or higher log2 value of each treatment adding+1 or –1, respectively. Next, we introduced the list of loci associated with each DEG into both the blast2GO application [37] and into the OmicsBox Base Platform version 1.2.4 (https://www.biobam.com/omicsbox/?cn-reloaded=1) in order to perform GO and obtained the GO terms. To avoid redundancy REVIGO (http://revigo.irb.hr) [38] was used with a default parameter (allowed similarity=0.7). Next, that data was used to analyse associations and to determine which genes were shared among treatments via a Venn diagram from the online tool http://bioinformatics.psb.ugent.be/webtools/Venn/. The heatmap containing only the DEGs associated with resistance mechanisms, was constructed using R studio software (http://www.rstudio.org/) and the stat package ggplot2 (http://ggplot2.org).

### cDNA synthesis and qRT-PCR

Total RNA was treated with DNase I (Thermo Scientific, MA, USA) and reverse-transcribed using the iScript cDNA Synthesis Kit under the thermal cycling conditions recommended by the manufacturer (BioRad, CA, USA). cDNA samples were stored at −80 °C for subsequent quantitative real-time polymerase chain reaction (qRT-PCR) analysis.

qRT-PCR assays were performed to validate the data obtained by RNA-seq analysis. Three DEGs were selected that had been detected by RNA-seq analysis in *F. oxysporum* when exposed to AMB and PSC treatments. The genes for which expression was assessed included the C-22 sterol desaturase (*ERG5*), gluconate 5 dehydrogenase (*G5D*) and aflatoxin pump efflux (*AFLT*). These genes have been linked to antifungal resistance in other fungi [2, 39–42]. Elongation factor 1 beta (EF1b) was used as the reference (normalizing) gene, the primer sequences of which are described in Table S2.

The QuantPrime platform [31] was used to design the qRT-PCR primers. Primers were designed with an annealing temperature of 60±0.5 °C and to span an exon-exon region border to avoid genomic DNA amplification. All primers were synthesized by IDT (Integrated DNA Technologies, IA, USA). The primer sequences used are shown in Table S2. All selected genes were amplified using the Maxima SYBR Green/ROX qPCR Master Mix (Thermo Scientific, MA, USA). The qRT-PCR was carried out in a 7500 Fast Real-Time PCR System (Applied Biosystems, MA, USA). To calculate the cycle threshold (Ct), the reactions were made as follows: 50 °C for 2 min; 95 °C for 10 min; and 40 cycles of 95 °C for 15 s, 60 °C for 1 min and 72 °C for 20 s values. This process was followed by 95 °C for 15 s, 60 °C for 1 min, 95 °C for 30 s, and then 60 °C for 15 s to obtain melt curves that would assess primer specificity. Each qRT-PCR was prepared in at least three replicates, and the amplification efficiency of each primer set was determined based on ﬁvefold dilutions of the cDNA template.

The Pearson correlation coefficient was calculated for the Ct value of the qRT-PCR analysis and the log2 values. The Ct values for the target genes and the reference genes were compared to those in the control and treated samples and normalized relative to the Ct values obtained for the reference gene using the Relative Expression Software Tool 2009 (REST) (http://rest-2009.gene-quantification.info/). The mathematical model accounted for differences in the amplification efficiencies of the reference gene and the target gene and for the mean Ct deviation between the control and the treated conditions [43].

The expression ratio results were tested for significance by running a Pair Wise Reallocation Randomization Test with α=0.001 using the REST 2009 software [43].

## Results

**Fig. 1.**
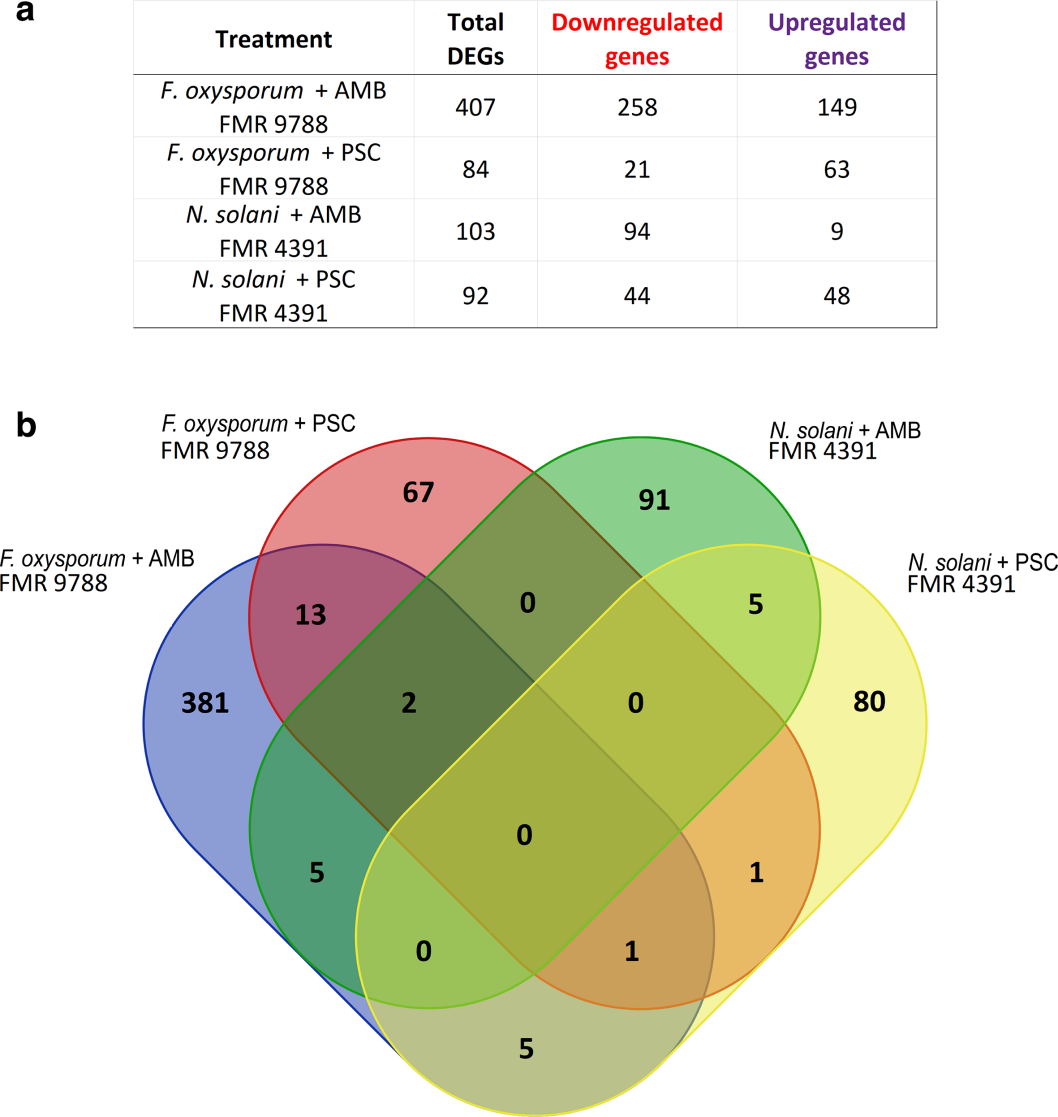
Distribution of genes expressed in response to antifungal treatments. (a) Number of total DEGs and the corresponding number of up- and down-regulated genes in each treatment for *F. oxysporum* FMR 9788 and *N. solani* FMR 4391 after 48 h of exposure to AMB and PSC compared to levels in the negative control. (b) Venn diagram showing the number of differentially expressed and shared transcripts per species and treatment.

The transcriptional profiles of both *F. oxysporum* FMR 9788 and *N. solani* (formerly *F. solani*) FMR 4391 were analysed to identify genes that might be involved in resistance to PSC or AMB. For both strains, RNA was extracted after 48 h of exposure to the antifungal drugs (or DMSO, negative control), and the resulting reads were mapped to the latest version of their respective reference genomes (as described in Methods). The set of statistical results from our RNA-seq data after the blast2Go analysis is shown in Table S3 and the raw reads are available at https://www.ncbi.nlm.nih.gov/geo/query/acc.cgi?acc=GSE82060. As shown in Fig. 1a, we identified several DEGs in the transcriptome of *F. oxysporum* treated with AMB (407 genes) and PSC (84 genes) and in the transcriptome of *N. solani* when treated with AMB (103) and PSC (92 genes), which are given in Table S4. For the DEGs that are probably associated to antifungal resistance, we show the gene name, location and fold change with *P*-value<0.05 in Table S5.

### Initial analysis revealed significant differences among antifungal treatments and fungi

**Fig. 2.**
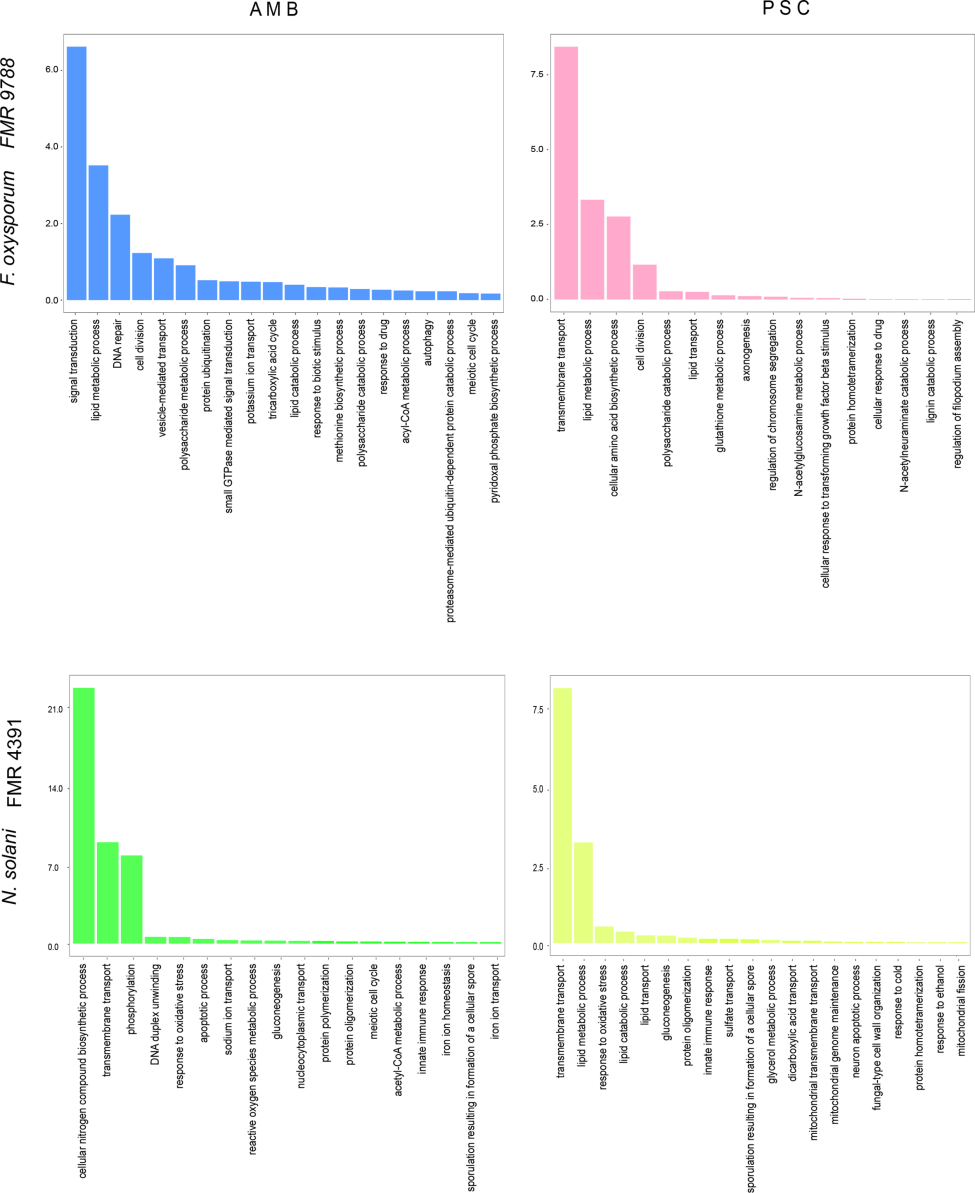
GO based on functional categorization of the DEGs for each treatment and fungus. The significantly enriched (*P*-value < 0.05) GO categories are represented, containing the DEGs expressed by *F. oxysporum* FMR 9788 and *N. solani* FMR 4391 in response to AMB and PSC. On the x-axis is the respective GO erm (biological process) and, on the y-axis, the number of sequences in each GO term.

A total of 686 DEGs were identified in the two strains in response to the two antifungals (Fig. 1a, b), with a global result of 269 up-regulated genes and 417 down-regulated (Fig. 1b). To ascertain the molecular mechanisms involved in the response to these antifungals, we examined the functional distributions of the regulated genes using blast2GO and OmicsBox. GO enrichment analysis allowed us to identify the functional categories under strong regulation upon exposure to the antifungals. The 686 DEGs, identified through RNA-seq analysis, were represented in a wide variety of biological process terms such as lipid metabolic process, lipid catabolic process, lipid transport, transmembrane transport, vesicle-mediated transport, glutathione metabolic process, response to stress oxidative and response to drug (Fig. 2). These are considered of interest because several genes involved in these biological processes have already been associated with antifungal resistance mechanisms in clinical and/or agricultural fungi [14, 15, 23].

**Fig. 3.**
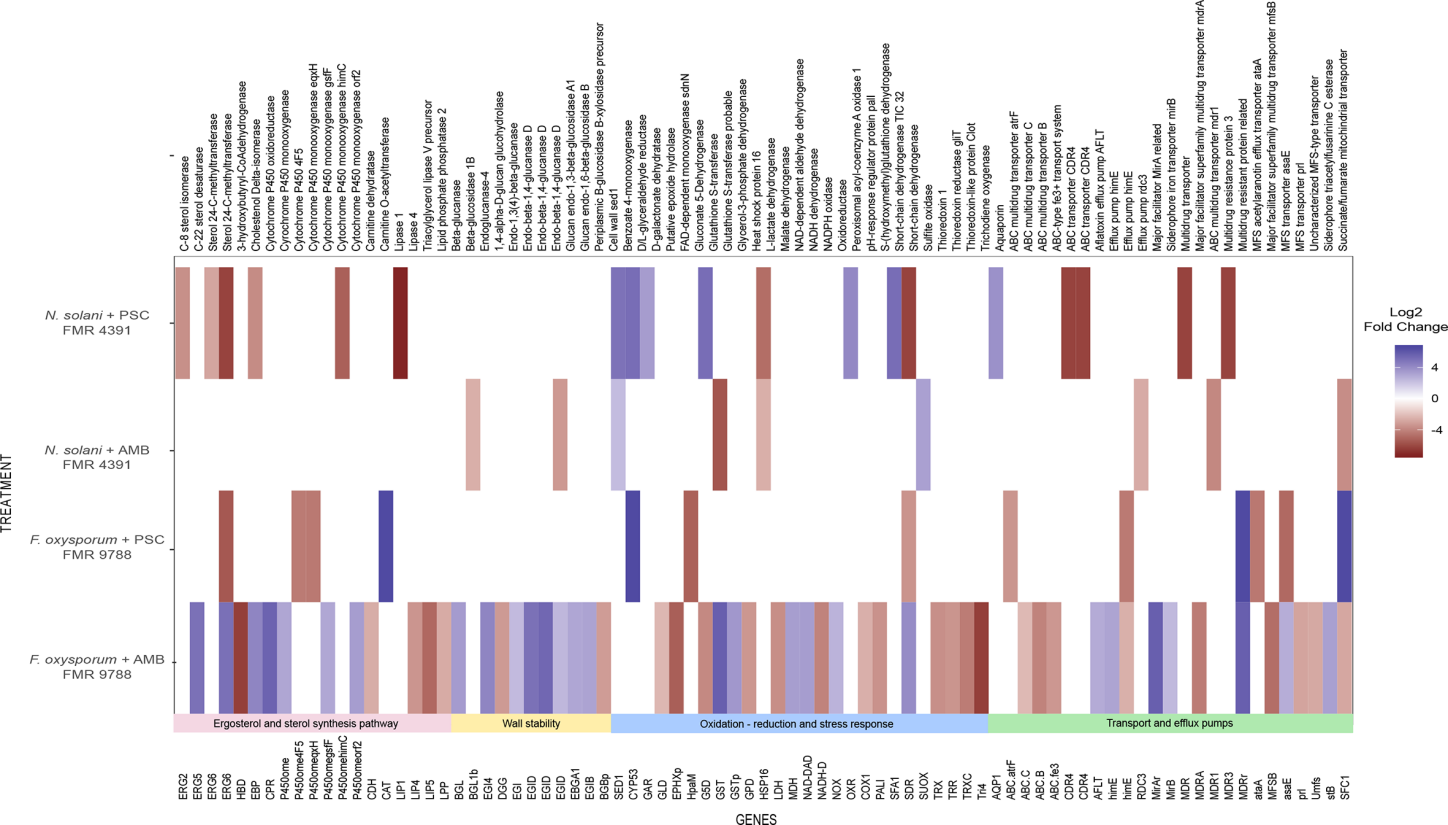
Set of genes that belong to the most representative antifungal resistance categories with their respective genes expressed after exposure of *F. oxysporum* FMR 9788 and *N. solani* FMR 4391 to AMB and PSC (log2-fold change). Values are coloured from violet (up-regulated) to red (down-regulated) according to the colour scale.

Our results also show that the glutathione S-transferase (*GST*) gene was differentially regulated in AMB treatments, although in *F. oxysporum* it was significantly up-regulated while in *N. solani* it was down-regulated (Fig. 3, Table S5). This is a multifunctional protein related to detoxification processes and tolerance to oxidative stress and may be associated with the response to the damage caused by this antifungal tested. In the PSC treatments this gene remained unchanged.

In addition, *F. oxysporum* in the AMB treatment exhibited a significant change in the regulation of genes involved in lipid biosynthetic processes and ergosterol pathway, among which the sterol 24-C-methyltransferase (*ERG 6*), C-22 sterol desaturase (*ERG 5*), cholestenol Delta-isomerase and different cytochrome P450 oxidoreductases genes were up-regulated (Fig. 3).

The AMB-treated and PSC-treated *F. oxysporum* shared 13 DEGs (Fig. 1b), of which an efflux pump himE, a probable glutathione S-transferase and two MFS transporter were of most relevance. For *N. solani* treatments (AMB and PSC) five DEGs were shared, with cell-wall protein SED1 and heat-shock protein 16 (*HSP16*) being the most important (Fig. 3). In both AMB treatments (for *F. oxysporum* and *N. solani*) five DEGs were shared, being mainly associated to putative proteins. Between the two PSC treatments (for *F. oxysporum* and *N. solani*), only the benzoate 4-monooxygenase gene was shared. Interestingly, sterol 24-C-methyltransferase (*ERG6*) was common to *F. oxysporum* AMB, PSC and *N. solani* PSC treatments.

### Genes differentially expressed in *F. oxysporum* and *N solani* against AMB and PSC previously associated with resistance mechanisms in other fungi

#### Genes involved in the ergosterol synthesis pathway, sterol synthesis or related to membrane stability

In total, 26 DEGs were found in *F. oxysporum* for these pathways, 22 in response to AMB and 4 to PSC treatments. For the latter, all genes were down-regulated and encode for cytochrome P450 proteins, *ERG6* and carnitine O-acetyltransferase. In total for this category in the *N. solani* treatments, eight DEGs had transcriptional changes, two in the AMB treatment and the others in the PSC treatment, all of which were down-regulated. For the AMB treatment, the down-regulated genes are related to membrane stability.

In *F. oxysporum* with AMB treatment, some genes related to ergosterol biosynthesis, such as sterol 24-C-methyltransferase (*ERG6*), C-22 sterol desaturase (*ERG5*), cytochrome P450 oxidoreductase (*CPR*) and three cytochrome P450 monooxygenase isoforms were up-regulated, as well as other lipid synthesis related genes, such as cholestenol delta-isomerase and several related to glucan. For this specific treatment, genes like 3-hydroxybutyryl-CoA dehydrogenase (*HBD*), Lipase 4 (*LIP4*), triacylglycerol lipase V precursor and lipid phosphate phosphatase 2 were found to be down-regulated (Fig. 3). As mentioned before, in *N. solani* with the PSC treatment the DEGs were down-regulated and correspond to one isoform of sterol 24-C-methyltransferase (*ERG6*), C-8 sterol isomerase (*ERG2*), cytochrome P450 monooxygenase, cholestenol Delta-isomerase and lipase 4 (*LIP4*) (Fig. 3). As with *F. oxysporum, N. solani* did not have any ergosterol pathway gene up-regulated in the PSC treatment.

Notably, in *F. oxysporum* exposed to AMB*,* we found several up-regulated genes that were related to wall stability, such as the beta-glucanase (*BGL*), glucan endo-1,3-beta-glucosidase A1 (*EBGA1*), endo-beta-1,4-glucanase D (*EGID*) and glucan endo-1,6-beta-glucosidase B (*EGIB*). All of these genes can be related with resistance to antifungals (Fig. 3).

### Genes involved in oxidation-reduction reactions and response to stress

We found that the gene that encodes for the *GST* enzyme was differentially expressed in the AMB treatments. In the case of *F. oxysporum,* it was up-regulated, while in *N. solani* it was down-regulated.

Eighteen genes involved in oxidation-reduction reactions and response to stress were differentially expressed in *F. oxysporum* against AMB and three against PSC treatments. For the latter, two genes were down-regulated and encode one FAD-dependent monooxygenase (*HpaM*), trichodiene oxygenase (*Tri4*) and one encodes a short-chain dehydrogenase, while the benzoate 4-monooxygenase was up-regulated. In AMB treatment, most DEGs were down-regulated, such as thioredoxin (*TRX*), gluconate 5-dehydrogenase (*G5D*), pro-apoptotic serine protease (*PASP*), epoxide hydrolase (*EPHX*), peroxisomal acyl-coenzyme A oxidase 1(*ACOX1*) and short-chain dehydrogenase (*SDR*). Among the up-regulated genes in the AMB treatment were glutathione S-transferase (*GST*), NADPH oxidase (*NOX*), and NAD-dependent aldehyde dehydrogenase (*NAD-DAD*) (Fig. 3).

For this category, *N. solani* treated with AMB had four DEGS, the cell-wall SED1 (*SED1*) and sulfite oxidase genes were up-regulated. In this treatment important genes were down-regulated, such as glutathione S transferase (*GST*) and heat-shock protein 16 (*HSP16*) (Fig. 3). For this fungus under PSC treatment eight genes had transcriptional changes, the benzoate 4-monooxygenase cytochrome P450 (*CYP53*), gluconate 5-dehydrogenase (*G5D*), S-(hydroxymethyl) glutathione dehydrogenase (*GTSp*), D/L-glyceraldehyde reductase (*GLD1*) and cell wall SED1 (SED*1*) genes were up-regulated, while the heat-shock protein 16 (*HSP16*) and short-chain dehydrogenase genes were down-regulated.

### Genes involved in the transport of substances and efflux pumps

After PSC treatment, *F. oxysporum* showed four down-regulated genes: ABC multidrug transporter, efflux pumps himE (*himE*) and two MFS transporters (*MFS*), and only two were up-regulated: multidrug-resistant protein-related genes and fumarate mitochondrial transporter genes. For *F. oxysporum* against AMB, seven genes were up-regulated, the most important being the aflatoxin efflux pump (*AFLT*), efflux pump himE (*himE*), MFS transporter (*MFS*), major facilitator *mirA* and *mirB* related genes; nine genes were down-regulated, among which there were several isoforms of *MFS* and ABC multidrug transporters.

In addition, *N. solani* under AMB treatment had just three DEGs, corresponding to the ABC multidrug transporter (*MDR1*) gene, efflux pump and succinate/fumarate mitochondrial transporters, which were down-regulated. The only up-regulated gene in *N. solani* in this category was an aquaporin in the PSC treatment (Fig. 3); another four genes were down-regulated.

### Validation of gene expression by qRT-PCR

High-throughput paired-end RNA sequencing (RNA-seq) technique is a powerful tool for quantifying and identifying mRNA expression profiles. Despite that being widely accepted [44, 45] we have corroborated our results by qRT-PCR, which is the gold standard for quantifying the level of expression of genes under different conditions [46]. We conducted qRT-PCR on ERG5, G5D and AFLT as gene makers normalized to the elongation factor 1 beta (EF1b) reference gene. The resulting expression levels agreed with the RNA-seq gene expression levels, confirming the obtained results from high-throughput sequencing (Table 1).

**Table 1.** Validation of differentially expressed genes using qRT-PCR. Three genes modulated in response to AMB and PSC were amplified in *F. oxysporum*. Gene expression levels are represented as a log2-fold change at each treatment relative to the negative control (DMSO), as determined by ANOVA followed by Tukey’s post-hoc test (*P*<0.005). The genes are cytochrome P450 oxidoreductase (CPR), gluconate 5 dehydrogenase (G5D), aflatoxin pump efflux (AFLT)

Treatment	Analysis	ERG5	G5D	AFLT
*F. oxysporum* FMR 9788+AMB	RNA-seq	+5.04242	−3.84802	+2.8714
	qRT-PCR	+17.076	−5.760	+4.543
*F. oxysporum* FMR 9788+PSC	RNA-seq	=	=	=
	qRT-PCR	=	=	=

+Up-regulated gene.

˗Down-regulated gene.

=gene without differential expression as determined by RNA-seq analysis and/or qRT-PCR.

## Discussion

After mapping the RNA-seq results to the reference genomes we noticed a low mapping rate of reads of *N. solani*, possibly because the two compared species, *N. haematococca* and *N. solani,* are phylogenetically distant [4, 47]. That result and the remarkable genomic differences in the transcriptomic profiles of *N. solani* in response to antifungal treatments in comparison to *F. oxysporum* support the divergence of the two genera, as has already been reported based on phylogenetic analyses and morphological characters [4, 48].

Several biological processes and genes previously linked to resistance mechanisms in other fungi such as *A. fumigatus*, *Candida albicans* and *Cryptococcus neoformans* [49], were differentially expressed in our treatments. We found unexpected, important negative regulation or no changes in genes related to ergosterol biosynthesis in most treatments. We were expecting an up-regulation of the *ERG11* gene against PSC treatment like other fungal pathogens but did not find any up-regulated gene related to ergosterol synthesis. However, in *F. oxysporum* against AMB, we found *ERG5, ERG6* and some cytochrome P450 monooxygenase isoforms up-regulated compared to controls. Interestingly, the *ERG6* gene was down-regulated for *F. oxysporum* and *N. solani* under PSC treatment.

The up-regulation of *ERG6* has been reported in clinical isolates of *C. parasilopsis* that are resistant to fluconazole; however, in isolates resistant to ITC and AMB, this gene was negatively regulated, mainly associated to a specific missense mutation [50]. Interestingly, the enzyme encoded by *ERG6* is not present in the cholesterol biosynthetic pathway in mammals, suggesting that it might be a good candidate for targeting fungal drugs [51].

The *ERG5* gene is a member of the cytochrome P450 enzymes family [52] and has been described as a catalytically self-sufficient fatty acid, hydroxylase membrane-bound in *F. oxysporum* [53]*,* which catalyses the subterminal (omega-1 to omega-3) hydroxylation of fatty acids. As mentioned for *ERG6*, the *ERG5* gene product is exclusively recovered in the membrane fraction of the fungal cells [54]. In *S. cerevisiae* mutant strains with a deletion of this gene, the ergosterol biosynthesis decreases fourfold and strains showed a major susceptibility to ketoconazole (member of the azole family), indicating that *ERG5* would participate in the azole resistance mechanism [55].

Considering that several cytochrome P450 members are important for ergosterol synthesis and virulence [56], it would be interesting to evaluate through knockout assays the role of *ERG5* and *ERG6* for these fungi as a possible therapeutic targets or to know their role in antifungal resistance, taking into account membrane binding and their function in the ergosterol pathway. Likewise, for both genes, experiments are needed to evaluate their potential involvement in antifungal resistance and therapeutic targetting.

In general, the negative regulation of ergosterol genes does not agree with the transcriptomic profiles obtained from other clinically relevant fungi in which those genes of ergosterol synthesis pathways are generally up-regulated when exposed to azoles, i.e. *A. fumigatus* [57]*, Candida albicans* [39] and *Trichophyton rubrum* [58]. We hypothesize that repressing ergosterol biosynthesis in *F. oxysporum* and *N. solani* against azole treatment might be a mechanism for reducing the expression of the therapeutic target and thus decreasing the damage caused by antifungal agents in the environment [59].

As we mentioned, our study has revealed a small number of DEGs related to sterol synthesis for *F. oxysporum* during AMB treatment, which might be because AMB does not primarily target the ergosterol biosynthetic pathway. However, it has been suggested that in fungi this compound might provoke changes in some ergosterol biosynthesis genes, as occurs in *A. fumigatus* and *C. albicans* in response to AMB [2, 60]. Some researchers have proposed that the down-regulation of ergosterol biosynthesis genes in response to AMB and some azoles like voriconazole is due to an alternate use of sterols or sterol intermediates in the cell membrane [42]. Young *et al*. reported down-regulation of the *ERG3* gene in clinical AMB-resistant isolates of *C. lusitaniae* [15], and Vincent *et al*. showed that the AMB MIC of *C. albicans* increased more than threefold by deleting ergosterol-related genes such as *ERG2, ERG3, ERG6* and *ERG11* [61].

It is important to mention that cell-wall-related genes such as glucan-associated synthesis were up-regulated in *F. oxysporum* after AMB treatment. Glucan is an important compound for the integrity and stability of the cell wall and it is also a unique component to fungi, which makes this cell-wall component an attractive drug target [62].

In response to the stress and oxidative damage caused by the action of AMB [12], *F. oxysporum* had an up-regulation of genes related with detoxification processes, response and tolerance to oxidative stress, such as NADPH oxidase, glutathione S-transferase, and NAD-dependent aldehyde dehydrogenase [63]. This upregulated expression profile is comparable to data reported for *A. fumigatus* in response to AMB treatment [42], in which the main change was in several heat-shock proteins that were up-regulated [41]. However, thioredoxin-like proteins were down-regulated in our AMB treatment. These enzymes modulate the AMB response in *A. fumigatus* to offset the oxidative damage caused by the drug [42]. Additionally, thioredoxin has been reported to be involved in *C. albicans* pathogenesis [64] as well as in *A. fumigatus* [65].

Concerning the genes involved in the stress response, we identified that *F. oxysporum* exposed to PSC showed two genes that encode short-chain dehydrogenase and FAD-dependent monooxygenase that were down-regulated, while *N. solani* exposed to PSC showed that the genes encoding l-glyceraldehyde reductase, oxidoreductase and cell wall *SED1* were up-regulated. This pattern was different in *Cryptococcus neoformans* in response to fluconazole, where superoxide dismutase and nitric oxide dioxygenase genes were up-regulated [66]. The above results suggest that in *F. oxysporum,* the antioxidant enzyme production or the oxidative stress response are not the main mechanisms for the adaptation to the environmental changes caused by PSC exposure. A similar inference can be made in the case of *N. solani* in both antifungal treatments.

Interestingly, the benzoate 4-monooxygenase encoding gene (*CYP53*) exhibited an important up-regulation in the two fungi treated with PSC. This gene encodes the CYP450 enzymes family involved in the phenolic detoxification [67] and it is widely distributed in relevant pathogenic fungi such as *A. fumigatus, A. niger, Rhodothorula minuta* and *Gibberella zeae* [67, 68]. This enzyme family is unique in the fungi kingdom as it does not have homologues in higher eukaryotes. It might, therefore, be a possible candidate drug target against pathogenic fungi. *Cochliobolus lunatus* had been evaluated previously against different natural antifungal phenolic compounds and showed an inhibition of the *CYP53A15* activity and of fungal growth [69]. The present study seems to indicate that, at an early stage in response to PSC treatment, efflux pumps are not an important resistance mechanism for *F. oxysporum* and *N. solani* as we did not observe an increased expression of the main transporter genes previously linked to resistance in fungal pathogens. This contrasts with previous studies on azole responses in other human and plant pathogenic fungi, which suggested drug tolerance in *Fusarium* was due to up-regulation of *ABC* transporters [41] as an effective efflux mechanism [23, 70] thus reducing the intracellular concentrations of the azole, i.e. *Fusarium graminearum* exposed to tebuconazole expressed a total of 54 putative *ABC* transporter proteins [22].

However, our study demonstrated important changes in several efflux pumps genes in their transcriptional expression levels in *F. oxysporum* in response to AMB. This is a striking phenomenon because efflux pumps have never so far been related to AMB resistance in fungi, although they have indeed been identified in parasite pathogens. Purkait *et al*. reported that AMB-resistant *Leishmania* strains displayed an increase in the level of expression of *MDR1*, a member of the ATP-binding cassette (*ABC*) [71]. Specific studies need to be developed that will corroborate the relationship of these efflux pumps as possible mediators of *F. oxysporum* resistance or perhaps increased tolerance when exposed to AMB.

Considering all findings, the present study highlights three main facts. Firstly, from a clinical perspective, it is important to determine the role of the *ERG5, E*RG6 and *CYP53* genes in *Fusarium* spp. that are exposed to antifungal compounds in order to consider them as possible therapeutic targets. Secondly, in the context of novel studies, it is necessary to evaluate, corroborate, characterize and analyse the role of MFS efflux pumps in *F. oxysporum* and their possible role in resistance to AMB. Thirdly, there is a marked difference in the transcriptional response between the two species studied.

Finally, considering that mutations in *ERG3*, *ERG6* and *ERG11* have been linked to AMB and azole resistance in other fungi [1], we can suggest that the presence of one or several point mutations in these strains might be causing the negative regulation of the ergosterol biosynthetic pathway. We consider it important to carry out genomic analyses in order to corroborate, or not, this hypothesis.

## Supplementary Data

Supplementary material 1

Supplementary material 2
