# Supplementary material for: Transcriptional response of Fusarium oxysporum and Neocosmospora solani challenged with amphotericin B or posaconazole
Source: Microbiology (Reading). 2020 Jul 9;166(10):936–46. doi: 10.1099/mic.0.000927 (PMC7660915; doi:10.1099/mic.0.000927)
Supplement: Supplementary material 1 [file mic-166-936-s001.pdf]

Table S1. Susceptibility profile of *F. oxysporum* FMR 9788 (FOSC) and *N. solani* FMR 4391. Sublethal concentrations of amphotericin B (AMB) and posaconazole (PSC) were used in our assays. This concentration corresponded to half of the minimal inhibitory concentration (MIC). *[27], **[28]

Strain	Antifungal compound	MIC mg L^{-1}	Sublethal concentration mg L^{-1}
N. solani FMR 4391*	Amphotericin B (AMB)	1	0.5
	Posaconazole (PSC)	>16	8
F. oxysporum FMR 9788**	Amphotericin B (AMB)	2	1
	Posaconazole (PSC)	>16	8

Table S2. List of primers used for the qRT-PCR assays of *Fusarium oxysporum* FMR 9788. Elongation factor 1-beta (*EF1b*), C-22 sterol desaturase (*ERG5*), Gluconate 5-dehydrogenase (*G5D*) and Aflatoxin efflux pump (*AFLT*).

Gene	ID	Forward Primer Sequence	Reverse Primer Sequence
EF1b	FOXG_00385	CACCCGCTCTTACATTGTTGGC	AGCCTTGAAGGTAGCGACATCAG
ERG5	FOXG_07910	GAGCAGAAGAAGAGCGGAATGC	TAAAGTGAGGCAGGACGACCAC
G5D	FOXG_15642	CCTGGCTACATGCTTACTGCTCTC	AGTCCACTTCGCTTTCAGATCAGG
AFLT	FOXG_03003	TCTGGAAGGGTGATCAGGGAACTC	TGGATGGCCTGGAAGTAAAGAGAG

Table S3. Statistical analysis of RNA sequencing results of six libraries from F. oxysporum FMR 9788 (FOSC) and N. solani FMR 4391 after 48 hours of exposure to amphotericin B (AMB), posaconazole (PSC) or dimethyl-sulfoxide (DMSO) as a negative treatment control (NTC).

Species	Treatment	Biological replica	Clean reads (PE)	Mapped reads (PE)	% Mapping rate	Coverage (X)
F. oxysporum FMR 9788	NTC	R1	30731031	21637143	70.41%	60
		R2	33794104	16899724	50.01%	47
	AMB	R1	30202959	18496776	61.24%	51
		R2	31203947	19610171	62.85%	54
	PSC	R1	26795126	19723619	73.61%	54
		R2	27028589	17420958	64.45%	48
N. solani FMR 4391	NTC	R1	28607293	6856611	23.97%	23
		R2	24968283	4967315	19.89%	17
	AMB	R1	26196526	4448258	16.98%	15
		R2	28956215	5108048	17.64%	17
	PSC	R1	24119498	3897937	16.16%	13
		R2	28937391	6308779	21.80%	21

Table S4: Results of RNA-seq related with the genes differentially expressed for *F. oxysporum* and *N. solani* against AMB or PSC treatment with p-value <0.05.

***F. oxysporum* + AMB**

test_id	gene_id	gene	locus	sample_1	sample_2	status	value_1	value_2	log2(fold_change)	test_stat	p_value	q_value	significant
XLOC_000140	XLOC_000140	-	jgi|Fusox2|10296|FOXG_13277T0:43-591	FOXAMP	FOXNTC	OK	3.56887	0	-6.38819	-nan	5e-05	0.00322127	yes
XLOC_001553	XLOC_001553	-	jgi|Fusox2|14217|FOXG_14591T0:1127-1504	FOXAMP	FOXNTC	OK	4.10796	0	-6.38820	-nan	5e-05	0.00322127	yes
XLOC_003660	XLOC_003660	-	jgi|Fusox2|20487|FOXG_04314T0:1509-2331	FOXAMP	FOXNTC	OK	4.84913	0	-6.38820	-nan	5e-05	0.00322127	yes
XLOC_004700	XLOC_004700	-	jgi|Fusox2|2341|FOXG_01240T0:450-884	FOXAMP	FOXNTC	OK	4.04796	0	-6.38820	-nan	5e-05	0.00322127	yes
XLOC_005700	XLOC_005700	-	jgi|Fusox2|26265|FOXG_07860T0:14-881	FOXAMP	FOXNTC	OK	1.56937	0	-6.38820	-nan	0.0003	0.0128851	yes
XLOC_008455	XLOC_008455	-	jgi|Fusox2|8230|FOXG_11967T0:5960-6582	FOXAMP	FOXNTC	OK	1.70064	0	-6.38820	-nan	0.0003	0.0128851	yes
XLOC_000184	XLOC_000184	-	jgi|Fusox2|10391|FOXG_13345T0:747-1001	FOXAMP	FOXNTC	OK	0	4.60749	5.71096	-nan	0.0013	0.0343208	yes
XLOC_001135	XLOC_001135	-	jgi|Fusox2|12776|FOXG_02574T0:287-468	FOXAMP	FOXNTC	OK	0	20.9095	5.71096	-nan	0.00075	0.0248075	yes
XLOC_001818	XLOC_001818	-	jgi|Fusox2|15313|FOXG_15331T0:4-1024	FOXAMP	FOXNTC	OK	0	3.66514	5.71096	-nan	0.0002	0.00959222	yes
XLOC_001889	XLOC_001889	-	jgi|Fusox2|15458|FOXG_15433T0:169-975	FOXAMP	FOXNTC	OK	0	1.86951	5.71096	-nan	0.0017	0.0398807	yes
XLOC_001927	XLOC_001927	-	jgi|Fusox2|15542|FOXG_21930T0:459-648	FOXAMP	FOXNTC	OK	0	23.0631	5.71096	-nan	0.0004	0.0156964	yes
XLOC_001988	XLOC_001988	-	jgi|Fusox2|15816|FOXG_15501T0:1453-1765	FOXAMP	FOXNTC	OK	0	6.48764	5.71096	-nan	0.00065	0.0223564	yes
XLOC_002094	XLOC_002094	-	jgi|Fusox2|16210|FOXG_15756T0:3486-4346	FOXAMP	FOXNTC	OK	0	1.525	5.71096	-nan	0.0003	0.0128851	yes
XLOC_002192	XLOC_002192	-	jgi|Fusox2|16469|FOXG_02767T0:1207-1835	FOXAMP	FOXNTC	OK	0	4.51296	5.71096	-nan	0.0004	0.0156964	yes
XLOC_002900	XLOC_002900	-	jgi|Fusox2|18176|FOXG_22202T0:1888-2153	FOXAMP	FOXNTC	OK	0	3.443	5.71096	-nan	0.0019	0.0427154	yes
XLOC_002991	XLOC_002991	-	jgi|Fusox2|18904|FOXG_16521T0:546-1008	FOXAMP	FOXNTC	OK	0	1.84779	5.71096	-nan	0.00095	0.028676	yes
XLOC_003026	XLOC_003026	-	jgi|Fusox2|18978|FOXG_16580T0:2-750	FOXAMP	FOXNTC	OK	0	1.83352	5.71096	-nan	5e-05	0.00322127	yes
XLOC_003163	XLOC_003163	-	jgi|Fusox2|19443|FOXG_22612T0:0-312	FOXAMP	FOXNTC	OK	0	10.47	5.71096	-nan	0.0004	0.0156964	yes
XLOC_003195	XLOC_003195	-	jgi|Fusox2|19512|FOXG_22629T0:62-353	FOXAMP	FOXNTC	OK	0	37.8737	5.71096	-nan	5e-05	0.00322127	yes
XLOC_003443	XLOC_003443	-	jgi|Fusox2|20019|FOXG_04034T0:939-1476	FOXAMP	FOXNTC	OK	0	1.43366	5.71096	-nan	0.0017	0.0398807	yes
XLOC_003454	XLOC_003454	-	jgi|Fusox2|20045|FOXG_04058T0:875-1226	FOXAMP	FOXNTC	OK	0	6.16887	5.71096	-nan	5e-05	0.00322127	yes
XLOC_003516	XLOC_003516	-	jgi|Fusox2|20159|FOXG_04132T0:531-630	FOXAMP	FOXNTC	OK	0	77.1579	5.71096	-nan	0.00175	0.0405034	yes
XLOC_003656	XLOC_003656	-	jgi|Fusox2|20483|FOXG_04310T0:29-480	FOXAMP	FOXNTC	OK	0	1.99377	5.71096	-nan	0.0011	0.0310337	yes
XLOC_003785	XLOC_003785	-	jgi|Fusox2|20749|FOXG_04490T0:203-1488	FOXAMP	FOXNTC	OK	0	1.91489	5.71096	-nan	5e-05	0.00322127	yes
XLOC_003828	XLOC_003828	-	jgi|Fusox2|20845|FOXG_04571T0:222-330	FOXAMP	FOXNTC	OK	0	42.7078	5.71096	-nan	0.00155	0.0380146	yes
XLOC_003966	XLOC_003966	-	jgi|Fusox2|21488|FOXG_17189T0:20-616	FOXAMP	FOXNTC	OK	0	1.41608	5.71096	-nan	0.0009	0.0278484	yes
XLOC_004076	XLOC_004076	-	jgi|Fusox2|21820|FOXG_04811T0:35-1273	FOXAMP	FOXNTC	OK	0	3.4391	5.71096	-nan	5e-05	0.00322127	yes
XLOC_004091	XLOC_004091	-	jgi|Fusox2|21851|FOXG_04838T0:71-829	FOXAMP	FOXNTC	OK	0	3.43773	5.71096	-nan	5e-05	0.00322127	yes
XLOC_004112	XLOC_004112	-	jgi|Fusox2|21893|FOXG_04873T0:1490-2102	FOXAMP	FOXNTC	OK	0	1.86878	5.71096	-nan	5e-05	0.00322127	yes
XLOC_004167	XLOC_004167	-	jgi|Fusox2|22003|FOXG_18942T0:90-777	FOXAMP	FOXNTC	OK	0	2.64959	5.71096	-nan	5e-05	0.00322127	yes
XLOC_004450	XLOC_004450	-	jgi|Fusox2|22637|FOXG_05310T0:214-585	FOXAMP	FOXNTC	OK	0	10.0074	5.71096	-nan	5e-05	0.00322127	yes
XLOC_004686	XLOC_004686	-	jgi|Fusox2|23386|FOXG_17590T0:31-979	FOXAMP	FOXNTC	OK	0	2.37493	5.71096	-nan	0.00085	0.0266838	yes
XLOC_004705	XLOC_004705	-	jgi|Fusox2|23431|FOXG_17627T0:648-1089	FOXAMP	FOXNTC	OK	0	5.50806	5.71096	-nan	0.0008	0.0258667	yes
XLOC_004715	XLOC_004715	-	jgi|Fusox2|23493|FOXG_05619T0:372-437	FOXAMP	FOXNTC	OK	0	19315.4	5.71096	-nan	5e-05	0.00322127	yes
XLOC_004880	XLOC_004880	-	jgi|Fusox2|2385|FOXG_18057T0:24-590	FOXAMP	FOXNTC	OK	0	1.721	5.71096	-nan	0.0001	0.00560584	yes
XLOC_005288	XLOC_005288	-	jgi|Fusox2|24828|FOXG_17657T0:0-179	FOXAMP	FOXNTC	OK	0	54.4262	5.71096	-nan	5e-05	0.00322127	yes
XLOC_005515	XLOC_005515	-	jgi|Fusox2|25939|FOXG_07643T0:763-1080	FOXAMP	FOXNTC	OK	0	5.2754	5.71096	-nan	0.0021	0.045551	yes
XLOC_005608	XLOC_005608	-	jgi|Fusox2|26072|FOXG_07748T0:3-697	FOXAMP	FOXNTC	OK	0	1.19145	5.71096	-nan	0.00095	0.028676	yes
XLOC_005763	XLOC_005763	-	jgi|Fusox2|26372|FOXG_07932T0:7-604	FOXAMP	FOXNTC	OK	0	2.16073	5.71096	-nan	0.00015	0.00757281	yes
XLOC_006211	XLOC_006211	-	jgi|Fusox2|3163|FOXG_19819T0:2-168	FOXAMP	FOXNTC	OK	0	12.7124	5.71096	-nan	0.00015	0.00757281	yes
XLOC_007102	XLOC_007102	-	jgi|Fusox2|5073|FOXG_09817T0:855-953	FOXAMP	FOXNTC	OK	0	77.9386	5.71096	-nan	0.00125	0.0336176	yes
XLOC_007430	XLOC_007430	-	jgi|Fusox2|5847|FOXG_20286T0:174-537	FOXAMP	FOXNTC	OK	0	4.39606	5.71096	-nan	0.0021	0.045551	yes
XLOC_007592	XLOC_007592	-	jgi|Fusox2|6231|FOXG_20330T0:70-1201	FOXAMP	FOXNTC	OK	0	1.4961	5.71096	-nan	5e-05	0.00322127	yes
XLOC_007737	XLOC_007737	-	jgi|Fusox2|6620|FOXG_10868T0:561-722	FOXAMP	FOXNTC	OK	0	13.1612	5.71096	-nan	0.0016	0.0388	yes
XLOC_007800	XLOC_007800	-	jgi|Fusox2|6769|FOXG_10992T0:76-151	FOXAMP	FOXNTC	OK	0	12600.4	5.71096	-nan	5e-05	0.00322127	yes
XLOC_007891	XLOC_007891	-	jgi|Fusox2|6932|FOXG_20451T0:6-149	FOXAMP	FOXNTC	OK	0	21.9967	5.71096	-nan	0.0003	0.0128851	yes
XLOC_007994	XLOC_007994	-	jgi|Fusox2|7148|FOXG_11234T0:1-1381	FOXAMP	FOXNTC	OK	0	2.88413	5.71096	-nan	5e-05	0.00322127	yes
XLOC_008288	XLOC_008288	-	jgi|Fusox2|7828|FOXG_11649T0:467-1056	FOXAMP	FOXNTC	OK	0	2.15138	5.71096	-nan	0.00015	0.00757281	yes
XLOC_008419	XLOC_008419	-	jgi|Fusox2|8155|FOXG_11896T0:6-960	FOXAMP	FOXNTC	OK	0	4.75312	5.71096	-nan	5e-05	0.00322127	yes
XLOC_008420	XLOC_008420	-	jgi|Fusox2|8155|FOXG_11896T0:1166-1460	FOXAMP	FOXNTC	OK	0	12.1207	5.71096	-nan	5e-05	0.00322127	yes
XLOC_008425	XLOC_008425	-	jgi|Fusox2|8169|FOXG_20630T0:1-1024	FOXAMP	FOXNTC	OK	0	2.66305	5.71096	-nan	5e-05	0.00322127	yes
XLOC_008522	XLOC_008522	-	jgi|Fusox2|8335|FOXG_12047T0:61-896	FOXAMP	FOXNTC	OK	0	1.24533	5.71096	-nan	0.00045	0.0171896	yes
XLOC_008546	XLOC_008546	-	jgi|Fusox2|8390|FOXG_12096T0:614-872	FOXAMP	FOXNTC	OK	0	8.48041	5.71096	-nan	0.00065	0.0223564	yes
XLOC_008629	XLOC_008629	-	jgi|Fusox2|8562|FOXG_12223T0:289-1839	FOXAMP	FOXNTC	OK	0	2.7306	5.71096	-nan	5e-05	0.00322127	yes
XLOC_008631	XLOC_008631	-	jgi|Fusox2|8564|FOXG_12225T0:222-1558	FOXAMP	FOXNTC	OK	0	3.11199	5.71096	-nan	5e-05	0.00322127	yes
XLOC_008635	XLOC_008635	-	jgi|Fusox2|8570|FOXG_12232T0:1-413	FOXAMP	FOXNTC	OK	0	3.92567	5.71096	-nan	5e-05	0.00322127	yes
XLOC_009035	XLOC_009035	-	jgi|Fusox2|9384|FOXG_12990T0:36-1719	FOXAMP	FOXNTC	OK	0	4.43406	5.71096	-nan	5e-05	0.00322127	yes
XLOC_001502	XLOC_001502	-	jgi|Fusox2|14084|FOXG_21557T0:30-737	FOXAMP	FOXNTC	OK	10073	60.1301	-7.38819	-4.56577	0.0001	0.00560584	yes
XLOC_008572	XLOC_008572	-	jgi|Fusox2|8455|FOXG_12143T0:90-1943	FOXAMP	FOXNTC	OK	180.254	1.46749	-6.94054	-6.32795	5e-05	0.00322127	yes
XLOC_000463	XLOC_000463	-	jgi|Fusox2|11093|FOXG_18156T0:1-649	FOXAMP	FOXNTC	OK	5781.46	51.6352	-6.80694	-5.94353	5e-05	0.00322127	yes
XLOC_001137	XLOC_001137	-	jgi|Fusox2|12781|FOXG_02577T0:4-1164	FOXAMP	FOXNTC	OK	1.15663	121.17	6.71096	4.72969	0.0003	0.0128851	yes
XLOC_000600	XLOC_000600	-	jgi|Fusox2|11419|FOXG_01826T0:0-672	FOXAMP	FOXNTC	OK	7.1355	726.37	6.66955	4.206	5e-05	0.00322127	yes
XLOC_001934	XLOC_001934	-	jgi|Fusox2|15569|FOXG_21944T0:1-1130	FOXAMP	FOXNTC	OK	170.908	1.73383	-6.62311	-4.46776	0.00145	0.0363891	yes
XLOC_008142	XLOC_008142	-	jgi|Fusox2|7494|FOXG_11450T0:67-2300	FOXAMP	FOXNTC	OK	1121.76	11.8529	-6.56437	-4.76784	5e-05	0.00322127	yes
XLOC_001503	XLOC_001503	-	jgi|Fusox2|14085|FOXG_14484T0:18-2172	FOXAMP	FOXNTC	OK	99.3779	1.16232	-6.41785	-6.02	5e-05	0.00322127	yes
XLOC_001989	XLOC_001989	-	jgi|Fusox2|15816|FOXG_15501T0:1981-2445	FOXAMP	FOXNTC	OK	7.56538	554.656	6.19604	5.05533	5e-05	0.00322127	yes
XLOC_005757	XLOC_005757	-	jgi|Fusox2|26356|FOXG_07919T0:85-1513	FOXAMP	FOXNTC	OK	616.643	8.92493	-6.11045	-5.45587	5e-05	0.00322127	yes
XLOC_006710	XLOC_006710	-	jgi|Fusox2|4231|FOXG_09265T0:25-816	FOXAMP	FOXNTC	OK	345.638	5.78074	-5.90187	-5.03381	5e-05	0.00322127	yes
XLOC_008511	XLOC_008511	-	jgi|Fusox2|8310|FOXG_12024T0:8-1662	FOXAMP	FOXNTC	OK	0.607169	30.3203	5.64204	4.5544	5e-05	0.00322127	yes
XLOC_000220	XLOC_000220	-	jgi|Fusox2|10478|FOXG_13404T0:0-2270	FOXAMP	FOXNTC	OK	205.803	4.19239	-5.61735	-4.61502	5e-05	0.00322127	yes
XLOC_002667	XLOC_002667	-	jgi|Fusox2|17537|FOXG_03403T0:28-1308	FOXAMP	FOXNTC	OK	232.31	4.78089	-5.60263	-4.51643	5e-05	0.00322127	yes
XLOC_007002	XLOC_007002	-	jgi|Fusox2|4904|FOXG_09685T0:12-1658	FOXAMP	FOXNTC	OK	2.60687	125.127	5.58492	4.54075	5e-05	0.00322127	yes
XLOC_000115	XLOC_000115	-	jgi|Fusox2|10257|FOXG_21114T0:12-813	FOXAMP	FOXNTC	OK	15732.9	332.586	-5.56391	-3.61418	5e-05	0.00322127	yes
XLOC_008320	XLOC_008320	-	jgi|Fusox2|7895|FOXG_11698T0:1-1073	FOXAMP	FOXNTC	OK	1.14521	48.456	5.40299	4.36929	0.0003	0.0128851	yes
XLOC_002197	XLOC_002197	-	jgi|Fusox2|16478|FOXG_02777T0:0-2751	FOXAMP	FOXNTC	OK	5253.98	125.33	-5.3896	-3.45679	5e-05	0.00322127	yes
XLOC_007882	XLOC_007882	-	jgi|Fusox2|690|FOXG_17862T0:18-1135	FOXAMP	FOXNTC	OK	94.3175	2.27988	-5.37049	-4.36129	5e-05	0.00322127	yes
XLOC_005512	XLOC_005512	-	jgi|Fusox2|25935|FOXG_07639T0:89-2324	FOXAMP	FOXNTC	OK	356.493	8.9619	-5.31392	-5.14025	5e-05	0.00322127	yes
XLOC_008378	XLOC_008378	-	jgi|Fusox2|8047|FOXG_11813T0:0-1262	FOXAMP	FOXNTC	OK	385.508	9.82591	-5.29403	-4.47256	5e-05	0.00322127	yes
XLOC_002995	XLOC_002995	-	jgi|Fusox2|18910|FOXG_16527T0:111-879	FOXAMP	FOXNTC	OK	1561.7	41.0822	-5.24846	-4.87689	5e-05	0.00322127	yes
XLOC_004881	XLOC_004881	-	jgi|Fusox2|23866|FOXG_05898T0:404-2561	FOXAMP	FOXNTC	OK	21.0576	762.282	5.17791	3.57936	5e-05	0.00322127	yes
XLOC_006377	XLOC_006377	-	jgi|Fusox2|3510|FOXG_08749T0:3-673	FOXAMP	FOXNTC	OK	88.8725	2.55194	-5.12207	-4.30292	0.0004	0.0156964	yes
XLOC_007069	XLOC_007069	-	jgi|Fusox2|5024|FOXG_09779T0:28-2013	FOXAMP	FOXNTC	OK	48.1419	1.41266	-5.09081	-4.14076	5e-05	0.00322127	yes
XLOC_001838	XLOC_001838	-	jgi|Fusox2|15352|FOXG_15360T0:0-184	FOXAMP	FOXNTC	OK	1082.17	31.8009	-5.08872	-3.71729	5e-05	0.00322127	yes
XLOC_006388	XLOC_006388	-	jgi|Fusox2|3538|FOXG_08765T0:120-1569	FOXAMP	FOXNTC	OK	1.03418	34.8751	5.07564	4.21162	5e-05	0.00322127	yes
XLOC_006974	XLOC_006974	-	jgi|Fusox2|4848|FOXG_09647T0:248-1628	FOXAMP	FOXNTC	OK	9.92179	326.972	5.04242	4.26974	5e-05	0.00322127	yes
XLOC_006822	XLOC_006822	-	jgi|Fusox2|4473|FOXG_09410T0:11-423	FOXAMP	FOXNTC	OK	806.983	25.3173	-4.99434	-4.7816	5e-05	0.00322127	yes
XLOC_006532	XLOC_006532	-	jgi|Fusox2|3882|FOXG_09027T0:8-1770	FOXAMP	FOXNTC	OK	309.702	9.90342	-4.96681	-4.0039	5e-05	0.00322127	yes
XLOC_002749	XLOC_002749	-	jgi|Fusox2|17746|FOXG_03522T0:6-3103	FOXAMP	FOXNTC	OK	0.637107	19.7767	4.95612	3.78994	5e-05	0.00322127	yes
XLOC_004109	XLOC_004109	-	jgi|Fusox2|21886|FOXG_04868T0:37-1317	FOXAMP	FOXNTC	OK	1.0367	31.9526	4.94586	3.88879	0.00105	0.0302155	yes
XLOC_004523	XLOC_004523	-	jgi|Fusox2|22848|FOXG_05414T0:4-1916	FOXAMP	FOXNTC	OK	563.515	18.3713	-4.93893	-4.75548	5e-05	0.00322127	yes
XLOC_004509	XLOC_004509	-	jgi|Fusox2|22825|FOXG_19051T0:0-1980	FOXAMP	FOXNTC	OK	285.216	9.42375	-4.91961	-4.91373	5e-05	0.00322127	yes
XLOC_003504	XLOC_003504	-	jgi|Fusox2|20138|FOXG_04119T0:6-1198	FOXAMP	FOXNTC	OK	3.80272	112.856	4.89131	3.86805	5e-05	0.00322127	yes
XLOC_007273	XLOC_007273	-	jgi|Fusox2|5427|FOXG_10105T0:141-1059	FOXAMP	FOXNTC	OK	80.6684	2.93959	-4.77832	-4.46272	5e-05	0.00322127	yes
XLOC_002135	XLOC_002135	-	jgi|Fusox2|16296|FOXG_15830T0:0-1981	FOXAMP	FOXNTC	OK	0.942228	25.7352	4.77152	4.4035	5e-05	0.00322127	yes
XLOC_006253	XLOC_006253	-	jgi|Fusox2|3249|FOXG_08562T0:0-2855	FOXAMP	FOXNTC	OK	126.514	4.64223	-4.76834	-4.86835	5e-05	0.00322127	yes
XLOC_005881	XLOC_005881	-	jgi|Fusox2|2655|FOXG_08222T0:0-789	FOXAMP	FOXNTC	OK	81.363	3.11531	-4.70693	-3.84831	0.00045	0.0171896	yes
XLOC_001505	XLOC_001505	-	jgi|Fusox2|14095|FOXG_21564T0:5-378	FOXAMP	FOXNTC	OK	512.806	19.8328	-4.69246	-4.36088	0.00015	0.00757281	yes
XLOC_006927	XLOC_006927	-	jgi|Fusox2|4743|FOXG_09565T0:4-1326	FOXAMP	FOXNTC	OK	880.338	34.6948	-4.66527	-4.12396	5e-05	0.00322127	yes
XLOC_006633	XLOC_006633	-	jgi|Fusox2|4044|FOXG_09149T0:0-1047	FOXAMP	FOXNTC	OK	2.16475	53.5018	4.62731	3.95189	0.0005	0.0184466	yes
XLOC_001822	XLOC_001822	-	jgi|Fusox2|15319|FOXG_15337T0:44-4046	FOXAMP	FOXNTC	OK	67.0447	2.79816	-4.58257	-4.57423	5e-05	0.00322127	yes
XLOC_000154	XLOC_000154	-	jgi|Fusox2|10319|FOXG_13296T0:35-1976	FOXAMP	FOXNTC	OK	0.386707	9.15726	4.5656	3.50339	0.0021	0.045551	yes
XLOC_007788	XLOC_007788	-	jgi|Fusox2|6731|FOXG_10964T0:1508-1809	FOXAMP	FOXNTC	OK	23.3695	545.229	4.54416	3.28813	5e-05	0.00322127	yes
XLOC_006725	XLOC_006725	-	jgi|Fusox2|4250|FOXG_09281T0:165-1988	FOXAMP	FOXNTC	OK	2.33909	52.0153	4.47491	3.84813	5e-05	0.00322127	yes
XLOC_002262	XLOC_002262	-	jgi|Fusox2|16609|FOXG_02878T0:143-1340	FOXAMP	FOXNTC	OK	1.74006	38.2785	4.45932	3.78392	5e-05	0.00322127	yes
XLOC_007075	XLOC_007075	-	jgi|Fusox2|5031|FOXG_09785T0:37-1692	FOXAMP	FOXNTC	OK	0.823598	17.606	4.41799	3.45415	0.00165	0.0395679	yes
XLOC_000161	XLOC_000161	-	jgi|Fusox2|10336|FOXG_13309T0:0-1631	FOXAMP	FOXNTC	OK	27.8322	1.30413	-4.41559	-3.65159	0.00095	0.028676	yes
XLOC_008447	XLOC_008447	-	jgi|Fusox2|8217|FOXG_11954T0:2212-3011	FOXAMP	FOXNTC	OK	60.3494	2.87833	-4.39004	-4.0164	5e-05	0.00322127	yes
XLOC_004229	XLOC_004229	-	jgi|Fusox2|22161|FOXG_05035T0:2-821	FOXAMP	FOXNTC	OK	92.0113	4.44746	-4.37076	-3.66611	5e-05	0.00322127	yes

XLOC_006254	XLOC_006254	-	jgi|Fusox2|3251|FOXG_19831T0:0-303	FOXAMP	FOXNTC	OK	404.563	19.7491	-4.35651	-3.23995	5e-05	0.00322127	yes
XLOC_002198	XLOC_002198	-	jgi|Fusox2|16479|FOXG_02778T0:0-672	FOXAMP	FOXNTC	OK	406.551	20.22	-4.32958	-4.47274	5e-05	0.00322127	yes
XLOC_008612	XLOC_008612	-	jgi|Fusox2|8527|FOXG_12199T0:66-873	FOXAMP	FOXNTC	OK	38.4481	1.92305	-4.32145	-3.7729	5e-05	0.00322127	yes
XLOC_000007	XLOC_000007	-	jgi|Fusox2|10028|FOXG_13070T0:3-1993	FOXAMP	FOXNTC	OK	147.635	7.44006	-4.31058	-4.30918	5e-05	0.00322127	yes
XLOC_008446	XLOC_008446	-	jgi|Fusox2|8217|FOXG_11954T0:0-2148	FOXAMP	FOXNTC	OK	33.1643	1.67184	-4.31013	-4.10829	5e-05	0.00322127	yes
XLOC_000360	XLOC_000360	-	jgi|Fusox2|10838|FOXG_01504T0:232-1532	FOXAMP	FOXNTC	OK	42.3301	2.14738	-4.30104	-3.54527	0.0009	0.0278484	yes
XLOC_002199	XLOC_002199	-	jgi|Fusox2|16480|FOXG_02779T0:0-320	FOXAMP	FOXNTC	OK	5556.76	284.2	-4.28927	-3.63823	5e-05	0.00322127	yes
XLOC_007056	XLOC_007056	-	jgi|Fusox2|5001|FOXG_09760T0:8-2201	FOXAMP	FOXNTC	OK	1632.41	83.7597	-4.2846	-3.61363	5e-05	0.00322127	yes
XLOC_008966	XLOC_008966	-	jgi|Fusox2|9227|FOXG_12873T0:7-998	FOXAMP	FOXNTC	OK	6.52173	127.043	4.28392	4.00675	5e-05	0.00322127	yes
XLOC_003505	XLOC_003505	-	jgi|Fusox2|20139|FOXG_04120T0:4-2343	FOXAMP	FOXNTC	OK	8.06762	156.569	4.27851	4.34495	5e-05	0.00322127	yes
XLOC_003820	XLOC_003820	-	jgi|Fusox2|20820|FOXG_04552T0:1-910	FOXAMP	FOXNTC	OK	97.5597	5.05338	-4.27097	-3.51724	5e-05	0.00322127	yes
XLOC_000218	XLOC_000218	-	jgi|Fusox2|10476|FOXG_13402T0:22-1275	FOXAMP	FOXNTC	OK	86.1489	4.54867	-4.24332	-3.68776	0.0001	0.00560584	yes
XLOC_006350	XLOC_006350	-	jgi|Fusox2|3446|FOXG_08699T0:0-996	FOXAMP	FOXNTC	OK	4407.37	234.312	-4.23342	-3.30963	5e-05	0.00322127	yes
XLOC_002731	XLOC_002731	-	jgi|Fusox2|17715|FOXG_03503T0:27-1343	FOXAMP	FOXNTC	OK	1.51499	28.475	4.23232	3.7592	5e-05	0.00322127	yes
XLOC_008053	XLOC_008053	-	jgi|Fusox2|7303|FOXG_11315T0:79-1520	FOXAMP	FOXNTC	OK	39.3718	2.1105	-4.22151	-3.62421	0.0005	0.0184466	yes
XLOC_001628	XLOC_001628	-	jgi|Fusox2|14496|FOXG_14782T0:30-2978	FOXAMP	FOXNTC	OK	22.9918	1.24866	-4.20266	-3.59161	5e-05	0.00322127	yes
XLOC_005395	XLOC_005395	-	jgi|Fusox2|25763|FOXG_07503T0:58-2324	FOXAMP	FOXNTC	OK	125.476	6.83919	-4.19744	-4.27967	5e-05	0.00322127	yes
XLOC_002996	XLOC_002996	-	jgi|Fusox2|18910|FOXG_16527T0:1123-1902	FOXAMP	FOXNTC	OK	403.253	22.0995	-4.1896	-3.82327	5e-05	0.00322127	yes
XLOC_004862	XLOC_004862	-	jgi|Fusox2|23827|FOXG_05871T0:4-1693	FOXAMP	FOXNTC	OK	0.629021	11.1736	4.15084	3.46134	0.00015	0.00757281	yes
XLOC_008750	XLOC_008750	-	jgi|Fusox2|8803|FOXG_12394T0:38-1337	FOXAMP	FOXNTC	OK	0.886594	15.6614	4.14279	3.35505	0.0001	0.00560584	yes
XLOC_002997	XLOC_002997	-	jgi|Fusox2|18910|FOXG_16527T0:2095-3188	FOXAMP	FOXNTC	OK	1635.54	93.2025	-4.13325	-3.52554	5e-05	0.00322127	yes
XLOC_004919	XLOC_004919	-	jgi|Fusox2|23974|FOXG_05962T0:50-1318	FOXAMP	FOXNTC	OK	239.911	14.0294	-4.09597	-3.80576	5e-05	0.00322127	yes
XLOC_002261	XLOC_002261	-	jgi|Fusox2|16605|FOXG_02876T0:57-3115	FOXAMP	FOXNTC	OK	1.24597	21.133	4.08415	3.51078	5e-05	0.00322127	yes
XLOC_004510	XLOC_004510	-	jgi|Fusox2|22826|FOXG_19052T0:0-1173	FOXAMP	FOXNTC	OK	243.62	14.4119	-4.0793	-4.18655	5e-05	0.00322127	yes
XLOC_006399	XLOC_006399	-	jgi|Fusox2|3565|FOXG_08787T0:4-1486	FOXAMP	FOXNTC	OK	19.1102	1.13867	-4.06892	-3.63514	5e-05	0.00322127	yes
XLOC_006376	XLOC_006376	-	jgi|Fusox2|3509|FOXG_08748T0:2-1245	FOXAMP	FOXNTC	OK	32.5393	1.94	-4.06805	-3.47309	0.00055	0.0199502	yes
XLOC_003521	XLOC_003521	-	jgi|Fusox2|20171|FOXG_18750T0:88-1794	FOXAMP	FOXNTC	OK	35.3079	2.12846	-4.05211	-3.32448	0.0009	0.0278484	yes
XLOC_005951	XLOC_005951	-	jgi|Fusox2|26697|FOXG_08148T0:83-1749	FOXAMP	FOXNTC	OK	0.501314	8.25368	4.04125	2.75891	0.0021	0.045551	yes
XLOC_003798	XLOC_003798	-	jgi|Fusox2|20783|FOXG_04516T0:18-1157	FOXAMP	FOXNTC	OK	0.863167	13.9663	4.01616	3.23406	0.00025	0.0112409	yes
XLOC_007679	XLOC_007679	-	jgi|Fusox2|6445|FOXG_10729T0:19-1110	FOXAMP	FOXNTC	OK	2034.79	126.212	-4.01096	-2.93232	0.0004	0.0156964	yes
XLOC_003792	XLOC_003792	-	jgi|Fusox2|20772|FOXG_04507T0:0-954	FOXAMP	FOXNTC	OK	3.46669	55.4735	4.00017	3.30704	5e-05	0.00322127	yes
XLOC_000486	XLOC_000486	-	jgi|Fusox2|1115|FOXG_17899T0:1705-3055	FOXAMP	FOXNTC	OK	252.254	15.7881	-3.99797	-4.03284	5e-05	0.00322127	yes
XLOC_001506	XLOC_001506	-	jgi|Fusox2|14096|FOXG_21565T0:0-534	FOXAMP	FOXNTC	OK	851.756	53.5583	-3.99126	-3.8306	0.0001	0.00560584	yes
XLOC_007073	XLOC_007073	-	jgi|Fusox2|5030|FOXG_09783T0:13-1653	FOXAMP	FOXNTC	OK	3.19637	50.5824	3.98413	3.47828	5e-05	0.00322127	yes
XLOC_004689	XLOC_004689	-	jgi|Fusox2|23400|FOXG_17601T0:97-1631	FOXAMP	FOXNTC	OK	10.1631	0.647271	-3.97283	-3.04486	0.00125	0.0336176	yes
XLOC_004627	XLOC_004627	-	jgi|Fusox2|23090|FOXG_05542T0:43-1847	FOXAMP	FOXNTC	OK	36.1378	2.31036	-3.96732	-3.36949	5e-05	0.00322127	yes
XLOC_008669	XLOC_008669	-	jgi|Fusox2|8674|FOXG_12292T0:85-1288	FOXAMP	FOXNTC	OK	0.761357	11.8188	3.95636	2.91735	0.0008	0.0258667	yes
XLOC_001839	XLOC_001839	-	jgi|Fusox2|15352|FOXG_15360T0:300-1734	FOXAMP	FOXNTC	OK	709.7	46.3053	-3.93796	-3.5451	5e-05	0.00322127	yes
XLOC_003821	XLOC_003821	-	jgi|Fusox2|20823|FOXG_04555T0:114-972	FOXAMP	FOXNTC	OK	7.41678	111.496	3.91005	3.05358	0.00015	0.00757281	yes
XLOC_005486	XLOC_005486	-	jgi|Fusox2|25900|FOXG_07611T0:1-3342	FOXAMP	FOXNTC	OK	295.574	19.6761	-3.909	-3.47306	5e-05	0.00322127	yes
XLOC_006255	XLOC_006255	-	jgi|Fusox2|3252|FOXG_08563T0:1-362	FOXAMP	FOXNTC	OK	272.764	18.7213	-3.8649	-2.95577	0.00015	0.00757281	yes
XLOC_002019	XLOC_002019	-	jgi|Fusox2|16044|FOXG_15642T0:0-1153	FOXAMP	FOXNTC	OK	38.6948	2.68709	-3.84802	-3.30697	0.00035	0.0143883	yes
XLOC_004853	XLOC_004853	-	jgi|Fusox2|23815|FOXG_05861T0:92-1497	FOXAMP	FOXNTC	OK	4.01752	57.4049	3.8368	3.34166	0.0001	0.00560584	yes
XLOC_008177	XLOC_008177	-	jgi|Fusox2|7577|FOXG_11504T0:1-663	FOXAMP	FOXNTC	OK	20.1289	1.42404	-3.8212	-3.14054	0.00065	0.0223564	yes
XLOC_001817	XLOC_001817	-	jgi|Fusox2|15310|FOXG_15329T0:7-1525	FOXAMP	FOXNTC	OK	1.01427	14.2574	3.8132	3.14411	0.00015	0.00757281	yes
XLOC_006667	XLOC_006667	-	jgi|Fusox2|4127|FOXG_09210T0:140-1274	FOXAMP	FOXNTC	OK	20.8871	1.49742	-3.80206	-3.34006	5e-05	0.00322127	yes
XLOC_007165	XLOC_007165	-	jgi|Fusox2|5200|FOXG_09926T0:0-453	FOXAMP	FOXNTC	OK	45.4715	3.30591	-3.78184	-2.81404	0.00075	0.0248075	yes
XLOC_006996	XLOC_006996	-	jgi|Fusox2|4891|FOXG_09678T0:28-1189	FOXAMP	FOXNTC	OK	319.707	23.5238	-3.76456	-3.0543	0.00055	0.0199502	yes
XLOC_008996	XLOC_008996	-	jgi|Fusox2|9285|FOXG_12911T0:167-1619	FOXAMP	FOXNTC	OK	33.2095	444.301	3.74187	3.21964	0.0001	0.00560584	yes
XLOC_002992	XLOC_002992	-	jgi|Fusox2|18905|FOXG_16522T0:5-1230	FOXAMP	FOXNTC	OK	3.65462	48.2882	3.72388	3.46569	5e-05	0.00322127	yes
XLOC_007868	XLOC_007868	-	jgi|Fusox2|6896|FOXG_11071T0:13-1456	FOXAMP	FOXNTC	OK	454.263	34.5521	-3.71668	-3.47198	5e-05	0.00322127	yes
XLOC_008220	XLOC_008220	-	jgi|Fusox2|7694|FOXG_11564T0:116-1771	FOXAMP	FOXNTC	OK	2.78828	36.5189	3.7112	3.17951	5e-05	0.00322127	yes
XLOC_004948	XLOC_004948	-	jgi|Fusox2|24018|FOXG_05995T0:14-3722	FOXAMP	FOXNTC	OK	436.045	33.66	-3.69537	-3.24974	5e-05	0.00322127	yes
XLOC_006400	XLOC_006400	-	jgi|Fusox2|3566|FOXG_08788T0:1-1978	FOXAMP	FOXNTC	OK	31.8895	2.47815	-3.68575	-3.64646	0.0001	0.00560584	yes
XLOC_008314	XLOC_008314	-	jgi|Fusox2|7884|FOXG_11687T0:50-1457	FOXAMP	FOXNTC	OK	48.4703	3.78069	-3.68038	-3.6064	5e-05	0.00322127	yes
XLOC_003941	XLOC_003941	-	jgi|Fusox2|21431|FOXG_17148T0:494-1445	FOXAMP	FOXNTC	OK	53.4154	4.19981	-3.66886	-2.94155	0.0001	0.00560584	yes
XLOC_002324	XLOC_002324	-	jgi|Fusox2|16752|FOXG_02980T0:9-2311	FOXAMP	FOXNTC	OK	23.8675	1.8771	-3.66847	-3.50222	5e-05	0.00322127	yes
XLOC_005006	XLOC_005006	-	jgi|Fusox2|24156|FOXG_19178T0:61-2463	FOXAMP	FOXNTC	OK	24.8336	1.95451	-3.66741	-3.18398	0.00045	0.0171896	yes
XLOC_007426	XLOC_007426	-	jgi|Fusox2|5843|FOXG_10378T0:17-924	FOXAMP	FOXNTC	OK	2.54014	31.824	3.64714	2.69322	0.0007	0.023514	yes
XLOC_002859	XLOC_002859	-	jgi|Fusox2|17989|FOXG_03654T0:20-1950	FOXAMP	FOXNTC	OK	939.365	75.0416	-3.64592	-2.56278	0.0018	0.0411095	yes
XLOC_007663	XLOC_007663	-	jgi|Fusox2|6403|FOXG_10698T0:35-1907	FOXAMP	FOXNTC	OK	1335.33	107.031	-3.6411	-2.68103	0.0011	0.0310337	yes
XLOC_007898	XLOC_007898	-	jgi|Fusox2|6947|FOXG_11101T0:3-4355	FOXAMP	FOXNTC	OK	612.87	49.215	-3.63841	-3.00494	0.0007	0.023514	yes
XLOC_000012	XLOC_000012	-	jgi|Fusox2|10036|FOXG_13078T0:16-794	FOXAMP	FOXNTC	OK	2.03776	24.9402	3.61341	2.71667	0.0013	0.0343208	yes
XLOC_003829	XLOC_003829	-	jgi|Fusox2|20845|FOXG_04571T0:394-729	FOXAMP	FOXNTC	OK	6.38515	77.8618	3.60812	2.8423	0.0004	0.0156964	yes
XLOC_006151	XLOC_006151	-	jgi|Fusox2|3029|FOXG_08430T0:1-1659	FOXAMP	FOXNTC	OK	20.6238	250.44	3.60208	3.12162	0.00015	0.00757281	yes
XLOC_003166	XLOC_003166	-	jgi|Fusox2|19450|FOXG_16865T0:50-3918	FOXAMP	FOXNTC	OK	71.3836	5.96285	-3.58152	-3.67415	5e-05	0.00322127	yes
XLOC_004525	XLOC_004525	-	jgi|Fusox2|22849|FOXG_05415T0:550-960	FOXAMP	FOXNTC	OK	30.5996	2.5704	-3.57345	-2.40861	0.00145	0.0363891	yes
XLOC_006973	XLOC_006973	-	jgi|Fusox2|4846|FOXG_09646T0:68-1729	FOXAMP	FOXNTC	OK	25.2425	2.12065	-3.57328	-3.35762	5e-05	0.00322127	yes
XLOC_005934	XLOC_005934	-	jgi|Fusox2|2664|FOXG_08228T0:3-1094	FOXAMP	FOXNTC	OK	1048.7	88.4179	-3.56813	-3.61469	5e-05	0.00322127	yes
XLOC_000282	XLOC_000282	-	jgi|Fusox2|10664|FOXG_13556T0:413-955	FOXAMP	FOXNTC	OK	190.559	16.2574	-3.55107	-3.3064	0.0004	0.0156964	yes
XLOC_004409	XLOC_004409	-	jgi|Fusox2|22548|FOXG_05258T0:0-723	FOXAMP	FOXNTC	OK	531.552	45.5171	-3.54573	-3.64061	5e-05	0.00322127	yes
XLOC_001613	XLOC_001613	-	jgi|Fusox2|14397|FOXG_14733T0:14-1510	FOXAMP	FOXNTC	OK	57.2948	4.92923	-3.53897	-3.50998	0.0001	0.00560584	yes
XLOC_007068	XLOC_007068	-	jgi|Fusox2|5023|FOXG_09778T0:4-999	FOXAMP	FOXNTC	OK	1.75156	19.9646	3.51073	2.65172	0.0019	0.0427154	yes
XLOC_003335	XLOC_003335	-	jgi|Fusox2|19787|FOXG_03855T0:49-4987	FOXAMP	FOXNTC	OK	20.1677	1.77114	-3.50929	-3.38946	0.0001	0.00560584	yes
XLOC_006296	XLOC_006296	-	jgi|Fusox2|3331|FOXG_08617T0:7-2425	FOXAMP	FOXNTC	OK	92.9211	8.20376	-3.50165	-3.6753	5e-05	0.00322127	yes
XLOC_001535	XLOC_001535	-	jgi|Fusox2|14170|FOXG_14550T0:0-2337	FOXAMP	FOXNTC	OK	82.6655	7.30977	-3.49939	-2.99438	0.00115	0.0321293	yes
XLOC_004031	XLOC_004031	-	jgi|Fusox2|21689|FOXG_18888T0:1-1771	FOXAMP	FOXNTC	OK	21.3396	1.88803	-3.49858	-2.93191	0.0017	0.0398807	yes
XLOC_004380	XLOC_004380	-	jgi|Fusox2|22498|FOXG_05230T0:0-1895	FOXAMP	FOXNTC	OK	73.9054	6.65617	-3.47292	-3.41515	5e-05	0.00322127	yes
XLOC_000276	XLOC_000276	-	jgi|Fusox2|10642|FOXG_13541T0:7-2832	FOXAMP	FOXNTC	OK	9.24248	0.834786	-3.4688	-2.93417	0.0012	0.0329924	yes
XLOC_003169	XLOC_003169	-	jgi|Fusox2|19455|FOXG_16869T0:62-845	FOXAMP	FOXNTC	OK	5.97979	66.2005	3.46867	2.95423	5e-05	0.00322127	yes
XLOC_001861	XLOC_001861	-	jgi|Fusox2|15408|FOXG_15398T0:2-2544	FOXAMP	FOXNTC	OK	1428.99	129.949	-3.45897	-2.51281	0.00045	0.0171896	yes
XLOC_001083	XLOC_001083	-	jgi|Fusox2|12626|FOXG_02467T0:325-1292	FOXAMP	FOXNTC	OK	50.6363	4.6349	-3.44956	-2.96507	0.0017	0.0398807	yes
XLOC_002657	XLOC_002657	-	jgi|Fusox2|17528|FOXG_03393T0:21-3430	FOXAMP	FOXNTC	OK	2.62527	28.5881	3.44488	3.5752	5e-05	0.00322127	yes
XLOC_000536	XLOC_000536	-	jgi|Fusox2|11271|FOXG_01740T0:9-4322	FOXAMP	FOXNTC	OK	4.64118	50.301	3.43802	3.44945	5e-05	0.00322127	yes
XLOC_003207	XLOC_003207	-	jgi|Fusox2|19536|FOXG_16927T0:390-1830	FOXAMP	FOXNTC	OK	19.7856	1.82829	-3.43589	-3.237	0.00035	0.0143883	yes
XLOC_004428	XLOC_004428	-	jgi|Fusox2|22584|FOXG_19020T0:2-1218	FOXAMP	FOXNTC	OK	131.927	12.3021	-3.42277	-2.82096	0.00085	0.0266838	yes
XLOC_002721	XLOC_002721	-	jgi|Fusox2|17693|FOXG_03489T0:0-170	FOXAMP	FOXNTC	OK	2260.06	211.142	-3.42008	-3.79338	0.00135	0.0344809	yes
XLOC_004336	XLOC_004336	-	jgi|Fusox2|2237|FOXG_01173T0:0-183	FOXAMP	FOXNTC	OK	88.9459	942.37	3.40529	2.57645	0.00045	0.0171896	yes
XLOC_000045	XLOC_000045	-	jgi|Fusox2|10117|FOXG_13126T0:42-1832	FOXAMP	FOXNTC	OK	0.654597	6.90811	3.39961	2.68825	0.0005	0.0184466	yes
XLOC_002247	XLOC_002247	-	jgi|Fusox2|16587|FOXG_02858T0:89-1682	FOXAMP	FOXNTC	OK	1.69738	17.8687	3.39606	3.19848	0.0002	0.00959222	yes
XLOC_001202	XLOC_001202	-	jgi|Fusox2|12989|FOXG_13653T0:27-4951	FOXAMP	FOXNTC	OK	426.169	40.6088	-3.39156	-2.52262	0.0017	0.0398807	yes
XLOC_000179	XLOC_000179	-	jgi|Fusox2|10374|FOXG_13339T0:9-1590	FOXAMP	FOXNTC	OK	43.2412	4.12597	-3.3896	-2.94945	0.0009	0.0278484	yes
XLOC_007215	XLOC_007215	-	jgi|Fusox2|5305|FOXG_10002T0:17-3122	FOXAMP	FOXNTC	OK	221.975	21.2467	-3.38508	-3.35067	5e-05	0.00322127	yes
XLOC_000285	XLOC_000285	-	jgi|Fusox2|10671|FOXG_13564T0:15-1852	FOXAMP	FOXNTC	OK	66.8872	6.44442	-3.37561	-3.30012	5e-05	0.00322127	yes
XLOC_004672	XLOC_004672	-	jgi|Fusox2|2327|FOXG_01230T0:3-825	FOXAMP	FOXNTC	OK	2.61788	27.0376	3.3685	2.97947	0.00015	0.00757281	yes
XLOC_002162	XLOC_002162	-	jgi|Fusox2|16404|FOXG_02711T0:49-1251	FOXAMP	FOXNTC	OK	191.69	18.5899	-3.36618	-3.59964	5e-05	0.00322127	yes
XLOC_005374	XLOC_005374	-	jgi|Fusox2|25736|FOXG_07483T0:114-1263	FOXAMP	FOXNTC	OK	1.66563	17.1555	3.36454	2.84019	0.0002	0.00959222	yes
XLOC_005275	XLOC_005275	-	jgi|Fusox2|2476|FOXG_01332T0:478-2365	FOXAMP	FOXNTC	OK	608.579	59.2194	-3.3613	-2.65925	0.00125	0.0336176	yes
XLOC_001203	XLOC_001203	-	jgi|Fusox2|12990|FOXG_13654T0:0-715	FOXAMP	FOXNTC	OK	299.23	29.444	-3.34521	-2.77659	0.0013	0.0343208	yes
XLOC_005401	XLOC_005401	-	jgi|Fusox2|25774|FOXG_07515T0:0-771	FOXAMP	FOXNTC	OK	95.4901	9.4029	-3.34417	-3.27828	0.0002	0.00959222	yes
XLOC_007395	XLOC_007395	-	jgi|Fusox2|5795|FOXG_10340T0:28-3225	FOXAMP	FOXNTC	OK	190.79	18.7892	-3.34401	-3.38687	5e-05	0.00322127	yes
XLOC_006104	XLOC_006104	-	jgi|Fusox2|2936|FOXG_08370T0:1-1503	FOXAMP	FOXNTC	OK	144.753	14.289	-3.34062	-3.20055	0.0001	0.00560584	yes
XLOC_009053	XLOC_009053	-	jgi|Fusox2|9433|FOXG_13024T0:18667-21566	FOXAMP	FOXNTC	OK	0.43048	4.30494	3.32198	2.47639	0.0021	0.045551	yes
XLOC_007400	XLOC_007400	-	jgi|Fusox2|5808|FOXG_10348T0:0-978	FOXAMP	FOXNTC	OK	635.257	63.7452	-3.31695	-3.34203	5e-05	0.00322127	yes
XLOC_006630	XLOC_006630	-	jgi|Fusox2|4038|FOXG_09142T0:58-957	FOXAMP	FOXNTC	OK	166.469	1656.64	3.31493	2.85401	0.0012	0.0329924	yes
XLOC_003096	XLOC_003096	-	jgi|Fusox2|1928|FOXG_00993T0:72-3358	FOXAMP	FOXNTC	OK	18.1882	1.84486	-3.30142	-3.22149	0.00025	0.0112409	yes
XLOC_000805	XLOC_000805	-	jgi|Fusox2|12021|FOXG_02115T0:3-1530	FOXAMP	FOXNTC	OK	1522.98	154.612	-3.30017	-2.81959	0.00115	0.0321293	yes
XLOC_002045	XLOC_002045	-	jgi|Fusox2|16120|FOXG_15693T0:9-905	FOXAMP	FOXNTC	OK	451.089	45.9898	-3.29402	-3.57853	5e-05	0.00322127	yes
XLOC_004283	XLOC_004283	-	jgi|Fusox2|22265|FOXG_05101T0:145-1174	FOXAMP	FOXNTC	OK	1726.97	176.89	-3.28731	-2.73793	0.0013	0.0343208	yes
XLOC_008719	XLOC_008719	-	jgi|Fusox2|8753|FOXG_12352T0:0-531	FOXAMP	FOXNTC	OK	2889.7	296.276	-3.2859	-3.29016	5e-05	0.00322127	yes
XLOC_000560	XLOC_000560	-	jgi|Fusox2|11325|FOXG_18180T0:32-1131	FOXAMP	FOXNTC	OK	3.62958	35.2593	3.28013	2.92809	0.0001	0.00560584	yes
XLOC_007071	XLOC_007071	-	jgi|Fusox2|5027|FOXG_09781T0:33-1122	FOXAMP	FOXNTC	OK	1.71298	16.6207	3.2784	2.89066	0.00015	0.00757281	yes
XLOC_007927	XLOC_007927	-	jgi|Fusox2|6999|FOXG_11136T0:0-1517	FOXAMP	FOXNTC	OK	0.78289	7.569	3.27322	2.77107	0.00085	0.0266838	yes

XLOC_000286	XLOC_000286	-	jgi|Fusox2|10676|FOXG_13566T0:41-2337	FOXAMP	FOXNTC	OK	453.679	47.0094	-3.27065	-3.15067	0.0001	0.00560584	yes
XLOC_001825	XLOC_001825	-	jgi|Fusox2|15340|FOXG_15352T0:7-1899	FOXAMP	FOXNTC	OK	6.86906	65.7201	3.25815	3.47257	5e-05	0.00322127	yes
XLOC_008399	XLOC_008399	-	jgi|Fusox2|809|FOXG_17868T0:0-768	FOXAMP	FOXNTC	OK	32.7607	312.714	3.25481	2.69853	0.00135	0.0344809	yes
XLOC_001294	XLOC_001294	-	jgi|Fusox2|13149|FOXG_13770T0:3-574	FOXAMP	FOXNTC	OK	23.3772	223.056	3.25423	2.80914	0.00135	0.0344809	yes
XLOC_001240	XLOC_001240	-	jgi|Fusox2|13055|FOXG_21238T0:99-1624	FOXAMP	FOXNTC	OK	5.75571	0.606223	-3.24707	-2.55416	0.0016	0.0388	yes
XLOC_005040	XLOC_005040	-	jgi|Fusox2|24233|FOXG_19187T0:0-14568	FOXAMP	FOXNTC	OK	7.75889	0.818279	-3.24518	-3.07705	5e-05	0.00322127	yes
XLOC_004555	XLOC_004555	-	jgi|Fusox2|2288|FOXG_18030T0:0-1024	FOXAMP	FOXNTC	OK	135.919	14.3774	-3.24087	-3.47102	5e-05	0.00322127	yes
XLOC_000255	XLOC_000255	-	jgi|Fusox2|10570|FOXG_13480T0:106-1513	FOXAMP	FOXNTC	OK	369.535	39.3436	-3.23151	-3.04232	5e-05	0.00322127	yes
XLOC_000087	XLOC_000087	-	jgi|Fusox2|10206|FOXG_13204T0:26-996	FOXAMP	FOXNTC	OK	634.084	67.5209	-3.23127	-3.14231	0.0002	0.00959222	yes
XLOC_001597	XLOC_001597	-	jgi|Fusox2|14328|FOXG_21603T0:194-519	FOXAMP	FOXNTC	OK	7.28571	68.132	3.22519	2.7682	0.00065	0.0223564	yes
XLOC_004811	XLOC_004811	-	jgi|Fusox2|23700|FOXG_05772T0:595-1099	FOXAMP	FOXNTC	OK	94.0737	10.178	-3.20833	-2.6692	0.00225	0.0481991	yes
XLOC_007241	XLOC_007241	-	jgi|Fusox2|5355|FOXG_10041T0:13-1435	FOXAMP	FOXNTC	OK	4.98586	45.4517	3.18842	2.80433	0.00025	0.0112409	yes
XLOC_002147	XLOC_002147	-	jgi|Fusox2|1637|FOXG_00838T0:6-1142	FOXAMP	FOXNTC	OK	357.646	39.5562	-3.17656	-3.4379	5e-05	0.00322127	yes
XLOC_003422	XLOC_003422	-	jgi|Fusox2|19979|FOXG_04002T0:96-1573	FOXAMP	FOXNTC	OK	95.1917	10.5669	-3.17128	-3.07358	0.00015	0.00757281	yes
XLOC_004526	XLOC_004526	-	jgi|Fusox2|22849|FOXG_05415T0:1060-2267	FOXAMP	FOXNTC	OK	44.3729	4.99057	-3.1524	-2.75419	0.00135	0.0344809	yes
XLOC_006994	XLOC_006994	-	jgi|Fusox2|4889|FOXG_09676T0:99-1112	FOXAMP	FOXNTC	OK	294.661	33.1564	-3.15169	-2.84602	5e-05	0.00322127	yes
XLOC_008214	XLOC_008214	-	jgi|Fusox2|7682|FOXG_11557T0:46-1054	FOXAMP	FOXNTC	OK	29.5597	3.33028	-3.14992	-2.61763	0.00135	0.0344809	yes
XLOC_004499	XLOC_004499	-	jgi|Fusox2|22796|FOXG_05386T0:14-3348	FOXAMP	FOXNTC	OK	6.29619	55.6099	3.14279	2.63316	0.00165	0.0395679	yes
XLOC_004908	XLOC_004908	-	jgi|Fusox2|23954|FOXG_05946T0:0-1315	FOXAMP	FOXNTC	OK	18.4832	2.10472	-3.13451	-2.80392	0.00025	0.0112409	yes
XLOC_002970	XLOC_002970	-	jgi|Fusox2|18857|FOXG_16484T0:0-1256	FOXAMP	FOXNTC	OK	1.4049	12.181	3.11609	2.76888	0.00085	0.0266838	yes
XLOC_006501	XLOC_006501	-	jgi|Fusox2|3812|FOXG_08977T0:7-1171	FOXAMP	FOXNTC	OK	11.3515	1.31153	-3.11357	-2.67497	0.00065	0.0223564	yes
XLOC_005011	XLOC_005011	-	jgi|Fusox2|24170|FOXG_06090T0:20-2403	FOXAMP	FOXNTC	OK	37.5674	4.34224	-3.11297	-3.2668	0.0001	0.00560584	yes
XLOC_007425	XLOC_007425	-	jgi|Fusox2|5842|FOXG_10377T0:0-3285	FOXAMP	FOXNTC	OK	14.0814	1.62784	-3.11275	-2.64867	0.00095	0.028676	yes
XLOC_001395	XLOC_001395	-	jgi|Fusox2|13380|FOXG_21291T0:77-2101	FOXAMP	FOXNTC	OK	12.9584	111.728	3.10803	2.6454	0.0007	0.023514	yes
XLOC_001034	XLOC_001034	-	jgi|Fusox2|12552|FOXG_02406T0:11-2665	FOXAMP	FOXNTC	OK	110.285	12.7977	-3.10728	-2.91575	5e-05	0.00322127	yes
XLOC_008713	XLOC_008713	-	jgi|Fusox2|8741|FOXG_12343T0:17-2802	FOXAMP	FOXNTC	OK	11.771	100.647	3.096	3.02457	0.0002	0.00959222	yes
XLOC_006644	XLOC_006644	-	jgi|Fusox2|4064|FOXG_09165T1:5461-6751	FOXAMP	FOXNTC	OK	9.30793	79.2961	3.09072	2.86393	0.0004	0.0156964	yes
XLOC_002738	XLOC_002738	-	jgi|Fusox2|17729|FOXG_03513T0:1-2854	FOXAMP	FOXNTC	OK	33.2103	280.386	3.07771	2.79198	0.0015	0.0374263	yes
XLOC_006378	XLOC_006378	-	jgi|Fusox2|3513|FOXG_08751T0:0-712	FOXAMP	FOXNTC	OK	529.148	62.9934	-3.0704	-3.32276	5e-05	0.00322127	yes
XLOC_007624	XLOC_007624	-	jgi|Fusox2|6323|FOXG_10637T0:7-1489	FOXAMP	FOXNTC	OK	2.22892	18.5515	3.05712	2.92234	0.0004	0.0156964	yes
XLOC_002663	XLOC_002663	-	jgi|Fusox2|17534|FOXG_03400T0:33-1542	FOXAMP	FOXNTC	OK	150.192	18.1629	-3.04774	-3.22642	5e-05	0.00322127	yes
XLOC_001108	XLOC_001108	-	jgi|Fusox2|12694|FOXG_02513T0:29-1291	FOXAMP	FOXNTC	OK	1.92406	15.8547	3.04269	2.78507	0.00065	0.0223564	yes
XLOC_001270	XLOC_001270	-	jgi|Fusox2|13101|FOXG_13743T0:18-1722	FOXAMP	FOXNTC	OK	274.197	33.3353	-3.04009	-3.21956	5e-05	0.00322127	yes
XLOC_008513	XLOC_008513	-	jgi|Fusox2|8316|FOXG_12028T0:66-1237	FOXAMP	FOXNTC	OK	3.58896	29.0544	3.01712	2.63871	0.00035	0.0143883	yes
XLOC_004751	XLOC_004751	-	jgi|Fusox2|23569|FOXG_19100T0:19-1284	FOXAMP	FOXNTC	OK	23.288	2.90964	-3.00068	-2.89025	0.0005	0.0184466	yes
XLOC_005895	XLOC_005895	-	jgi|Fusox2|26584|FOXG_08080T0:0-3437	FOXAMP	FOXNTC	OK	1243.44	155.517	-2.9992	-2.3388	0.00085	0.0266838	yes
XLOC_000040	XLOC_000040	-	jgi|Fusox2|10109|FOXG_13119T0:25-1275	FOXAMP	FOXNTC	OK	23.5051	186.269	2.98634	2.94373	0.0002	0.00959222	yes
XLOC_003406	XLOC_003406	-	jgi|Fusox2|19944|FOXG_18703T0:0-1203	FOXAMP	FOXNTC	OK	12.1985	95.6508	2.97107	3.16496	5e-05	0.00322127	yes
XLOC_003840	XLOC_003840	-	jgi|Fusox2|20875|FOXG_04598T0:79-1944	FOXAMP	FOXNTC	OK	20.0423	2.56221	-2.96759	-2.91474	0.00065	0.0223564	yes
XLOC_006834	XLOC_006834	-	jgi|Fusox2|4507|FOXG_09428T0:64-1440	FOXAMP	FOXNTC	OK	100.506	12.9137	-2.96031	-3.10766	5e-05	0.00322127	yes
XLOC_007143	XLOC_007143	-	jgi|Fusox2|5141|FOXG_20174T0:5-574	FOXAMP	FOXNTC	OK	196.648	25.4328	-2.95086	-2.7814	0.0021	0.045551	yes
XLOC_008119	XLOC_008119	-	jgi|Fusox2|7440|FOXG_11412T0:0-2787	FOXAMP	FOXNTC	OK	7.0518	0.915403	-2.94551	-2.66204	0.00065	0.0223564	yes
XLOC_001011	XLOC_001011	-	jgi|Fusox2|12499|FOXG_02376T0:242-5735	FOXAMP	FOXNTC	OK	216.974	28.1982	-2.94385	-2.79148	0.0002	0.00959222	yes
XLOC_002303	XLOC_002303	-	jgi|Fusox2|166|FOXG_17802T0:0-757	FOXAMP	FOXNTC	OK	31.9	4.18437	-2.93047	-2.29441	0.00175	0.0405034	yes
XLOC_000742	XLOC_000742	-	jgi|Fusox2|1183|FOXG_00599T0:0-786	FOXAMP	FOXNTC	OK	467.389	61.4761	-2.92653	-3.17466	5e-05	0.00322127	yes
XLOC_002221	XLOC_002221	-	jgi|Fusox2|16527|FOXG_02813T0:0-864	FOXAMP	FOXNTC	OK	5.90685	44.6634	2.91863	2.5922	0.001	0.0294642	yes
XLOC_003687	XLOC_003687	-	jgi|Fusox2|20543|FOXG_04352T0:37-1510	FOXAMP	FOXNTC	OK	36.2404	4.79618	-2.91764	-2.84619	0.00035	0.0143883	yes
XLOC_007824	XLOC_007824	-	jgi|Fusox2|6814|FOXG_11021T0:0-399	FOXAMP	FOXNTC	OK	1269.95	168.792	-2.91145	-3.10748	5e-05	0.00322127	yes
XLOC_007076	XLOC_007076	-	jgi|Fusox2|5031|FOXG_09785T0:9803-10221	FOXAMP	FOXNTC	OK	5.85006	43.9633	2.90978	2.35688	0.00185	0.0420291	yes
XLOC_006373	XLOC_006373	-	jgi|Fusox2|3503|FOXG_19871T0:4917-6946	FOXAMP	FOXNTC	OK	129.794	17.5651	-2.88543	-3.06054	0.00025	0.0112409	yes
XLOC_002993	XLOC_002993	-	jgi|Fusox2|18909|FOXG_16526T0:4-1632	FOXAMP	FOXNTC	OK	81.8062	11.0766	-2.8847	-2.77191	0.00085	0.0266838	yes
XLOC_001923	XLOC_001923	-	jgi|Fusox2|15536|FOXG_15482T0:0-618	FOXAMP	FOXNTC	OK	127.776	17.333	-2.88202	-2.80454	0.0011	0.0310337	yes
XLOC_005509	XLOC_005509	-	jgi|Fusox2|25932|FOXG_07636T0:92-2281	FOXAMP	FOXNTC	OK	238.482	32.3632	-2.88146	-2.94698	0.00015	0.00757281	yes
XLOC_008762	XLOC_008762	-	jgi|Fusox2|8828|FOXG_12648T0:6-1293	FOXAMP	FOXNTC	OK	34.4625	4.67978	-2.88051	-2.82004	0.00065	0.0223564	yes
XLOC_006932	XLOC_006932	-	jgi|Fusox2|4751|FOXG_09572T0:0-1161	FOXAMP	FOXNTC	OK	2.07377	15.1754	2.8714	2.57679	0.0006	0.0215825	yes
XLOC_003003	XLOC_003003	-	jgi|Fusox2|18921|FOXG_16534T0:169-1637	FOXAMP	FOXNTC	OK	72.4134	9.90593	-2.86989	-3.08463	0.00015	0.00757281	yes
XLOC_006956	XLOC_006956	-	jgi|Fusox2|4804|FOXG_09616T0:41-2026	FOXAMP	FOXNTC	OK	144.117	19.8738	-2.8583	-3.08427	5e-05	0.00322127	yes
XLOC_001023	XLOC_001023	-	jgi|Fusox2|12530|FOXG_02390T1:0-632	FOXAMP	FOXNTC	OK	72.255	10.0635	-2.84397	-2.31237	0.0016	0.0388	yes
XLOC_001790	XLOC_001790	-	jgi|Fusox2|15248|FOXG_15276T0:173-1120	FOXAMP	FOXNTC	OK	28.1016	201.662	2.84321	2.93482	5e-05	0.00322127	yes
XLOC_004809	XLOC_004809	-	jgi|Fusox2|236|FOXG_00117T0:0-906	FOXAMP	FOXNTC	OK	12.0601	86.3361	2.83972	2.73776	0.0001	0.00560584	yes
XLOC_001531	XLOC_001531	-	jgi|Fusox2|14165|FOXG_14546T0:11-1292	FOXAMP	FOXNTC	OK	49.5884	6.93113	-2.83884	-2.78866	0.00035	0.0143883	yes
XLOC_005028	XLOC_005028	-	jgi|Fusox2|24211|FOXG_06112T0:0-810	FOXAMP	FOXNTC	OK	254.237	35.8635	-2.82559	-2.90884	5e-05	0.00322127	yes
XLOC_001881	XLOC_001881	-	jgi|Fusox2|15436|FOXG_15424T0:17-3194	FOXAMP	FOXNTC	OK	1.22613	8.64495	2.81774	2.43888	0.00125	0.0336176	yes
XLOC_004233	XLOC_004233	-	jgi|Fusox2|22174|FOXG_05042T0:14-3852	FOXAMP	FOXNTC	OK	10.7159	75.3218	2.81331	2.74324	0.00035	0.0143883	yes
XLOC_003810	XLOC_003810	-	jgi|Fusox2|20805|FOXG_04537T0:2744-5151	FOXAMP	FOXNTC	OK	0.665885	4.67325	2.81108	2.4571	0.0013	0.0343208	yes
XLOC_004153	XLOC_004153	-	jgi|Fusox2|21965|FOXG_04915T0:272-1685	FOXAMP	FOXNTC	OK	2.16323	15.1427	2.80736	2.56844	0.001	0.0294642	yes
XLOC_006083	XLOC_006083	-	jgi|Fusox2|2874|FOXG_08338T0:17-1098	FOXAMP	FOXNTC	OK	146.807	20.9825	-2.80667	-2.83967	0.00025	0.0112409	yes
XLOC_007418	XLOC_007418	-	jgi|Fusox2|5837|FOXG_10372T0:16-1938	FOXAMP	FOXNTC	OK	127.724	18.3953	-2.79562	-2.94241	5e-05	0.00322127	yes
XLOC_001910	XLOC_001910	-	jgi|Fusox2|15508|FOXG_15460T0:15-2472	FOXAMP	FOXNTC	OK	23.3027	3.35952	-2.79417	-2.69631	0.00125	0.0336176	yes
XLOC_003432	XLOC_003432	-	jgi|Fusox2|19993|FOXG_04016T0:16-653	FOXAMP	FOXNTC	OK	42.9784	297.745	2.79239	2.95793	0.0001	0.00560584	yes
XLOC_004816	XLOC_004816	-	jgi|Fusox2|23704|FOXG_05776T0:1-948	FOXAMP	FOXNTC	OK	193.588	28.2993	-2.77415	-2.97402	0.00015	0.00757281	yes
XLOC_003301	XLOC_003301	-	jgi|Fusox2|19714|FOXG_03801T0:7-1287	FOXAMP	FOXNTC	OK	23.3785	3.44465	-2.76276	-2.65092	0.0011	0.0310337	yes
XLOC_003930	XLOC_003930	-	jgi|Fusox2|2133|FOXG_01116T0:0-1414	FOXAMP	FOXNTC	OK	6.8015	45.9772	2.75699	2.35061	0.002	0.0443856	yes
XLOC_008448	XLOC_008448	-	jgi|Fusox2|8217|FOXG_11954T0:3068-7362	FOXAMP	FOXNTC	OK	62.0221	9.19092	-2.7545	-2.85849	0.0001	0.00560584	yes
XLOC_007401	XLOC_007401	-	jgi|Fusox2|5809|FOXG_20283T0:0-816	FOXAMP	FOXNTC	OK	715.82	106.288	-2.75162	-2.74149	5e-05	0.00322127	yes
XLOC_000136	XLOC_000136	-	jgi|Fusox2|10288|FOXG_13269T0:19-1662	FOXAMP	FOXNTC	OK	9.2494	61.8552	2.74146	2.75417	0.0003	0.0128851	yes
XLOC_002690	XLOC_002690	-	jgi|Fusox2|17622|FOXG_03443T0:3-1563	FOXAMP	FOXNTC	OK	25.6165	3.83859	-2.73843	-2.68858	0.00135	0.0344809	yes
XLOC_001382	XLOC_001382	-	jgi|Fusox2|13331|FOXG_13880T0:1-1532	FOXAMP	FOXNTC	OK	53.8689	8.07976	-2.73707	-2.84956	0.0001	0.00560584	yes
XLOC_001629	XLOC_001629	-	jgi|Fusox2|1449|FOXG_00724T0:11-2630	FOXAMP	FOXNTC	OK	547.925	82.8551	-2.72531	-2.57201	0.0007	0.023514	yes
XLOC_003692	XLOC_003692	-	jgi|Fusox2|20549|FOXG_04356T0:78-1409	FOXAMP	FOXNTC	OK	91.3206	13.8795	-2.71799	-2.93935	0.00025	0.0112409	yes
XLOC_007266	XLOC_007266	-	jgi|Fusox2|5415|FOXG_10093T0:15-1617	FOXAMP	FOXNTC	OK	16.0997	104.037	2.69198	2.81823	0.00025	0.0112409	yes
XLOC_008722	XLOC_008722	-	jgi|Fusox2|8764|FOXG_12362T0:89-1532	FOXAMP	FOXNTC	OK	56.9703	8.85779	-2.68519	-2.65665	0.0019	0.0427154	yes
XLOC_008128	XLOC_008128	-	jgi|Fusox2|7457|FOXG_11427T0:154-1837	FOXAMP	FOXNTC	OK	171.868	26.7465	-2.68388	-2.48049	0.0005	0.0184466	yes
XLOC_006417	XLOC_006417	-	jgi|Fusox2|360|FOXG_00183T0:0-1546	FOXAMP	FOXNTC	OK	167.228	26.0283	-2.68367	-2.54959	0.00165	0.0395679	yes
XLOC_004004	XLOC_004004	-	jgi|Fusox2|21634|FOXG_17419T0:10-1541	FOXAMP	FOXNTC	OK	486.522	76.032	-2.67783	-2.51933	0.00065	0.0223564	yes
XLOC_007371	XLOC_007371	-	jgi|Fusox2|5744|FOXG_10309T0:66-3095	FOXAMP	FOXNTC	OK	43.9905	6.89845	-2.67285	-2.80173	5e-05	0.00322127	yes
XLOC_000615	XLOC_000615	-	jgi|Fusox2|1145|FOXG_00575T0:0-708	FOXAMP	FOXNTC	OK	676.205	106.111	-2.67189	-2.50512	0.00035	0.0143883	yes
XLOC_007096	XLOC_007096	-	jgi|Fusox2|5062|FOXG_09809T0:25-2450	FOXAMP	FOXNTC	OK	1.95198	12.3509	2.6616	2.36059	0.0022	0.0473631	yes
XLOC_001950	XLOC_001950	-	jgi|Fusox2|1566|FOXG_00798T0:0-2493	FOXAMP	FOXNTC	OK	183.274	28.9842	-2.66066	-2.43972	0.00165	0.0395679	yes
XLOC_007757	XLOC_007757	-	jgi|Fusox2|6649|FOXG_20400T0:16-2138	FOXAMP	FOXNTC	OK	779.489	124.312	-2.64857	-2.47574	0.00055	0.0199502	yes
XLOC_003100	XLOC_003100	-	jgi|Fusox2|1930|FOXG_00995T0:77-2219	FOXAMP	FOXNTC	OK	124.241	19.9622	-2.63779	-2.8056	0.0003	0.0128851	yes
XLOC_006147	XLOC_006147	-	jgi|Fusox2|3004|FOXG_08418T0:0-2430	FOXAMP	FOXNTC	OK	246.54	39.6549	-2.63625	-2.81941	0.0001	0.00560584	yes
XLOC_005707	XLOC_005707	-	jgi|Fusox2|26278|FOXG_07867T0:45-2202	FOXAMP	FOXNTC	OK	17.3757	2.80021	-2.63346	-2.57893	0.0017	0.0398807	yes
XLOC_003667	XLOC_003667	-	jgi|Fusox2|20510|FOXG_04328T1:46-1553	FOXAMP	FOXNTC	OK	61.941	9.98459	-2.63312	-2.84094	0.00015	0.00757281	yes
XLOC_001008	XLOC_001008	-	jgi|Fusox2|12489|FOXG_02370T0:3-3987	FOXAMP	FOXNTC	OK	244.077	39.3883	-2.6315	-2.32074	0.002	0.0443856	yes
XLOC_007118	XLOC_007118	-	jgi|Fusox2|5091|FOXG_09835T0:51-1850	FOXAMP	FOXNTC	OK	52.036	8.4083	-2.62962	-2.59355	0.001	0.0294642	yes
XLOC_006982	XLOC_006982	-	jgi|Fusox2|4875|FOXG_09666T0:132-1181	FOXAMP	FOXNTC	OK	49.3796	8.04524	-2.61771	-2.52664	0.0022	0.0473631	yes
XLOC_007604	XLOC_007604	-	jgi|Fusox2|6256|FOXG_10601T0:2-879	FOXAMP	FOXNTC	OK	56.6767	9.30581	-2.60655	-2.56809	0.00225	0.0481991	yes
XLOC_003740	XLOC_003740	-	jgi|Fusox2|2064|FOXG_01070T0:3-2711	FOXAMP	FOXNTC	OK	6.95737	41.9634	2.59252	2.63193	0.00065	0.0223564	yes
XLOC_005178	XLOC_005178	-	jgi|Fusox2|24542|FOXG_06318T0:4-3000	FOXAMP	FOXNTC	OK	72.1042	11.9583	-2.59208	-2.76451	0.00025	0.0112409	yes
XLOC_004575	XLOC_004575	-	jgi|Fusox2|22927|FOXG_05468T0:65-1104	FOXAMP	FOXNTC	OK	101.974	613.594	2.58909	2.55738	0.0007	0.023514	yes
XLOC_002359	XLOC_002359	-	jgi|Fusox2|16828|FOXG_03024T0:0-2112	FOXAMP	FOXNTC	OK	458.682	76.2669	-2.58837	-2.31528	0.0017	0.0398807	yes
XLOC_000188	XLOC_000188	-	jgi|Fusox2|10404|FOXG_13351T0:78-2099	FOXAMP	FOXNTC	OK	6.81723	1.13416	-2.58756	-2.28407	0.002	0.0443856	yes
XLOC_002590	XLOC_002590	-	jgi|Fusox2|17376|FOXG_18528T0:1-3606	FOXAMP	FOXNTC	OK	51.9502	8.64423	-2.58732	-2.57723	0.0018	0.0411095	yes
XLOC_000329	XLOC_000329	-	jgi|Fusox2|10787|FOXG_01465T0:2-3215	FOXAMP	FOXNTC	OK	133.584	22.3511	-2.57933	-2.48871	0.00155	0.0380146	yes
XLOC_008869	XLOC_008869	-	jgi|Fusox2|900|FOXG_00452T0:76-1876	FOXAMP	FOXNTC	OK	1030.96	172.563	-2.5788	-2.2632	0.0018	0.0411095	yes
XLOC_003353	XLOC_003353	-	jgi|Fusox2|19824|FOXG_03887T0:30-2306	FOXAMP	FOXNTC	OK	90.522	15.28	-2.56662	-2.87286	5e-05	0.00322127	yes
XLOC_000027	XLOC_000027	-	jgi|Fusox2|10079|FOXG_13106T0:0-1791	FOXAMP	FOXNTC	OK	52.0993	8.81336	-2.5635	-2.53175	0.00135	0.0344809	yes
XLOC_004977	XLOC_004977	-	jgi|Fusox2|24077|FOXG_06034T0:4-1340	FOXAMP	FOXNTC	OK	73.6361	12.5006	-2.55841	-2.77714	0.0006	0.0215825	yes
XLOC_004932	XLOC_004932	-	jgi|Fusox2|23994|FOXG_05975T0:9-2676	FOXAMP	FOXNTC	OK	196.964	33.4573	-2.55754	-2.58543	0.0005	0.0184466	yes
XLOC_004958	XLOC_004958	-	jgi|Fusox2|24040|FOXG_06011T0:8-263	FOXAMP	FOXNTC	OK	104.299	17.8337	-2.54805	-2.40105	0.00215	0.0465187	yes
XLOC_000236	XLOC_000236	-	jgi|Fusox2|10516|FOXG_13439T0:122-1254	FOXAMP	FOXNTC	OK	51.8443	8.90607	-2.54132	-2.52946	0.00135	0.0344809	yes
XLOC_005095	XLOC_005095	-	jgi|Fusox2|24346|FOXG_06199T0:0-1161	FOXAMP	FOXNTC	OK	73.3127	12.5941	-2.54131	-2.49938	0.00195	0.0436123	yes

XLOC_001843	XLOC_001843	-	jgi|Fusox2|15365|FOXG_15366T0:0-637	FOXAMP	FOXNTC	OK	10.618	61.6811	2.53832	2.47364	0.0018	0.0411095	yes
XLOC_008984	XLOC_008984	-	jgi|Fusox2|925|FOXG_00469T0:53-1298	FOXAMP	FOXNTC	OK	24.9239	4.29366	-2.53725	-2.44811	0.0023	0.0490269	yes
XLOC_008672	XLOC_008672	-	jgi|Fusox2|8679|FOXG_12296T0:0-2412	FOXAMP	FOXNTC	OK	35.2166	6.07195	-2.53602	-2.43671	0.00125	0.0336176	yes
XLOC_001980	XLOC_001980	-	jgi|Fusox2|1577|FOXG_17953T0:0-928	FOXAMP	FOXNTC	OK	51.2615	8.85038	-2.53407	-2.46732	0.00155	0.0380146	yes
XLOC_005533	XLOC_005533	-	jgi|Fusox2|25964|FOXG_07663T0:55-1707	FOXAMP	FOXNTC	OK	58.2879	10.0969	-2.52928	-2.53184	0.00175	0.0405034	yes
XLOC_006991	XLOC_006991	-	jgi|Fusox2|4886|FOXG_09673T0:19-882	FOXAMP	FOXNTC	OK	63.5886	11.0855	-2.52009	-2.48657	0.0015	0.0374263	yes
XLOC_008929	XLOC_008929	-	jgi|Fusox2|9133|FOXG_12823T0:1-3444	FOXAMP	FOXNTC	OK	69.1552	12.1114	-2.51347	-2.82207	0.0002	0.00959222	yes
XLOC_008192	XLOC_008192	-	jgi|Fusox2|762|FOXG_00375T0:18-1748	FOXAMP	FOXNTC	OK	77.2153	13.528	-2.51294	-2.489	0.0014	0.0355476	yes
XLOC_001783	XLOC_001783	-	jgi|Fusox2|15231|FOXG_15266T0:3128-4203	FOXAMP	FOXNTC	OK	13.6215	77.598	2.51013	2.4029	0.0012	0.0329924	yes
XLOC_005705	XLOC_005705	-	jgi|Fusox2|26276|FOXG_07866T0:1-2505	FOXAMP	FOXNTC	OK	93.8349	16.66	-2.49374	-2.62534	0.0005	0.0184466	yes
XLOC_002212	XLOC_002212	-	jgi|Fusox2|16511|FOXG_02799T0:29-1265	FOXAMP	FOXNTC	OK	55.4788	9.85254	-2.49337	-2.62288	0.00035	0.0143883	yes
XLOC_002033	XLOC_002033	-	jgi|Fusox2|16081|FOXG_22108T0:2321-3583	FOXAMP	FOXNTC	OK	10.1217	56.8588	2.48993	2.20477	0.00075	0.0248075	yes
XLOC_006227	XLOC_006227	-	jgi|Fusox2|3186|FOXG_08526T0:38-4091	FOXAMP	FOXNTC	OK	267.234	47.6788	-2.48668	-2.49875	0.00145	0.0363891	yes
XLOC_007265	XLOC_007265	-	jgi|Fusox2|5407|FOXG_10089T0:31-1726	FOXAMP	FOXNTC	OK	166.691	30.1736	-2.46581	-2.31525	0.00205	0.0451471	yes
XLOC_008763	XLOC_008763	-	jgi|Fusox2|8829|FOXG_12649T0:4-1718	FOXAMP	FOXNTC	OK	375.94	68.2762	-2.46105	-2.62768	0.0004	0.0156964	yes
XLOC_004279	XLOC_004279	-	jgi|Fusox2|2225|FOXG_01162T0:25-872	FOXAMP	FOXNTC	OK	95.2341	17.3836	-2.45375	-2.61186	0.00055	0.0199502	yes
XLOC_002505	XLOC_002505	-	jgi|Fusox2|17182|FOXG_03200T0:30-3129	FOXAMP	FOXNTC	OK	11.0447	60.4072	2.45137	2.76064	0.00015	0.00757281	yes
XLOC_005719	XLOC_005719	-	jgi|Fusox2|2629|FOXG_08211T0:3-1532	FOXAMP	FOXNTC	OK	6.21659	33.9376	2.44869	2.40771	0.00195	0.0436123	yes
XLOC_000533	XLOC_000533	-	jgi|Fusox2|11267|FOXG_01739T0:20-3466	FOXAMP	FOXNTC	OK	11.5851	63.2361	2.44848	2.65552	0.0003	0.0128851	yes
XLOC_006474	XLOC_006474	-	jgi|Fusox2|3770|FOXG_08937T0:165-1796	FOXAMP	FOXNTC	OK	54.835	10.0481	-2.44818	-2.33174	0.0008	0.0258667	yes
XLOC_003118	XLOC_003118	-	jgi|Fusox2|19367|FOXG_22596T0:6-505	FOXAMP	FOXNTC	OK	228.535	41.9486	-2.44572	-2.26056	0.00115	0.0321293	yes
XLOC_003681	XLOC_003681	-	jgi|Fusox2|20532|FOXG_04345T0:1-489	FOXAMP	FOXNTC	OK	575.955	106.016	-2.44167	-2.28568	0.0018	0.0411095	yes
XLOC_006506	XLOC_006506	-	jgi|Fusox2|3821|FOXG_08982T0:154-3983	FOXAMP	FOXNTC	OK	279.204	51.4077	-2.44126	-2.44199	0.00155	0.0380146	yes
XLOC_000872	XLOC_000872	-	jgi|Fusox2|12187|FOXG_02207T0:3-858	FOXAMP	FOXNTC	OK	77.6211	14.4382	-2.42656	-2.21794	0.00135	0.0344809	yes
XLOC_005704	XLOC_005704	-	jgi|Fusox2|26275|FOXG_07865T0:0-829	FOXAMP	FOXNTC	OK	87.3365	16.2989	-2.42181	-2.54413	0.0005	0.0184466	yes
XLOC_006419	XLOC_006419	-	jgi|Fusox2|361|FOXG_00184T0:8-2495	FOXAMP	FOXNTC	OK	90.522	16.9682	-2.41544	-2.55283	0.00025	0.0112409	yes
XLOC_006553	XLOC_006553	-	jgi|Fusox2|3908|FOXG_09044T0:0-1726	FOXAMP	FOXNTC	OK	231.057	43.5421	-2.40777	-2.60612	0.0008	0.0258667	yes
XLOC_000203	XLOC_000203	-	jgi|Fusox2|10432|FOXG_13371T0:46-2332	FOXAMP	FOXNTC	OK	11.0222	58.1622	2.39967	2.66497	0.00025	0.0112409	yes
XLOC_005720	XLOC_005720	-	jgi|Fusox2|262|FOXG_00133T0:0-1755	FOXAMP	FOXNTC	OK	244.023	46.2677	-2.39894	-2.43874	0.00085	0.0266838	yes
XLOC_007807	XLOC_007807	-	jgi|Fusox2|6781|FOXG_11000T0:438-1303	FOXAMP	FOXNTC	OK	165.718	31.481	-2.39618	-2.30679	0.001	0.0294642	yes
XLOC_001269	XLOC_001269	-	jgi|Fusox2|13100|FOXG_13742T0:96-2079	FOXAMP	FOXNTC	OK	247.17	47.1785	-2.3893	-2.4317	0.00135	0.0344809	yes
XLOC_005611	XLOC_005611	-	jgi|Fusox2|26078|FOXG_07752T0:0-3007	FOXAMP	FOXNTC	OK	145.362	27.8316	-2.38485	-2.4597	0.00035	0.0143883	yes
XLOC_006219	XLOC_006219	-	jgi|Fusox2|3176|FOXG_08520T0:0-3125	FOXAMP	FOXNTC	OK	38.982	7.57871	-2.36278	-2.556	0.00105	0.0302155	yes
XLOC_006060	XLOC_006060	-	jgi|Fusox2|2797|FOXG_08301T0:4-2632	FOXAMP	FOXNTC	OK	3.76153	19.1166	2.34544	2.1276	0.00155	0.0380146	yes
XLOC_004629	XLOC_004629	-	jgi|Fusox2|23093|FOXG_05544T0:170-2171	FOXAMP	FOXNTC	OK	32.4248	6.38804	-2.34365	-2.44688	0.00075	0.0248075	yes
XLOC_004154	XLOC_004154	-	jgi|Fusox2|21969|FOXG_04918T0:74-4326	FOXAMP	FOXNTC	OK	55.1033	10.9064	-2.33697	-2.4518	0.001	0.0294642	yes
XLOC_000995	XLOC_000995	-	jgi|Fusox2|12467|FOXG_02354T0:13-1449	FOXAMP	FOXNTC	OK	53.8285	10.6766	-2.33392	-2.49478	0.001	0.0294642	yes
XLOC_006995	XLOC_006995	-	jgi|Fusox2|4890|FOXG_09677T0:1-5442	FOXAMP	FOXNTC	OK	25.9438	5.14775	-2.33337	-2.60386	0.00025	0.0112409	yes
XLOC_006252	XLOC_006252	-	jgi|Fusox2|3244|FOXG_08559T0:6-3708	FOXAMP	FOXNTC	OK	80.5674	16.0274	-2.32966	-2.50334	0.00105	0.0302155	yes
XLOC_008337	XLOC_008337	-	jgi|Fusox2|7924|FOXG_11724T0:3-1211	FOXAMP	FOXNTC	OK	110.839	22.1478	-2.32323	-2.47964	0.00105	0.0302155	yes
XLOC_001029	XLOC_001029	-	jgi|Fusox2|12539|FOXG_02398T0:46-1841	FOXAMP	FOXNTC	OK	38.245	7.65414	-2.32096	-2.44328	0.00095	0.028676	yes
XLOC_003688	XLOC_003688	-	jgi|Fusox2|20544|FOXG_18810T0:0-1583	FOXAMP	FOXNTC	OK	11.3268	56.569	2.32027	2.12671	0.0019	0.0427154	yes
XLOC_007320	XLOC_007320	-	jgi|Fusox2|5624|FOXG_10255T0:0-2493	FOXAMP	FOXNTC	OK	172.399	34.6396	-2.31526	-2.48513	0.0008	0.0258667	yes
XLOC_003630	XLOC_003630	-	jgi|Fusox2|20433|FOXG_04284T0:0-1722	FOXAMP	FOXNTC	OK	211.623	42.5413	-2.31456	-2.50215	0.0011	0.0310337	yes
XLOC_003834	XLOC_003834	-	jgi|Fusox2|2086|FOXG_01086T0:7-1228	FOXAMP	FOXNTC	OK	50.0101	10.0886	-2.30949	-2.43868	0.00095	0.028676	yes
XLOC_007834	XLOC_007834	-	jgi|Fusox2|6846|FOXG_11036T0:1-2376	FOXAMP	FOXNTC	OK	127.788	25.8609	-2.30491	-2.44352	0.0013	0.0343208	yes
XLOC_006617	XLOC_006617	-	jgi|Fusox2|4007|FOXG_09121T0:0-2390	FOXAMP	FOXNTC	OK	27.7639	5.6423	-2.29886	-2.45247	0.00155	0.0380146	yes
XLOC_005730	XLOC_005730	-	jgi|Fusox2|26311|FOXG_07890T0:98-2374	FOXAMP	FOXNTC	OK	35.1439	7.16709	-2.29382	-2.4756	0.00095	0.028676	yes
XLOC_008468	XLOC_008468	-	jgi|Fusox2|8245|FOXG_11978T0:57-1136	FOXAMP	FOXNTC	OK	13.3323	65.2562	2.29119	2.45698	0.0008	0.0258667	yes
XLOC_003486	XLOC_003486	-	jgi|Fusox2|20104|FOXG_04094T0:1-3499	FOXAMP	FOXNTC	OK	113.01	23.1018	-2.29037	-2.43769	0.0011	0.0310337	yes
XLOC_006508	XLOC_006508	-	jgi|Fusox2|3825|FOXG_08985T0:73-1274	FOXAMP	FOXNTC	OK	90.0904	18.6212	-2.27443	-2.41986	0.00105	0.0302155	yes
XLOC_008623	XLOC_008623	-	jgi|Fusox2|8549|FOXG_20691T0:61-1406	FOXAMP	FOXNTC	OK	103.309	21.4535	-2.26768	-2.36304	0.0007	0.023514	yes
XLOC_002098	XLOC_002098	-	jgi|Fusox2|16214|FOXG_15760T0:1-1797	FOXAMP	FOXNTC	OK	124.877	26.0041	-2.26369	-2.43119	0.0014	0.0355476	yes
XLOC_007276	XLOC_007276	-	jgi|Fusox2|543|FOXG_00256T0:9-2241	FOXAMP	FOXNTC	OK	263.643	54.931	-2.26289	-2.3277	0.0012	0.0329924	yes
XLOC_009032	XLOC_009032	-	jgi|Fusox2|934|FOXG_00475T0:7-2204	FOXAMP	FOXNTC	OK	30.6907	6.40323	-2.26093	-2.38828	0.00105	0.0302155	yes
XLOC_007122	XLOC_007122	-	jgi|Fusox2|5100|FOXG_09842T0:15-2846	FOXAMP	FOXNTC	OK	157.376	32.8853	-2.2587	-2.46866	0.0012	0.0329924	yes
XLOC_002038	XLOC_002038	-	jgi|Fusox2|16097|FOXG_15675T0:1149-3537	FOXAMP	FOXNTC	OK	38.979	8.14785	-2.25821	-2.42219	0.0016	0.0388	yes
XLOC_002046	XLOC_002046	-	jgi|Fusox2|16121|FOXG_15694T0:4-793	FOXAMP	FOXNTC	OK	304.874	63.793	-2.25674	-2.55015	0.00045	0.0171896	yes
XLOC_001371	XLOC_001371	-	jgi|Fusox2|13308|FOXG_13870T0:4-2125	FOXAMP	FOXNTC	OK	39.1965	8.28527	-2.2421	-2.39045	0.00105	0.0302155	yes
XLOC_005732	XLOC_005732	-	jgi|Fusox2|26313|FOXG_07892T0:0-1115	FOXAMP	FOXNTC	OK	51.5286	243.428	2.24005	2.40752	0.001	0.0294642	yes
XLOC_005753	XLOC_005753	-	jgi|Fusox2|26344|FOXG_07913T0:15-1762	FOXAMP	FOXNTC	OK	46.2031	9.91519	-2.22028	-2.38112	0.00185	0.0420291	yes
XLOC_005197	XLOC_005197	-	jgi|Fusox2|24584|FOXG_06342T0:24-1511	FOXAMP	FOXNTC	OK	97.8325	21.1369	-2.21055	-2.3589	0.00135	0.0344809	yes
XLOC_007809	XLOC_007809	-	jgi|Fusox2|6788|FOXG_11004T0:51-2275	FOXAMP	FOXNTC	OK	248.837	54.246	-2.19761	-2.32361	0.00125	0.0336176	yes
XLOC_005191	XLOC_005191	-	jgi|Fusox2|24578|FOXG_06336T0:20-4175	FOXAMP	FOXNTC	OK	20.1002	4.43033	-2.18172	-2.33518	0.00205	0.0451471	yes
XLOC_001837	XLOC_001837	-	jgi|Fusox2|15351|FOXG_15359T0:76-978	FOXAMP	FOXNTC	OK	68.154	15.1388	-2.17055	-2.21478	0.00175	0.0405034	yes
XLOC_004327	XLOC_004327	-	jgi|Fusox2|22355|FOXG_05159T0:0-2495	FOXAMP	FOXNTC	OK	266.422	59.2558	-2.16869	-2.24715	0.00085	0.0266838	yes
XLOC_004766	XLOC_004766	-	jgi|Fusox2|23594|FOXG_05704T0:24-1813	FOXAMP	FOXNTC	OK	48.3359	10.8543	-2.15483	-2.3065	0.0023	0.0490269	yes
XLOC_003014	XLOC_003014	-	jgi|Fusox2|18947|FOXG_16551T0:43-1716	FOXAMP	FOXNTC	OK	36.4033	8.55945	-2.08848	-2.16508	0.00235	0.0498466	yes
XLOC_008236	XLOC_008236	-	jgi|Fusox2|7729|FOXG_11588T0:17-6101	FOXAMP	FOXNTC	OK	30.9862	7.31295	-2.0831	-2.23071	0.0017	0.0398807	yes
XLOC_003508	XLOC_003508	-	jgi|Fusox2|20150|FOXG_04124T0:3-938	FOXAMP	FOXNTC	OK	58.8901	14.0843	-2.06394	-2.16627	0.00235	0.0498466	yes
XLOC_002137	XLOC_002137	-	jgi|Fusox2|16300|FOXG_15833T0:24-1330	FOXAMP	FOXNTC	OK	34.6047	138.885	2.00485	2.27774	0.00175	0.0405034	yes
XLOC_008600	XLOC_008600	-	jgi|Fusox2|849|FOXG_17876T0:72-3643	FOXAMP	FOXNTC	OK	54.3547	13.5822	-2.00069	-2.27226	0.00145	0.0363891	yes
XLOC_006585	XLOC_006585	-	jgi|Fusox2|3969|FOXG_09087T0:9-5233	FOXAMP	FOXNTC	OK	28.9169	7.43202	-1.96008	-2.23282	0.00205	0.0451471	yes

F. oxysporum + PSC

test_id	gene_id	gene	locus	sample_1	sample_2	status	value_1	value_2	log2(fold_change)	test_stat	p_value	q_value	significant
XLOC_000489	XLOC_000489	-	jgi|Fusox2|11162|FOXG_01683T0:16-140	FOXPSC	FOXNTC	OK	0	27.1885	6.54621	-nan	0.00045	0.0471054	yes
XLOC_000653	XLOC_000653	-	jgi|Fusox2|11588|FOXG_18208T0:1737-2058	FOXPSC	FOXNTC	OK	0	4.75932	6.54621	-nan	0.00045	0.0471054	yes
XLOC_001024	XLOC_001024	-	jgi|Fusox2|12532|FOXG_02391T0:0-138	FOXPSC	FOXNTC	OK	0	31.3342	6.54621	-nan	0.00015	0.0299761	yes
XLOC_001174	XLOC_001174	-	jgi|Fusox2|12923|FOXG_13600T0:294-519	FOXPSC	FOXNTC	OK	0	7.95881	6.54621	-nan	0.00045	0.0471054	yes
XLOC_001660	XLOC_001660	-	jgi|Fusox2|14803|FOXG_21764T0:516-1281	FOXPSC	FOXNTC	OK	0	4.79344	6.54621	-nan	5e-05	0.017586	yes
XLOC_001737	XLOC_001737	-	jgi|Fusox2|15063|FOXG_14970T0:1-1186	FOXPSC	FOXNTC	OK	0	2.85394	6.54621	-nan	5e-05	0.017586	yes
XLOC_001738	XLOC_001738	-	jgi|Fusox2|15064|FOXG_14971T0:78-1731	FOXPSC	FOXNTC	OK	0	3.50638	6.54621	-nan	0.0001	0.0283645	yes
XLOC_001927	XLOC_001927	-	jgi|Fusox2|15542|FOXG_21930T0:459-648	FOXPSC	FOXNTC	OK	0	31.1059	6.54621	-nan	5e-05	0.017586	yes
XLOC_002043	XLOC_002043	-	jgi|Fusox2|16108|FOXG_22115T0:136-366	FOXPSC	FOXNTC	OK	0	8.28108	6.54621	-nan	0.0004	0.046896	yes
XLOC_002060	XLOC_002060	-	jgi|Fusox2|16153|FOXG_15713T0:1102-1512	FOXPSC	FOXNTC	OK	0	5.06302	6.54621	-nan	0.00025	0.0382304	yes
XLOC_002214	XLOC_002214	-	jgi|Fusox2|16515|FOXG_02802T0:61-885	FOXPSC	FOXNTC	OK	0	6.85519	6.54621	-nan	0.00015	0.0299761	yes
XLOC_002230	XLOC_002230	-	jgi|Fusox2|16548|FOXG_02830T0:1497-1853	FOXPSC	FOXNTC	OK	0	5.60891	6.54621	-nan	0.00045	0.0471054	yes
XLOC_002939	XLOC_002939	-	jgi|Fusox2|18476|FOXG_22323T0:13-322	FOXPSC	FOXNTC	OK	0	6.16755	6.54621	-nan	0.0003	0.0382304	yes
XLOC_002942	XLOC_002942	-	jgi|Fusox2|18488|FOXG_16126T0:1925-2190	FOXPSC	FOXNTC	OK	0	6.2173	6.54621	-nan	0.00045	0.0471054	yes
XLOC_003243	XLOC_003243	-	jgi|Fusox2|1959|FOXG_17995T0:0-118	FOXPSC	FOXNTC	OK	0	36.6288	6.54621	-nan	0.0003	0.0382304	yes
XLOC_003516	XLOC_003516	-	jgi|Fusox2|20159|FOXG_04132T0:531-630	FOXPSC	FOXNTC	OK	0	104.065	6.54621	-nan	0.00015	0.0299761	yes
XLOC_003542	XLOC_003542	-	jgi|Fusox2|20210|FOXG_18761T0:0-260	FOXPSC	FOXNTC	OK	0	69.6361	6.54621	-nan	5e-05	0.017586	yes
XLOC_003628	XLOC_003628	-	jgi|Fusox2|20431|FOXG_04282T0:28-363	FOXPSC	FOXNTC	OK	0	7.26236	6.54621	-nan	5e-05	0.017586	yes
XLOC_003672	XLOC_003672	-	jgi|Fusox2|20518|FOXG_04332T0:411-504	FOXPSC	FOXNTC	OK	0	121.401	6.54621	-nan	0.0003	0.0382304	yes
XLOC_003828	XLOC_003828	-	jgi|Fusox2|20845|FOXG_04571T0:222-330	FOXPSC	FOXNTC	OK	0	57.6015	6.54621	-nan	0.00035	0.0433458	yes
XLOC_003853	XLOC_003853	-	jgi|Fusox2|20946|FOXG_04660T0:452-1043	FOXPSC	FOXNTC	OK	0	2.02974	6.54621	-nan	0.0003	0.0382304	yes
XLOC_003908	XLOC_003908	-	jgi|Fusox2|21113|FOXG_17033T0:45-1183	FOXPSC	FOXNTC	OK	0	2.07013	6.54621	-nan	0.00015	0.0299761	yes
XLOC_004006	XLOC_004006	-	jgi|Fusox2|21638|FOXG_17422T0:12564-13335	FOXPSC	FOXNTC	OK	0	2.41156	6.54621	-nan	0.0003	0.0382304	yes
XLOC_004030	XLOC_004030	-	jgi|Fusox2|21687|FOXG_04704T0:5-1626	FOXPSC	FOXNTC	OK	0	1.89134	6.54621	-nan	5e-05	0.017586	yes
XLOC_004077	XLOC_004077	-	jgi|Fusox2|21820|FOXG_04811T0:1419-1873	FOXPSC	FOXNTC	OK	0	8.51665	6.54621	-nan	0.0003	0.0382304	yes
XLOC_004715	XLOC_004715	-	jgi|Fusox2|23493|FOXG_05619T0:372-437	FOXPSC	FOXNTC	OK	0	26051.4	6.54621	-nan	0.00015	0.0299761	yes
XLOC_004779	XLOC_004779	-	jgi|Fusox2|23636|FOXG_05724T0:879-1031	FOXPSC	FOXNTC	OK	0	16.2823	6.54621	-nan	0.00045	0.0471054	yes
XLOC_004780	XLOC_004780	-	jgi|Fusox2|23636|FOXG_05724T0:1084-1389	FOXPSC	FOXNTC	OK	0	10.5946	6.54621	-nan	5e-05	0.017586	yes
XLOC_004805	XLOC_004805	-	jgi|Fusox2|2368|FOXG_01259T0:2726-2958	FOXPSC	FOXNTC	OK	0	29.1804	6.54621	-nan	5e-05	0.017586	yes
XLOC_005325	XLOC_005325	-	jgi|Fusox2|25532|FOXG_19467T0:585-665	FOXPSC	FOXNTC	OK	0	887.805	6.54621	-nan	0.0004	0.046896	yes
XLOC_006064	XLOC_006064	-	jgi|Fusox2|2817|FOXG_19789T0:11-186	FOXPSC	FOXNTC	OK	0	23.5517	6.54621	-nan	0.00015	0.0299761	yes
XLOC_007089	XLOC_007089	-	jgi|Fusox2|5051|FOXG_09802T0:144-1025	FOXPSC	FOXNTC	OK	0	1.97003	6.54621	-nan	0.00045	0.0471054	yes
XLOC_007248	XLOC_007248	-	jgi|Fusox2|5369|FOXG_10053T0:15-1698	FOXPSC	FOXNTC	OK	0	4.20526	6.54621	-nan	5e-05	0.017586	yes
XLOC_007364	XLOC_007364	-	jgi|Fusox2|5720|FOXG_20270T0:2-256	FOXPSC	FOXNTC	OK	0	11.6534	6.54621	-nan	0.0003	0.0382304	yes
XLOC_007736	XLOC_007736	-	jgi|Fusox2|6620|FOXG_10868T0:189-449	FOXPSC	FOXNTC	OK	0	7.37554	6.54621	-nan	0.0003	0.0382304	yes
XLOC_007737	XLOC_007737	-	jgi|Fusox2|6620|FOXG_10868T0:561-722	FOXPSC	FOXNTC	OK	0	17.751	6.54621	-nan	0.0003	0.0382304	yes
XLOC_007800	XLOC_007800	-	jgi|Fusox2|6769|FOXG_10992T0:76-151	FOXPSC	FOXNTC	OK	0	16994.6	6.54621	-nan	5e-05	0.017586	yes
XLOC_008358	XLOC_008358	-	jgi|Fusox2|8001|FOXG_11774T0:1441-2015	FOXPSC	FOXNTC	OK	0	2.84426	6.54621	-nan	0.0003	0.0382304	yes
XLOC_008546	XLOC_008546	-	jgi|Fusox2|8390|FOXG_12096T0:614-872	FOXPSC	FOXNTC	OK	0	11.4378	6.54621	-nan	0.00015	0.0299761	yes
XLOC_001395	XLOC_001395	-	jgi|Fusox2|13380|FOXG_21291T0:77-2101	FOXPSC	FOXNTC	OK	0.806206	150.689	7.54621	4.53274	5e-05	0.017586	yes
XLOC_002321	XLOC_002321	-	jgi|Fusox2|16745|FOXG_18445T0:5960-6580	FOXPSC	FOXNTC	OK	6.75484	533.413	6.30319	3.61602	0.0002	0.035172	yes
XLOC_001998	XLOC_001998	-	jgi|Fusox2|1596|FOXG_17956T0:0-1900	FOXPSC	FOXNTC	OK	8.13819	566.934	6.12233	3.54087	5e-05	0.017586	yes
XLOC_007139	XLOC_007139	-	jgi|Fusox2|512|FOXG_00241T0:1-3141	FOXPSC	FOXNTC	OK	0.583283	35.3623	5.92187	4.09081	5e-05	0.017586	yes
XLOC_001999	XLOC_001999	-	jgi|Fusox2|1597|FOXG_17957T0:53-2481	FOXPSC	FOXNTC	OK	4.79646	220.97	5.52573	3.43866	0.0001	0.0283645	yes
XLOC_006388	XLOC_006388	-	jgi|Fusox2|3538|FOXG_08765T0:120-1569	FOXPSC	FOXNTC	OK	2137.13	46.7081	-5.51586	-3.33805	5e-05	0.017586	yes
XLOC_002502	XLOC_002502	-	jgi|Fusox2|17179|FOXG_03197T0:0-1305	FOXPSC	FOXNTC	OK	12.7722	554.127	5.43913	3.27179	0.00015	0.0299761	yes
XLOC_000577	XLOC_000577	-	jgi|Fusox2|11369|FOXG_01792T0:12-1150	FOXPSC	FOXNTC	OK	435.736	11.3509	-5.26257	-3.84642	5e-05	0.017586	yes
XLOC_003130	XLOC_003130	-	jgi|Fusox2|19392|FOXG_16822T0:28-1544	FOXPSC	FOXNTC	OK	252.366	6.69001	-5.23737	-3.72357	5e-05	0.017586	yes
XLOC_001871	XLOC_001871	-	jgi|Fusox2|15425|FOXG_15413T0:0-715	FOXPSC	FOXNTC	OK	474.572	12.9258	-5.1983	-3.80186	5e-05	0.017586	yes
XLOC_004519	XLOC_004519	-	jgi|Fusox2|22843|FOXG_19057T0:33-4163	FOXPSC	FOXNTC	OK	4.87036	166.293	5.09356	3.18868	0.00035	0.0433458	yes
XLOC_004727	XLOC_004727	-	jgi|Fusox2|23526|FOXG_05648T0:0-976	FOXPSC	FOXNTC	OK	388.888	12.1538	-4.99988	-3.66883	5e-05	0.017586	yes
XLOC_000079	XLOC_000079	-	jgi|Fusox2|10184|FOXG_13185T0:50-2282	FOXPSC	FOXNTC	OK	392.005	12.7197	-4.94574	-3.61008	0.0001	0.0283645	yes
XLOC_005344	XLOC_005344	-	jgi|Fusox2|25642|FOXG_07399T0:78-1457	FOXPSC	FOXNTC	OK	34.5549	1.1214	-4.94552	-3.71222	0.0001	0.0283645	yes
XLOC_005038	XLOC_005038	-	jgi|Fusox2|24231|FOXG_19186T0:3-528	FOXPSC	FOXNTC	OK	1.99415	56.7925	4.83185	3.14674	0.00025	0.0382304	yes
XLOC_003233	XLOC_003233	-	jgi|Fusox2|1958|FOXG_01015T0:1316-2621	FOXPSC	FOXNTC	OK	21.6479	615.876	4.83034	2.86326	5e-05	0.017586	yes
XLOC_001619	XLOC_001619	-	jgi|Fusox2|14427|FOXG_14750T0:6-1267	FOXPSC	FOXNTC	OK	23.3793	1.05251	-4.47332	-3.08955	0.0003	0.0382304	yes
XLOC_008011	XLOC_008011	-	jgi|Fusox2|7183|FOXG_11254T0:0-1618	FOXPSC	FOXNTC	OK	85.1553	3.84845	-4.46775	-3.58148	0.0001	0.0283645	yes
XLOC_009060	XLOC_009060	-	jgi|Fusox2|9453|FOXG_13040T0:6-728	FOXPSC	FOXNTC	OK	2.85618	61.2784	4.42322	3.32411	0.00025	0.0382304	yes
XLOC_006932	XLOC_006932	-	jgi|Fusox2|4751|FOXG_09572T0:0-1161	FOXPSC	FOXNTC	OK	435.762	20.4042	-4.4166	-4.00445	5e-05	0.017586	yes
XLOC_007433	XLOC_007433	-	jgi|Fusox2|5853|FOXG_10386T0:11-1889	FOXPSC	FOXNTC	OK	100.542	4.74093	-4.40649	-3.5028	0.00015	0.0299761	yes
XLOC_005496	XLOC_005496	-	jgi|Fusox2|25912|FOXG_07622T0:73-2691	FOXPSC	FOXNTC	OK	169.705	8.04764	-4.39832	-3.5067	5e-05	0.017586	yes
XLOC_002814	XLOC_002814	-	jgi|Fusox2|17869|FOXG_03599T0:0-582	FOXPSC	FOXNTC	OK	4.22428	86.9858	4.364	3.02968	0.0002	0.035172	yes
XLOC_000921	XLOC_000921	-	jgi|Fusox2|12341|FOXG_02285T0:2-1567	FOXPSC	FOXNTC	OK	515.962	25.2473	-4.35307	-3.42464	5e-05	0.017586	yes
XLOC_002998	XLOC_002998	-	jgi|Fusox2|18911|FOXG_16528T0:92-1740	FOXPSC	FOXNTC	OK	200.744	9.90991	-4.34034	-3.43153	0.0001	0.0283645	yes
XLOC_005275	XLOC_005275	-	jgi|Fusox2|2476|FOXG_01332T0:478-2365	FOXPSC	FOXNTC	OK	3.97144	79.8634	4.3298	2.97199	5e-05	0.017586	yes
XLOC_006592	XLOC_006592	-	jgi|Fusox2|3976|FOXG_09094T0:4-2027	FOXPSC	FOXNTC	OK	88.1305	4.4117	-4.32023	-3.28087	0.0002	0.035172	yes
XLOC_005482	XLOC_005482	-	jgi|Fusox2|25896|FOXG_19513T0:0-541	FOXPSC	FOXNTC	OK	3.78506	72.8217	4.26598	2.88711	0.00015	0.0299761	yes
XLOC_004777	XLOC_004777	-	jgi|Fusox2|23622|FOXG_05720T0:35-1334	FOXPSC	FOXNTC	OK	2.47593	47.1636	4.25163	3.09997	0.00015	0.0299761	yes
XLOC_002033	XLOC_002033	-	jgi|Fusox2|16081|FOXG_22108T0:2321-3583	FOXPSC	FOXNTC	OK	4.09501	76.6606	4.22655	3.09655	0.0002	0.035172	yes
XLOC_000640	XLOC_000640	-	jgi|Fusox2|11530|FOXG_18205T0:1-921	FOXPSC	FOXNTC	OK	3.09569	56.8559	4.19898	2.91089	0.0002	0.035172	yes
XLOC_005707	XLOC_005707	-	jgi|Fusox2|26278|FOXG_07867T0:45-2202	FOXPSC	FOXNTC	OK	66.0093	3.7611	-4.13344	-3.05759	0.00045	0.0471054	yes
XLOC_001115	XLOC_001115	-	jgi|Fusox2|12728|FOXG_02535T0:370-1546	FOXPSC	FOXNTC	OK	10.9863	190.552	4.11642	2.98866	0.00015	0.0299761	yes
XLOC_007678	XLOC_007678	-	jgi|Fusox2|6444|FOXG_10728T0:9-1119	FOXPSC	FOXNTC	OK	2.55025	43.2317	4.08338	3.0055	0.00025	0.0382304	yes
XLOC_003703	XLOC_003703	-	jgi|Fusox2|20573|FOXG_04372T0:15-1167	FOXPSC	FOXNTC	OK	6.63755	106.584	4.0052	3.29877	5e-05	0.017586	yes
XLOC_004422	XLOC_004422	-	jgi|Fusox2|22572|FOXG_05272T1:0-1807	FOXPSC	FOXNTC	OK	1.66181	25.0729	3.9153	2.80472	0.0004	0.046896	yes
XLOC_002589	XLOC_002589	-	jgi|Fusox2|17371|FOXG_03303T0:1197-3437	FOXPSC	FOXNTC	OK	1.93072	28.5478	3.88617	2.81952	0.00025	0.0382304	yes
XLOC_003687	XLOC_003687	-	jgi|Fusox2|20543|FOXG_04352T0:37-1510	FOXPSC	FOXNTC	OK	93.3261	6.4331	-3.8587	-2.84421	0.0004	0.046896	yes
XLOC_007750	XLOC_007750	-	jgi|Fusox2|6641|FOXG_10885T0:12-1214	FOXPSC	FOXNTC	OK	2.82891	40.6121	3.84359	2.70098	0.0003	0.0382304	yes
XLOC_007426	XLOC_007426	-	jgi|Fusox2|5843|FOXG_10378T0:17-924	FOXPSC	FOXNTC	OK	3.14832	42.9143	3.7688	2.59959	0.0002	0.035172	yes
XLOC_007912	XLOC_007912	-	jgi|Fusox2|6968|FOXG_11118T1:72-1290	FOXPSC	FOXNTC	OK	46.4262	4.12726	-3.49168	-2.88112	0.00045	0.0471054	yes
XLOC_002769	XLOC_002769	-	jgi|Fusox2|17789|FOXG_03541T0:66-2397	FOXPSC	FOXNTC	OK	27.66	2.52789	-3.4518	-2.82544	0.00025	0.0382304	yes
XLOC_005464	XLOC_005464	-	jgi|Fusox2|25865|FOXG_07591T0:1-630	FOXPSC	FOXNTC	OK	579.865	56.4031	-3.36187	-3.03234	0.00015	0.0299761	yes
XLOC_005520	XLOC_005520	-	jgi|Fusox2|25948|FOXG_07650T0:0-1410	FOXPSC	FOXNTC	OK	119.144	11.8879	-3.32515	-3.06531	5e-05	0.017586	yes
XLOC_003330	XLOC_003330	-	jgi|Fusox2|19779|FOXG_03848T0:14-1486	FOXPSC	FOXNTC	OK	12.9332	94.1386	2.86371	2.48213	0.00025	0.0382304	yes

***N. solani* + AMB**

test_id	gene_id	gene	locus	sample_1	sample_2	status	value_1	value_2	log2(fold_change)	test_stat	p_value	q_value	significant
XLOC_003356	XLOC_003356	-	jgi|Necha2|42095|e_gw1.33.309.1:501-617	FSOAMP	FSONTC	OK	403.445	0	-4.61346	-nan	5e-05	0.00945943	yes
XLOC_005174	XLOC_005174	-	jgi|Necha2|63227|estExt_Genewise1.C_sca_11_chr8_3_00620:821-1147	FSOAMP	FSONTC	OK	8.51251	0	-4.61346	-nan	5e-05	0.00945943	yes
XLOC_005369	XLOC_005369	-	jgi|Necha2|64874|estExt_Genewise1.C_sca_28_chr13_5_00203:312-449	FSOAMP	FSONTC	OK	104.048	0	-4.61346	-nan	5e-05	0.00945943	yes
XLOC_006614	XLOC_006614	-	jgi|Necha2|72144|estExt_Genewise1Plus.C_sca_82_chr10_2_00004:231-377	FSOAMP	FSONTC	OK	113.996	0	-4.61346	-nan	5e-05	0.00945943	yes
XLOC_006723	XLOC_006723	-	jgi|Necha2|73052|estExt_Genewise1Plus.C_sca_15_chr12_5_00497:715-815	FSOAMP	FSONTC	OK	1051.96	0	-4.61346	-nan	5e-05	0.00945943	yes
XLOC_006732	XLOC_006732	-	jgi|Necha2|73184|estExt_Genewise1Plus.C_sca_28_chr13_5_00376:47-152	FSOAMP	FSONTC	OK	562.633	0	-4.61346	-nan	5e-05	0.00945943	yes
XLOC_007234	XLOC_007234	-	jgi|Necha2|75850|fgenesh1_pg.sca_18_chr2_1_0000041:1269-1542	FSOAMP	FSONTC	OK	11.0869	0	-4.61346	-nan	5e-05	0.00945943	yes
XLOC_007373	XLOC_007373	-	jgi|Necha2|77065|fgenesh1_pg.sca_26_chr2_2_0000141:1323-1433	FSOAMP	FSONTC	OK	446.082	0	-4.61346	-nan	5e-05	0.00945943	yes
XLOC_007488	XLOC_007488	-	jgi|Necha2|78598|fgenesh1_pg.sca_59_chr4_1_0000047:177-449	FSOAMP	FSONTC	OK	69.2765	0	-4.61346	-nan	5e-05	0.00945943	yes
XLOC_007783	XLOC_007783	-	jgi|Necha2|80618|fgenesh1_pg.sca_5_chr5_3_0000417:519-755	FSOAMP	FSONTC	OK	12.5092	0	-4.61346	-nan	0.0001	0.0159159	yes
XLOC_008113	XLOC_008113	-	jgi|Necha2|85203|fgenesh1_pg.sca_6_chr9_2_0000666:25-156	FSOAMP	FSONTC	OK	156.189	0	-4.61346	-nan	5e-05	0.00945943	yes
XLOC_008204	XLOC_008204	-	jgi|Necha2|86652|fgenesh1_pg.sca_32_chr11_3_0000047:186-618	FSOAMP	FSONTC	OK	5.06217	0	-4.61346	-nan	0.0001	0.0159159	yes
XLOC_001837	XLOC_001837	-	jgi|Necha2|30607|e_gw1.1.1313.1:2122-2355	FSOAMP	FSONTC	OK	0	12.6783	3.26775	-nan	5e-05	0.00945943	yes
XLOC_003659	XLOC_003659	-	jgi|Necha2|46823|e_gw1.10.4.1:2927-3127	FSOAMP	FSONTC	OK	0	18.3907	3.26775	-nan	5e-05	0.00945943	yes
XLOC_004219	XLOC_004219	-	jgi|Necha2|52965|e_gw1.40.123.1:1773-1954	FSOAMP	FSONTC	OK	0	33.673	3.26775	-nan	5e-05	0.00945943	yes
XLOC_005574	XLOC_005574	-	jgi|Necha2|66028|estExt_Genewise1Plus.C_sca_18_chr2_1_00150:10-119	FSOAMP	FSONTC	OK	0	310.259	3.26775	-nan	0.00015	0.0206034	yes
XLOC_001206	XLOC_001206	-	jgi|Necha2|105195|estExt_fgenesh1_pm.C_sca_27_chr9_1_00003:49-831	FSOAMP	FSONTC	OK	12368.1	252.63	-5.61346	-4.79326	5e-05	0.00945943	yes
XLOC_008831	XLOC_008831	-	jgi|Necha2|91966|fgenesh1_pm.sca_2_chr3_3_0000366:1-1109	FSOAMP	FSONTC	OK	167.851	4.45124	-5.23683	-5.37278	5e-05	0.00945943	yes
XLOC_008188	XLOC_008188	-	jgi|Necha2|85958|fgenesh1_pg.sca_14_chr10_3_0000153:2-434	FSOAMP	FSONTC	OK	150.142	4.48153	-5.06619	-4.41617	0.0004	0.0405131	yes
XLOC_005099	XLOC_005099	-	jgi|Necha2|62727|estExt_Genewise1.C_sca_10_chr8_2_00237:122-497	FSOAMP	FSONTC	OK	213.532	7.76623	-4.78109	-4.79321	5e-05	0.00945943	yes
XLOC_002486	XLOC_002486	-	jgi|Necha2|34956|e_gw1.44.94.1:41-1633	FSOAMP	FSONTC	OK	298.161	11.0337	-4.75611	-5.45117	5e-05	0.00945943	yes
XLOC_005814	XLOC_005814	-	jgi|Necha2|67443|estExt_Genewise1Plus.C_sca_2_chr3_3_01017:103-2038	FSOAMP	FSONTC	OK	1595.26	60.8438	-4.71253	-5.03145	5e-05	0.00945943	yes
XLOC_003265	XLOC_003265	-	jgi|Necha2|41350|e_gw1.5.1512.1:147-566	FSOAMP	FSONTC	OK	107.55	4.27547	-4.65278	-3.90322	0.00025	0.0291483	yes
XLOC_003803	XLOC_003803	-	jgi|Necha2|48037|e_gw1.27.204.1:525-1309	FSOAMP	FSONTC	OK	138.84	6.74278	-4.36393	-3.97151	5e-05	0.00945943	yes
XLOC_008280	XLOC_008280	-	jgi|Necha2|89600|fgenesh1_pm.sca_8_chr1_1_0000037:0-953	FSOAMP	FSONTC	OK	427.797	21.7889	-4.29526	-5.28201	5e-05	0.00945943	yes
XLOC_005365	XLOC_005365	-	jgi|Necha2|64827|estExt_Genewise1.C_sca_28_chr13_5_00070:95-1094	FSOAMP	FSONTC	OK	4.67446	90.0438	4.26775	4.0473	5e-05	0.00945943	yes
XLOC_004416	XLOC_004416	-	jgi|Necha2|57605|estExt_Genewise1.C_sca_1_chr1_3_01119:54-1232	FSOAMP	FSONTC	OK	150.245	7.89317	-4.25056	-4.57848	5e-05	0.00945943	yes
XLOC_005754	XLOC_005754	-	jgi|Necha2|67133|estExt_Genewise1Plus.C_sca_2_chr3_3_00397:0-1960	FSOAMP	FSONTC	OK	12723.3	800.082	-3.99118	-4.29729	5e-05	0.00945943	yes
XLOC_008500	XLOC_008500	-	jgi|Necha2|90242|fgenesh1_pm.sca_1_chr1_3_0000375:1326-1860	FSOAMP	FSONTC	OK	212.025	13.7946	-3.94206	-4.08194	5e-05	0.00945943	yes
XLOC_004902	XLOC_004902	-	jgi|Necha2|61067|estExt_Genewise1.C_sca_5_chr5_3_00280:11-2004	FSOAMP	FSONTC	OK	913.46	59.8928	-3.93089	-4.29475	5e-05	0.00945943	yes
XLOC_003562	XLOC_003562	-	jgi|Necha2|45493|e_gw1.9.706.1:0-861	FSOAMP	FSONTC	OK	117.354	7.87982	-3.89656	-4.11259	5e-05	0.00945943	yes
XLOC_000613	XLOC_000613	-	jgi|Necha2|102651|estExt_fgenesh1_kg.C_sca_5_chr5_3_00024:350-662	FSOAMP	FSONTC	OK	361.256	24.3658	-3.89009	-4.09871	5e-05	0.00945943	yes
XLOC_009526	XLOC_009526	-	jgi|Necha2|96330|fgenesh1_pm.sca_6_chr9_2_0000288:2631-3813	FSOAMP	FSONTC	OK	52.7794	3.56389	-3.88845	-3.9596	5e-05	0.00945943	yes
XLOC_000612	XLOC_000612	-	jgi|Necha2|102651|estExt_fgenesh1_kg.C_sca_5_chr5_3_00024:68-245	FSOAMP	FSONTC	OK	793.983	53.7704	-3.88422	-5.57745	0.00025	0.0291483	yes
XLOC_000563	XLOC_000563	-	jgi|Necha2|102426|estExt_fgenesh1_kg.C_sca_18_chr2_1_00017:234-1069	FSOAMP	FSONTC	OK	334.951	23.1518	-3.85475	-3.94302	5e-05	0.00945943	yes
XLOC_004228	XLOC_004228	-	jgi|Necha2|53388|e_gw1.29.77.1:146-380	FSOAMP	FSONTC	OK	366.962	25.9423	-3.82225	-3.49617	0.0001	0.0159159	yes
XLOC_005116	XLOC_005116	-	jgi|Necha2|62839|estExt_Genewise1.C_sca_10_chr8_2_00492:1724-2272	FSOAMP	FSONTC	OK	226.452	16.1613	-3.80859	-4.48557	5e-05	0.00945943	yes
XLOC_007923	XLOC_007923	-	jgi|Necha2|83418|fgenesh1_pg.sca_17_chr8_1_0000191:180-458	FSOAMP	FSONTC	OK	214.961	16.0897	-3.73986	-3.81494	5e-05	0.00945943	yes
XLOC_003578	XLOC_003578	-	jgi|Necha2|46030|e_gw1.17.482.1:131-506	FSOAMP	FSONTC	OK	401.756	30.5683	-3.71621	-3.63496	5e-05	0.00945943	yes
XLOC_006725	XLOC_006725	-	jgi|Necha2|73052|estExt_Genewise1Plus.C_sca_15_chr12_5_00497:1347-1644	FSOAMP	FSONTC	OK	683.007	52.1589	-3.71092	-4.03739	5e-05	0.00945943	yes
XLOC_007674	XLOC_007674	-	jgi|Necha2|79543|fgenesh1_pg.sca_19_chr4_3_0000051:0-969	FSOAMP	FSONTC	OK	1038.15	79.5031	-3.70686	-4.0405	5e-05	0.00945943	yes
XLOC_004630	XLOC_004630	-	jgi|Necha2|59124|estExt_Genewise1.C_sca_2_chr3_3_00814:38-1139	FSOAMP	FSONTC	OK	173.164	13.9618	-3.63259	-3.66619	5e-05	0.00945943	yes
XLOC_008503	XLOC_008503	-	jgi|Necha2|90246|fgenesh1_pm.sca_1_chr1_3_0000379:0-461	FSOAMP	FSONTC	OK	246.129	20.0332	-3.61895	-3.88655	5e-05	0.00945943	yes
XLOC_007924	XLOC_007924	-	jgi|Necha2|83418|fgenesh1_pg.sca_17_chr8_1_0000191:558-887	FSOAMP	FSONTC	OK	154.38	13.172	-3.55095	-3.72659	0.00015	0.0206034	yes
XLOC_003579	XLOC_003579	-	jgi|Necha2|46030|e_gw1.17.482.1:699-986	FSOAMP	FSONTC	OK	244.508	21.865	-3.48319	-3.24776	0.0001	0.0159159	yes
XLOC_004547	XLOC_004547	-	jgi|Necha2|58572|estExt_Genewise1.C_sca_18_chr2_1_02121:844-1477	FSOAMP	FSONTC	OK	550.541	51.6506	-3.41399	-3.93393	5e-05	0.00945943	yes
XLOC_008505	XLOC_008505	-	jgi|Necha2|90246|fgenesh1_pm.sca_1_chr1_3_0000379:995-1895	FSOAMP	FSONTC	OK	144.671	13.9688	-3.37249	-3.69535	5e-05	0.00945943	yes
XLOC_006225	XLOC_006225	-	jgi|Necha2|69757|estExt_Genewise1Plus.C_sca_5_chr5_3_01357:0-1536	FSOAMP	FSONTC	OK	944.155	91.7033	-3.36398	-3.70498	5e-05	0.00945943	yes
XLOC_001347	XLOC_001347	-	jgi|Necha2|105936|fgeneshN1_pm.sca_26_chr2_2_0000010:701-1035	FSOAMP	FSONTC	OK	528.218	52.7278	-3.3245	-3.73296	5e-05	0.00945943	yes
XLOC_006494	XLOC_006494	-	jgi|Necha2|71298|estExt_Genewise1Plus.C_sca_11_chr8_3_00101:598-1411	FSOAMP	FSONTC	OK	166.499	16.6774	-3.31955	-3.34555	0.0001	0.0159159	yes
XLOC_008817	XLOC_008817	-	jgi|Necha2|91891|fgenesh1_pm.sca_2_chr3_3_0000291:0-1034	FSOAMP	FSONTC	OK	186.427	18.9758	-3.29638	-3.22349	0.00015	0.0206034	yes
XLOC_003471	XLOC_003471	-	jgi|Necha2|43700|e_gw1.20.1277.1:0-323	FSOAMP	FSONTC	OK	103.658	10.5594	-3.29523	-3.38437	0.00025	0.0291483	yes
XLOC_001317	XLOC_001317	-	jgi|Necha2|105740|estExt_fgenesh1_pm.C_sca_28_chr13_5_00081:564-752	FSOAMP	FSONTC	OK	545.541	56.563	-3.26976	-3.37105	0.00015	0.0206034	yes
XLOC_004338	XLOC_004338	-	jgi|Necha2|57070|estExt_Genewise1.C_sca_8_chr1_1_00729:73-1416	FSOAMP	FSONTC	OK	76.2698	8.0327	-3.24716	-3.505	5e-05	0.00945943	yes
XLOC_001316	XLOC_001316	-	jgi|Necha2|105740|estExt_fgenesh1_pm.C_sca_28_chr13_5_00081:13-315	FSOAMP	FSONTC	OK	230.205	24.3944	-3.2383	-3.20935	5e-05	0.00945943	yes
XLOC_000448	XLOC_000448	-	jgi|Necha2|101921|estExt_fgenesh1_pg.C_sca_14_chr10_3_00151:686-1345	FSOAMP	FSONTC	OK	95.5783	10.2965	-3.21452	-3.3104	5e-05	0.00945943	yes
XLOC_004546	XLOC_004546	-	jgi|Necha2|58572|estExt_Genewise1.C_sca_18_chr2_1_02121:113-737	FSOAMP	FSONTC	OK	565.903	61.4196	-3.20378	-3.73093	5e-05	0.00945943	yes
XLOC_007919	XLOC_007919	-	jgi|Necha2|83392|fgenesh1_pg.sca_17_chr8_1_0000165:5-480	FSOAMP	FSONTC	OK	254.493	27.6658	-3.20145	-3.16612	0.00015	0.0206034	yes
XLOC_007384	XLOC_007384	-	jgi|Necha2|77393|fgenesh1_pg.sca_2_chr3_3_0000115:123-577	FSOAMP	FSONTC	OK	166.007	18.8858	-3.13587	-3.21537	0.0001	0.0159159	yes
XLOC_002366	XLOC_002366	-	jgi|Necha2|33808|e_gw1.18.2042.1:0-1433	FSOAMP	FSONTC	OK	718.227	83.7313	-3.1006	-3.21968	0.0002	0.0253848	yes
XLOC_009600	XLOC_009600	-	jgi|Necha2|96866|fgenesh1_pm.sca_14_chr10_3_0000060:12-422	FSOAMP	FSONTC	OK	293.226	34.6955	-3.0792	-3.32134	5e-05	0.00945943	yes
XLOC_009313	XLOC_009313	-	jgi|Necha2|94808|fgenesh1_pm.sca_94_chr7_5_0000001:485-879	FSOAMP	FSONTC	OK	115.042	13.7751	-3.06203	-3.09629	5e-05	0.00945943	yes
XLOC_006392	XLOC_006392	-	jgi|Necha2|70841|estExt_Genewise1Plus.C_sca_17_chr8_1_00301:106-795	FSOAMP	FSONTC	OK	126.209	15.1564	-3.05782	-3.35302	5e-05	0.00945943	yes
XLOC_005375	XLOC_005375	-	jgi|Necha2|64999|estExt_Genewise1.C_sca_132_unmapped0001:209-470	FSOAMP	FSONTC	OK	2416.72	308.716	-2.9687	-3.24941	5e-05	0.00945943	yes
XLOC_009886	XLOC_009886	-	jgi|Necha2|98911|estExt_fgenesh1_pg.C_sca_1_chr1_3_00795:5-462	FSOAMP	FSONTC	OK	111.068	14.2746	-2.95991	-3.11007	0.00015	0.0206034	yes
XLOC_001157	XLOC_001157	-	jgi|Necha2|104917|estExt_fgenesh1_pm.C_sca_9_chr7_10_00188:0-1460	FSOAMP	FSONTC	OK	122.27	15.8508	-2.94744	-3.12919	0.0001	0.0159159	yes
XLOC_007766	XLOC_007766	-	jgi|Necha2|80505|fgenesh1_pg.sca_5_chr5_3_0000304:0-343	FSOAMP	FSONTC	OK	121.307	16.3462	-2.89163	-3.16274	0.00015	0.0206034	yes
XLOC_008640	XLOC_008640	-	jgi|Necha2|90932|fgenesh1_pm.sca_18_chr2_1_0000336:4-313	FSOAMP	FSONTC	OK	270.021	36.423	-2.89015	-2.90971	0.0002	0.0253848	yes
XLOC_000447	XLOC_000447	-	jgi|Necha2|101921|estExt_fgenesh1_pg.C_sca_14_chr10_3_00151:0-212	FSOAMP	FSONTC	OK	322.814	44.0809	-2.87248	-2.88518	0.0003	0.033056	yes
XLOC_007332	XLOC_007332	-	jgi|Necha2|76673|fgenesh1_pg.sca_18_chr2_1_0000864:736-1304	FSOAMP	FSONTC	OK	98.1409	13.4308	-2.86931	-3.18891	5e-05	0.00945943	yes
XLOC_007885	XLOC_007885	-	jgi|Necha2|81884|fgenesh1_pg.sca_20_chr6_4_0000510:1-1260	FSOAMP	FSONTC	OK	821.342	124.466	-2.72223	-2.69341	0.00035	0.0369416	yes
XLOC_006394	XLOC_006394	-	jgi|Necha2|70841|estExt_Genewise1Plus.C_sca_17_chr8_1_00301:1769-2715	FSOAMP	FSONTC	OK	153.562	23.3768	-2.71567	-3.08038	0.0001	0.0159159	yes
XLOC_003814	XLOC_003814	-	jgi|Necha2|48247|e_gw1.27.37.1:630-848	FSOAMP	FSONTC	OK	369.581	56.3869	-2.71246	-2.88982	0.00035	0.0369416	yes
XLOC_009452	XLOC_009452	-	jgi|Necha2|95800|fgenesh1_pm.sca_11_chr8_3_0000142:766-1034	FSOAMP	FSONTC	OK	320.611	48.9823	-2.71049	-2.89313	0.0003	0.033056	yes
XLOC_005710	XLOC_005710	-	jgi|Necha2|66818|estExt_Genewise1Plus.C_sca_18_chr2_1_02141:2-425	FSOAMP	FSONTC	OK	114.924	17.6777	-2.70067	-3.01122	0.00025	0.0291483	yes
XLOC_006393	XLOC_006393	-	jgi|Necha2|70841|estExt_Genewise1Plus.C_sca_17_chr8_1_00301:873-1482	FSOAMP	FSONTC	OK	156.255	24.7252	-2.65985	-2.9735	0.00015	0.0206034	yes
XLOC_007333	XLOC_007333	-	jgi|Necha2|76673|fgenesh1_pg.sca_18_chr2_1_0000864:1362-2290	FSOAMP	FSONTC	OK	29.3615	4.68706	-2.64717	-2.77314	0.0004	0.0405131	yes
XLOC_005175	XLOC_005175	-	jgi|Necha2|63257|estExt_Genewise1.C_sca_27_chr9_1_00259:48-1661	FSOAMP	FSONTC	OK	301.204	49.7531	-2.59788	-2.88957	5e-05	0.00945943	yes
XLOC_002793	XLOC_002793	-	jgi|Necha2|37665|e_gw1.3.378.1:52-1391	FSOAMP	FSONTC	OK	85.0206	14.0795	-2.59421	-3.23062	5e-05	0.00945943	yes
XLOC_007021	XLOC_007021	-	jgi|Necha2|74848|fgenesh1_pg.sca_1_chr1_3_0000459:0-980	FSOAMP	FSONTC	OK	54.0637	8.97005	-2.59147	-2.82104	0.0003	0.033056	yes
XLOC_000581	XLOC_000581	-	jgi|Necha2|102552|estExt_fgenesh1_kg.C_sca_3_chr4_2_00039:335-941	FSOAMP	FSONTC	OK	337.23	55.972	-2.59096	-2.83548	0.00035	0.0369416	yes
XLOC_006134	XLOC_006134	-	jgi|Necha2|69214|estExt_Genewise1Plus.C_sca_5_chr5_3_00094:24-1116	FSOAMP	FSONTC	OK	65.7951	11.0858	-2.56927	-3.00338	5e-05	0.00945943	yes
XLOC_005105	XLOC_005105	-	jgi|Necha2|62784|estExt_Genewise1.C_sca_10_chr8_2_00368:259-1306	FSOAMP	FSONTC	OK	350.287	59.7223	-2.5522	-2.86427	0.00025	0.0291483	yes
XLOC_000838	XLOC_000838	-	jgi|Necha2|103549|estExt_fgenesh1_pm.C_sca_18_chr2_1_00695:906-3913	FSOAMP	FSONTC	OK	79.1607	13.6018	-2.54098	-2.69304	0.00035	0.0369416	yes
XLOC_004802	XLOC_004802	-	jgi|Necha2|60425|estExt_Genewise1.C_sca_3_chr4_2_01485:59-1208	FSOAMP	FSONTC	OK	123.588	21.4009	-2.5298	-2.83067	0.0003	0.033056	yes
XLOC_010062	XLOC_010062	-	jgi|Necha2|99589|estExt_fgenesh1_pg.C_sca_2_chr3_3_00394:2136-2679	FSOAMP	FSONTC	OK	181.793	32.2989	-2.49274	-2.79714	0.00015	0.0206034	yes
XLOC_009797	XLOC_009797	-	jgi|Necha2|98772|estExt_fgenesh1_pg.C_sca_1_chr1_3_00313:1201-2397	FSOAMP	FSONTC	OK	125.089	22.5174	-2.47384	-2.72255	0.00025	0.0291483	yes
XLOC_005079	XLOC_005079	-	jgi|Necha2|62632|estExt_Genewise1.C_sca_17_chr8_1_00470:14-2987	FSOAMP	FSONTC	OK	15.5362	2.88144	-2.43078	-2.72497	0.0001	0.0159159	yes
XLOC_005895	XLOC_005895	-	jgi|Necha2|67950|estExt_Genewise1Plus.C_sca_3_chr4_2_00023:0-1940	FSOAMP	FSONTC	OK	201.197	38.2576	-2.39479	-2.6716	0.0005	0.0486748	yes
XLOC_000006	XLOC_000006	-	jgi|Necha2|100017|estExt_fgenesh1_pg.C_sca_3_chr4_2_00337:6-918	FSOAMP	FSONTC	OK	14.8097	77.5605	2.38878	2.84414	0.00025	0.0291483	yes
XLOC_000030	XLOC_000030	-	jgi|Necha2|100063|estExt_fgenesh1_pg.C_sca_3_chr4_2_00423:1-1449	FSOAMP	FSONTC	OK	90.165	18.1171	-2.31522	-2.79887	5e-05	0.00945943	yes
XLOC_007733	XLOC_007733	-	jgi|Necha2|80283|fgenesh1_pg.sca_5_chr5_3_0000082:781-1665	FSOAMP	FSONTC	OK	81.0419	16.5719	-2.28993	-2.48342	0.0002	0.0253848	yes
XLOC_005438	XLOC_005438	-	jgi|Necha2|65406|estExt_Genewise1Plus.C_sca_1_chr1_3_00088:1665-2831	FSOAMP	FSONTC	OK	178.17	36.4344	-2.28988	-2.54584	0.0005	0.0486748	yes
XLOC_009976	XLOC_009976	-	jgi|Necha2|99160|estExt_fgenesh1_pg.C_sca_18_chr2_1_00441:255-468	FSOAMP	FSONTC	OK	717.447	3504.36	2.28821	2.80753	0.0002	0.0253848	yes
XLOC_006496	XLOC_006496	-	jgi|Necha2|71309|estExt_Genewise1Plus.C_sca_11_chr8_3_00126:373-2310	FSOAMP	FSONTC	OK	155.858	32.1605	-2.27687	-2.77527	0.0001	0.0159159	yes
XLOC_009937	XLOC_009937	-	jgi|Necha2|99005|estExt_fgenesh1_pg.C_sca_1_chr1_3_01102:717-1490	FSOAMP	FSONTC	OK	159.385	32.9983	-2.27206	-2.60801	0.0002	0.0253848	yes
XLOC_010036	XLOC_010036	-	jgi|Necha2|99505|estExt_fgenesh1_pg.C_sca_2_chr3_3_00161:0-994	FSOAMP	FSONTC	OK	39.5023	187.906	2.25	2.6158	0.0005	0.0486748	yes
XLOC_005482	XLOC_005482	-	jgi|Necha2|65650|estExt_Genewise1Plus.C_sca_1_chr1_3_00612:39-1841	FSOAMP	FSONTC	OK	7.25945	34.376	2.24347	2.72312	0.0004	0.0405131	yes
XLOC_000895	XLOC_000895	-	jgi|Necha2|103727|estExt_fgenesh1_pm.C_sca_2_chr3_3_00212:4-1388	FSOAMP	FSONTC	OK	81.6587	17.3331	-2.23607	-2.64837	0.0002	0.0253848	yes
XLOC_006020	XLOC_006020	-	jgi|Necha2|68517|estExt_Genewise1Plus.C_sca_3_chr4_2_01150:283-1045	FSOAMP	FSONTC	OK	78.977	17.1073	-2.20682	-2.56765	0.00045	0.0451215	yes
XLOC_006021	XLOC_006021	-	jgi|Necha2|68517|estExt_Genewise1Plus.C_sca_3_chr4_2_01150:1110-2607	FSOAMP	FSONTC	OK	236.316	52.4501	-2.1717	-2.56177	0.0004	0.0405131	yes
XLOC_008637	XLOC_008637	-	jgi|Necha2|90927|fgenesh1_pm.sca_18_chr2_1_0000331:2886-3320	FSOAMP	FSONTC	OK	262.138	59.5729	-2.1376	-2.56387	0.0003	0.033056	yes
XLOC_003843	XLOC_003843	-	jgi|Necha2|48581|e_gw1.6.807.1:1-162	FSOAMP	FSONTC	OK	1572.93	362.818	-2.11613	-2.62095	0.00015	0.0206034	yes

***N. solani* + PSC**

test_id	gene_id	gene	locus	sample_1	sample_2	status	value_1	value_2	log2(fold_change)	test_stat	p_value	q_value	significant
XLOC_000552	XLOC_000552	-	jgi|Necha2|102402|estExt_fgenesh1_kg.C_sca_1_chr1_3_00089:278-405	FSOPSC	FSONTC	OK	1230.8	0	-6.22869	-nan	5e-05	0.00952453	yes
XLOC_001682	XLOC_001682	-	jgi|Necha2|25451|gw1.20.1344.1:243-443	FSOPSC	FSONTC	OK	33.4792	0	-6.22869	-nan	5e-05	0.00952453	yes
XLOC_002261	XLOC_002261	-	jgi|Necha2|33136|e_gw1.18.384.1:717-1126	FSOPSC	FSONTC	OK	24.8474	0	-6.22869	-nan	5e-05	0.00952453	yes
XLOC_002311	XLOC_002311	-	jgi|Necha2|33473|e_gw1.18.2143.1:409-620	FSOPSC	FSONTC	OK	72.6043	0	-6.22869	-nan	5e-05	0.00952453	yes
XLOC_002997	XLOC_002997	-	jgi|Necha2|38820|e_gw1.3.1272.1:1042-1189	FSOPSC	FSONTC	OK	41.9487	0	-6.22869	-nan	0.00025	0.0327792	yes
XLOC_003143	XLOC_003143	-	jgi|Necha2|40389|e_gw1.12.678.1:606-826	FSOPSC	FSONTC	OK	71.4906	0	-6.22869	-nan	5e-05	0.00952453	yes
XLOC_003433	XLOC_003433	-	jgi|Necha2|43313|e_gw1.20.1298.1:75-380	FSOPSC	FSONTC	OK	38.9336	0	-6.22869	-nan	5e-05	0.00952453	yes
XLOC_003537	XLOC_003537	-	jgi|Necha2|44802|e_gw1.58.25.1:834-980	FSOPSC	FSONTC	OK	238.59	0	-6.22869	-nan	5e-05	0.00952453	yes
XLOC_003538	XLOC_003538	-	jgi|Necha2|44971|e_gw1.9.166.1:12-388	FSOPSC	FSONTC	OK	66.7403	0	-6.22869	-nan	5e-05	0.00952453	yes
XLOC_003539	XLOC_003539	-	jgi|Necha2|44971|e_gw1.9.166.1:646-761	FSOPSC	FSONTC	OK	264.286	0	-6.22869	-nan	0.00035	0.0406161	yes
XLOC_004024	XLOC_004024	-	jgi|Necha2|49776|e_gw1.43.147.1:925-1447	FSOPSC	FSONTC	OK	3.72042	0	-6.22869	-nan	0.0002	0.0288457	yes
XLOC_004299	XLOC_004299	-	jgi|Necha2|56078|e_gw1.100.10.1:1108-1494	FSOPSC	FSONTC	OK	7.88935	0	-6.22869	-nan	5e-05	0.00952453	yes
XLOC_005163	XLOC_005163	-	jgi|Necha2|63187|estExt_Genewise1.C_sca_11_chr8_3_00512:4-277	FSOPSC	FSONTC	OK	15.2729	0	-6.22869	-nan	5e-05	0.00952453	yes
XLOC_005165	XLOC_005165	-	jgi|Necha2|63187|estExt_Genewise1.C_sca_11_chr8_3_00512:1644-1894	FSOPSC	FSONTC	OK	29.8724	0	-6.22869	-nan	5e-05	0.00952453	yes
XLOC_005579	XLOC_005579	-	jgi|Necha2|66087|estExt_Genewise1Plus.C_sca_18_chr2_1_00400:1104-1334	FSOPSC	FSONTC	OK	15.1068	0	-6.22869	-nan	0.00015	0.0222706	yes
XLOC_007794	XLOC_007794	-	jgi|Necha2|80681|fgenesh1_pg.sca_5_chr5_3_0000480:161-351	FSOPSC	FSONTC	OK	93.9847	0	-6.22869	-nan	5e-05	0.00952453	yes
XLOC_007903	XLOC_007903	-	jgi|Necha2|82460|fgenesh1_pg.sca_58_chr7_9_0000044:226-526	FSOPSC	FSONTC	OK	15.7371	0	-6.22869	-nan	5e-05	0.00952453	yes
XLOC_008218	XLOC_008218	-	jgi|Necha2|87168|fgenesh1_pg.sca_41_chr12_2_0000001:20-335	FSOPSC	FSONTC	OK	21.9958	0	-6.22869	-nan	5e-05	0.00952453	yes
XLOC_008238	XLOC_008238	-	jgi|Necha2|88214|fgenesh1_pg.sca_28_chr13_5_0000116:999-1112	FSOPSC	FSONTC	OK	376.334	0	-6.22869	-nan	0.0001	0.0171119	yes
XLOC_009105	XLOC_009105	-	jgi|Necha2|93326|fgenesh1_pm.sca_12_chr5_2_0000143:386-993	FSOPSC	FSONTC	OK	22.6752	0	-6.22869	-nan	5e-05	0.00952453	yes
XLOC_009106	XLOC_009106	-	jgi|Necha2|93326|fgenesh1_pm.sca_12_chr5_2_0000143:1497-2042	FSOPSC	FSONTC	OK	36.8702	0	-6.22869	-nan	5e-05	0.00952453	yes
XLOC_009604	XLOC_009604	-	jgi|Necha2|96869|fgenesh1_pm.sca_14_chr10_3_0000063:1384-1666	FSOPSC	FSONTC	OK	10.1246	0	-6.22869	-nan	0.00015	0.0222706	yes
XLOC_009642	XLOC_009642	-	jgi|Necha2|97646|fgenesh1_pm.sca_29_chr12_4_0000036:742-982	FSOPSC	FSONTC	OK	50.3962	0	-6.22869	-nan	5e-05	0.00952453	yes
XLOC_000663	XLOC_000663	-	jgi|Necha2|102879|estExt_fgenesh1_kg.C_sca_14_chr10_3_00008:146-257	FSOPSC	FSONTC	OK	0	397.299	5.16206	-nan	0.00025	0.0327792	yes
XLOC_001471	XLOC_001471	-	jgi|Necha2|122939|fgeneshN1_kg.sca_40_chr12_1_0000002:69-475	FSOPSC	FSONTC	OK	0	25.831	5.16206	-nan	5e-05	0.00952453	yes
XLOC_001677	XLOC_001677	-	jgi|Necha2|24316|gw1.33.342.1:577-1028	FSOPSC	FSONTC	OK	0	86.4578	5.16206	-nan	5e-05	0.00952453	yes
XLOC_001678	XLOC_001678	-	jgi|Necha2|24316|gw1.33.342.1:1177-1407	FSOPSC	FSONTC	OK	0	15.8411	5.16206	-nan	0.0003	0.0360571	yes
XLOC_003432	XLOC_003432	-	jgi|Necha2|43302|e_gw1.20.192.1:1026-1175	FSOPSC	FSONTC	OK	0	184.23	5.16206	-nan	5e-05	0.00952453	yes
XLOC_003529	XLOC_003529	-	jgi|Necha2|44426|e_gw1.84.19.1:18174-18299	FSOPSC	FSONTC	OK	0	115.927	5.16206	-nan	0.0003	0.0360571	yes
XLOC_003560	XLOC_003560	-	jgi|Necha2|45456|e_gw1.9.61.1:395-726	FSOPSC	FSONTC	OK	0	10.6179	5.16206	-nan	0.00045	0.0493826	yes
XLOC_004012	XLOC_004012	-	jgi|Necha2|49652|e_gw1.43.4.1:264-473	FSOPSC	FSONTC	OK	0	19.5779	5.16206	-nan	0.00015	0.0222706	yes
XLOC_004219	XLOC_004219	-	jgi|Necha2|52965|e_gw1.40.123.1:1773-1954	FSOPSC	FSONTC	OK	0	35.4763	5.16206	-nan	0.0001	0.0171119	yes
XLOC_004259	XLOC_004259	-	jgi|Necha2|54329|e_gw1.15.344.1:350-584	FSOPSC	FSONTC	OK	0	16.0199	5.16206	-nan	5e-05	0.00952453	yes
XLOC_005187	XLOC_005187	-	jgi|Necha2|63362|estExt_Genewise1.C_sca_6_chr9_2_00050:328-487	FSOPSC	FSONTC	OK	0	42.2147	5.16206	-nan	0.0001	0.0171119	yes
XLOC_005872	XLOC_005872	-	jgi|Necha2|67785|estExt_Genewise1Plus.C_sca_54_chr3_4_00066:117-250	FSOPSC	FSONTC	OK	0	386.777	5.16206	-nan	5e-05	0.00952453	yes
XLOC_005883	XLOC_005883	-	jgi|Necha2|67881|estExt_Genewise1Plus.C_sca_31_chr3_5_00313:940-1118	FSOPSC	FSONTC	OK	0	78.4103	5.16206	-nan	5e-05	0.00952453	yes
XLOC_006773	XLOC_006773	-	jgi|Necha2|73479|fgenesh1_kg.sca_18_chr2_1_0000031:204-696	FSOPSC	FSONTC	OK	0	24.7622	5.16206	-nan	5e-05	0.00952453	yes
XLOC_007392	XLOC_007392	-	jgi|Necha2|77516|fgenesh1_pg.sca_2_chr3_3_0000238:151-257	FSOPSC	FSONTC	OK	0	574.987	5.16206	-nan	0.0003	0.0360571	yes
XLOC_007751	XLOC_007751	-	jgi|Necha2|80428|fgenesh1_pg.sca_5_chr5_3_0000227:437-775	FSOPSC	FSONTC	OK	0	8.31347	5.16206	-nan	0.00025	0.0327792	yes
XLOC_008222	XLOC_008222	-	jgi|Necha2|87276|fgenesh1_pg.sca_41_chr12_2_0000109:234-410	FSOPSC	FSONTC	OK	0	99.8525	5.16206	-nan	5e-05	0.00952453	yes
XLOC_009894	XLOC_009894	-	jgi|Necha2|98919|estExt_fgenesh1_pg.C_sca_1_chr1_3_00821:749-980	FSOPSC	FSONTC	OK	0	52.461	5.16206	-nan	5e-05	0.00952453	yes
XLOC_009325	XLOC_009325	-	jgi|Necha2|95089|fgenesh1_pm.sca_9_chr7_10_0000189:17-1463	FSOPSC	FSONTC	OK	265.488	1.77007	-7.22869	-6.0369	5e-05	0.00952453	yes
XLOC_000005	XLOC_000005	-	jgi|Necha2|100013|estExt_fgenesh1_pg.C_sca_3_chr4_2_00315:73-477	FSOPSC	FSONTC	OK	7.97737	571.249	6.16206	4.70705	5e-05	0.00952453	yes
XLOC_008182	XLOC_008182	-	jgi|Necha2|85938|fgenesh1_pg.sca_14_chr10_3_0000133:36-490	FSOPSC	FSONTC	OK	1013.02	14.8658	-6.09052	-3.74566	0.00025	0.0327792	yes
XLOC_000603	XLOC_000603	-	jgi|Necha2|102607|estExt_fgenesh1_kg.C_sca_12_chr5_2_00004:16-351	FSOPSC	FSONTC	OK	10.8652	718.626	6.04745	5.08079	5e-05	0.00952453	yes
XLOC_002486	XLOC_002486	-	jgi|Necha2|34956|e_gw1.44.94.1:41-1633	FSOPSC	FSONTC	OK	573.214	11.4339	-5.64769	-3.70934	0.0001	0.0171119	yes
XLOC_005044	XLOC_005044	-	jgi|Necha2|62104|estExt_Genewise1.C_sca_20_chr6_4_00873:1119-2459	FSOPSC	FSONTC	OK	4.92305	207.173	5.39514	4.25114	5e-05	0.00952453	yes
XLOC_002741	XLOC_002741	-	jgi|Necha2|36929|e_gw1.54.143.1:9-540	FSOPSC	FSONTC	OK	6.20841	257.892	5.3764	3.96606	0.00035	0.0406161	yes
XLOC_007481	XLOC_007481	-	jgi|Necha2|78548|fgenesh1_pg.sca_60_chr3_6_0000052:5-1500	FSOPSC	FSONTC	OK	50.9153	1.33305	-5.2553	-3.76084	5e-05	0.00952453	yes
XLOC_009976	XLOC_009976	-	jgi|Necha2|99160|estExt_fgenesh1_pg.C_sca_18_chr2_1_00441:255-468	FSOPSC	FSONTC	OK	116.995	3661.86	4.96806	4.15744	5e-05	0.00952453	yes
XLOC_007021	XLOC_007021	-	jgi|Necha2|74848|fgenesh1_pg.sca_1_chr1_3_0000459:0-980	FSOPSC	FSONTC	OK	257.799	9.32901	-4.78838	-3.6819	5e-05	0.00952453	yes
XLOC_000087	XLOC_000087	-	jgi|Necha2|100233|estExt_fgenesh1_pg.C_sca_3_chr4_2_00817:168-1487	FSOPSC	FSONTC	OK	6.32715	173.006	4.77312	4.20443	5e-05	0.00952453	yes
XLOC_006317	XLOC_006317	-	jgi|Necha2|70270|estExt_Genewise1Plus.C_sca_20_chr6_4_00711:469-1632	FSOPSC	FSONTC	OK	199.208	7.30589	-4.76907	-3.64174	5e-05	0.00952453	yes
XLOC_005365	XLOC_005365	-	jgi|Necha2|64827|estExt_Genewise1.C_sca_28_chr13_5_00070:95-1094	FSOPSC	FSONTC	OK	3.61426	94.9233	4.71499	3.50672	5e-05	0.00952453	yes
XLOC_009708	XLOC_009708	-	jgi|Necha2|98619|estExt_fgenesh1_pg.C_sca_8_chr1_1_00396:793-1224	FSOPSC	FSONTC	OK	186.451	7.22578	-4.6895	-3.63681	0.00025	0.0327792	yes
XLOC_007661	XLOC_007661	-	jgi|Necha2|79446|fgenesh1_pg.sca_3_chr4_2_0000847:732-931	FSOPSC	FSONTC	OK	765.823	31.3896	-4.60865	-3.89533	5e-05	0.00952453	yes
XLOC_009707	XLOC_009707	-	jgi|Necha2|98619|estExt_fgenesh1_pg.C_sca_8_chr1_1_00396:158-647	FSOPSC	FSONTC	OK	410.869	17.9662	-4.51532	-3.73513	5e-05	0.00952453	yes
XLOC_001015	XLOC_001015	-	jgi|Necha2|104286|estExt_fgenesh1_pm.C_sca_19_chr4_3_00117:1200-1651	FSOPSC	FSONTC	OK	11.7154	255.135	4.44478	3.39336	0.0004	0.0448711	yes
XLOC_006791	XLOC_006791	-	jgi|Necha2|73679|fgenesh1_kg.sca_5_chr5_3_0000013:0-768	FSOPSC	FSONTC	OK	27.9379	595.858	4.41467	3.73445	0.00015	0.0222706	yes
XLOC_004957	XLOC_004957	-	jgi|Necha2|61350|estExt_Genewise1.C_sca_5_chr5_3_00888:62-644	FSOPSC	FSONTC	OK	114.362	2345.97	4.35851	3.47484	5e-05	0.00952453	yes
XLOC_007660	XLOC_007660	-	jgi|Necha2|79446|fgenesh1_pg.sca_3_chr4_2_0000847:0-498	FSOPSC	FSONTC	OK	298.98	14.6388	-4.35217	-3.86312	5e-05	0.00952453	yes
XLOC_010036	XLOC_010036	-	jgi|Necha2|99505|estExt_fgenesh1_pg.C_sca_2_chr3_3_00161:0-994	FSOPSC	FSONTC	OK	9.82265	196.776	4.3243	3.78977	5e-05	0.00952453	yes
XLOC_000654	XLOC_000654	-	jgi|Necha2|102832|estExt_fgenesh1_kg.C_sca_6_chr9_2_00037:410-597	FSOPSC	FSONTC	OK	181.233	3359.76	4.21244	3.56624	5e-05	0.00952453	yes
XLOC_008441	XLOC_008441	-	jgi|Necha2|90089|fgenesh1_pm.sca_1_chr1_3_0000222:391-730	FSOPSC	FSONTC	OK	8.68867	153.248	4.14059	3.36237	0.0003	0.0360571	yes
XLOC_005580	XLOC_005580	-	jgi|Necha2|66087|estExt_Genewise1Plus.C_sca_18_chr2_1_00400:1441-2721	FSOPSC	FSONTC	OK	256.592	15.0567	-4.091	-3.19382	5e-05	0.00952453	yes
XLOC_010037	XLOC_010037	-	jgi|Necha2|99505|estExt_fgenesh1_pg.C_sca_2_chr3_3_00161:1498-1805	FSOPSC	FSONTC	OK	18.9733	319.825	4.07524	3.46468	5e-05	0.00952453	yes
XLOC_005578	XLOC_005578	-	jgi|Necha2|66087|estExt_Genewise1Plus.C_sca_18_chr2_1_00400:540-954	FSOPSC	FSONTC	OK	214.525	12.9383	-4.05142	-3.161	0.00015	0.0222706	yes
XLOC_000538	XLOC_000538	-	jgi|Necha2|102387|estExt_fgenesh1_kg.C_sca_1_chr1_3_00067:187-580	FSOPSC	FSONTC	OK	226.116	13.7171	-4.04301	-3.0418	0.00015	0.0222706	yes
XLOC_006990	XLOC_006990	-	jgi|Necha2|74762|fgenesh1_pg.sca_1_chr1_3_0000373:331-899	FSOPSC	FSONTC	OK	6.55904	107.576	4.03573	3.36362	0.0004	0.0448711	yes
XLOC_003358	XLOC_003358	-	jgi|Necha2|42213|e_gw1.33.369.1:777-1038	FSOPSC	FSONTC	OK	847.615	52.6814	-4.00804	-3.16959	0.0003	0.0360571	yes
XLOC_000557	XLOC_000557	-	jgi|Necha2|102409|estExt_fgenesh1_kg.C_sca_1_chr1_3_00101:305-851	FSOPSC	FSONTC	OK	503.022	32.7374	-3.94161	-3.43463	0.00015	0.0222706	yes
XLOC_006821	XLOC_006821	-	jgi|Necha2|74068|fgenesh1_pg.sca_8_chr1_1_0000099:3870-4182	FSOPSC	FSONTC	OK	17.8562	273.618	3.93766	3.06456	0.0002	0.0288457	yes
XLOC_003567	XLOC_003567	-	jgi|Necha2|45826|e_gw1.22.250.1:0-831	FSOPSC	FSONTC	OK	5.76963	83.2153	3.8503	3.34386	0.00025	0.0327792	yes
XLOC_010153	XLOC_010153	-	jgi|Necha2|99959|estExt_fgenesh1_pg.C_sca_3_chr4_2_00213:1111-1719	FSOPSC	FSONTC	OK	29.6908	426.212	3.84348	3.4085	5e-05	0.00952453	yes
XLOC_008566	XLOC_008566	-	jgi|Necha2|90431|fgenesh1_pm.sca_1_chr1_3_0000564:23-641	FSOPSC	FSONTC	OK	8.98058	126.318	3.81411	3.47199	5e-05	0.00952453	yes
XLOC_004275	XLOC_004275	-	jgi|Necha2|54849|e_gw1.28.37.1:102-647	FSOPSC	FSONTC	OK	9.37239	131.58	3.81138	3.24564	0.00035	0.0406161	yes
XLOC_000569	XLOC_000569	-	jgi|Necha2|102497|estExt_fgenesh1_kg.C_sca_2_chr3_3_00033:105-407	FSOPSC	FSONTC	OK	110.891	1539.34	3.7951	2.93889	5e-05	0.00952453	yes
XLOC_006100	XLOC_006100	-	jgi|Necha2|68940|estExt_Genewise1Plus.C_sca_12_chr5_2_00169:1854-2261	FSOPSC	FSONTC	OK	312.732	23.2035	-3.75251	-3.05302	0.00015	0.0222706	yes
XLOC_002541	XLOC_002541	-	jgi|Necha2|35479|e_gw1.2.978.1:738-1001	FSOPSC	FSONTC	OK	45.5849	611.472	3.74566	2.84381	0.0001	0.0171119	yes
XLOC_010152	XLOC_010152	-	jgi|Necha2|99959|estExt_fgenesh1_pg.C_sca_3_chr4_2_00213:0-931	FSOPSC	FSONTC	OK	48.9969	653.994	3.73852	3.24659	5e-05	0.00952453	yes
XLOC_006560	XLOC_006560	-	jgi|Necha2|71809|estExt_Genewise1Plus.C_sca_6_chr9_2_00398:105-768	FSOPSC	FSONTC	OK	612.962	49.4323	-3.63227	-2.96968	0.00045	0.0493826	yes
XLOC_002540	XLOC_002540	-	jgi|Necha2|35479|e_gw1.2.978.1:3-596	FSOPSC	FSONTC	OK	18.3822	226.95	3.626	2.7867	5e-05	0.00952453	yes
XLOC_000460	XLOC_000460	-	jgi|Necha2|101975|estExt_fgenesh1_pg.C_sca_52_chr10_4_00053:3-798	FSOPSC	FSONTC	OK	13.2511	154.918	3.54732	3.23859	5e-05	0.00952453	yes
XLOC_001174	XLOC_001174	-	jgi|Necha2|105062|estExt_fgenesh1_pm.C_sca_10_chr8_2_00210:55-1298	FSOPSC	FSONTC	OK	9.80108	110.965	3.50102	3.30271	5e-05	0.00952453	yes
XLOC_004276	XLOC_004276	-	jgi|Necha2|54849|e_gw1.28.37.1:747-1409	FSOPSC	FSONTC	OK	12.7048	142.503	3.48754	3.20554	0.0001	0.0171119	yes
XLOC_007621	XLOC_007621	-	jgi|Necha2|79258|fgenesh1_pg.sca_3_chr4_2_0000659:0-775	FSOPSC	FSONTC	OK	82.051	7.64092	-3.4247	-2.63928	0.0004	0.0448711	yes
XLOC_000217	XLOC_000217	-	jgi|Necha2|100823|estExt_fgenesh1_pg.C_sca_20_chr6_4_00077:0-1049	FSOPSC	FSONTC	OK	19.2187	206.14	3.42304	3.18315	5e-05	0.00952453	yes
XLOC_006263	XLOC_006263	-	jgi|Necha2|69991|estExt_Genewise1Plus.C_sca_20_chr6_4_00057:42-577	FSOPSC	FSONTC	OK	105.142	10.2305	-3.3614	-3.1559	5e-05	0.00952453	yes
XLOC_000211	XLOC_000211	-	jgi|Necha2|100803|estExt_fgenesh1_pg.C_sca_53_chr6_3_00068:143-1182	FSOPSC	FSONTC	OK	18.2118	178.215	3.29067	2.78959	0.0003	0.0360571	yes
XLOC_001518	XLOC_001518	-	jgi|Necha2|123214|estExt_fgeneshN1_kg.C_sca_27_chr9_1_00006:842-1587	FSOPSC	FSONTC	OK	295.441	33.9964	-3.11942	-2.50467	0.00025	0.0327792	yes
XLOC_001061	XLOC_001061	-	jgi|Necha2|104483|estExt_fgenesh1_pm.C_sca_5_chr5_3_00198:171-640	FSOPSC	FSONTC	OK	122.378	957.678	2.96819	2.79797	0.0003	0.0360571	yes
XLOC_004681	XLOC_004681	-	jgi|Necha2|59530|estExt_Genewise1.C_sca_54_chr3_4_00035:29-1448	FSOPSC	FSONTC	OK	1090.57	143.605	-2.9249	-2.59048	0.00015	0.0222706	yes

Table S5. Genes differentially expressed for *F. oxysporum* and *N. solani* against AMB or PSC treatment. This table lists the gene name, GO Term, location and fold change detected in RNA-seq analysis. It only shows DEGs with some associated antifungal resistance (p-value <0.05).

Gene Name	GoTerm Blast2Go	Location	*F.oxysporum* FMR 9788		*N. solani* FMR 4291	
			AMB	PSC	AMB	PSC
Ergosterol and sterol synthesis pathway						
HBD	3-hydroxybutyryl-CoAdehydrogenase	jgi|Fusox2|20487|FOXG_04314T0:1509-2331	-6.38819	0	0	0
ERG5	C-22 sterol desaturase	jgi|Fusox2|4848|FOXG_09647T0:248-1628	5.04242	0	0	0
ERG2	C-8 sterol isomerase	jgi|Necha2|71809|estExt_Genewise1Plus.C_sca_6_chr9_2_00398:105-768	0	0	0	-3.63227
CDH	Carnitine dehydratase	jgi|Fusox2|1566|FOXG_00798T0:0-2493	-2.66066	0	0	0
CAT	Carnitine O-acetyltransferase	jgi|Fusox2|21687|FOXG_04704T0:5-1626	0	6.54621	0	0
EBP	Cholestenol Delta-isomerase	jgi|Necha2|68940|estExt_Genewise1Plus.C_sca_12_chr5_2_00169:1854-2261	0	0	0	-3.75251
EBP	Cholestenol Delta-isomerase	jgi|Fusox2|9227|FOXG_12873T0:7-998	4.28392	0	0	0
P450ome	Cyrochrome P450 monooxygenase	jgi|Fusox2|17729|FOXG_03513T0:1-2854	3.07771	0	0	0
P450ome4F5	Cytochrome P450 4F5	jgi|Fusox2|18911|FOXG_16528T0:92-1740	0	-4.34034	0	0
P450omeqxH	Cytochrome P450 monooxygenase eqxH	jgi|Fusox2|5853|FOXG_10386T0:11-1889	0	-4.40649	0	0
P450omegsfF	Cytochrome P450 monooxygenase gsfF	jgi|Fusox2|8316|FOXG_12028T0:66-1237	3.01712	0	0	0
P450omehimC	Cytochrome P450 monooxygenase himC	jgi|Necha2|78548|fgenesh1_pg.sca_60_chr3_6_0000052:5-1500	0	0	0	-5.2553
P450omeorf2	Cytochrome P450 monooxygenase orf2	jgi|Fusox2|10117|FOXG_13126T0:42-1832	3.39961	0	0	0
CPR	Cytochrome P450 oxidoreductase	jgi|Fusox2|4904|FOXG_09685T0:12-1658	5.58492	0	0	0
LIP1	Lipase 1	jgi|Necha2|95089|fgenesh1_pm.sca_9_chr7_10_0000189:17-1463	0	0	0	-7.22869
LIP4	Lipase 4	jgi|Fusox2|19536|FOXG_16927T0:390-1830	-3.43589	0	0	0
LPP	Lipid phosphate phosphatase 2	jgi|Fusox2|20549|FOXG_04356T0:78-1409	-2.71799	0	0	0
ERG6	Sterol 24-C-methyltransferase	jgi|Necha2|59530|estExt_Genewise1.C_sca_54_chr3_4_00035:29-1448	0	0	0	-2.9249
ERG6	Sterol 24-C-methyltransferase	jgi|Necha2|33136|e_gw1.18.384.1:717-1126	0	0	0	-6.22869
ERG6	Sterol 24-C-methyltransferase	jgi|Fusox2|3538|FOXG_08765T0:120-1569	5.07564	-5.51586	0	0
LIP5	Triacylglycerol lipase V precursor	jgi|Fusox2|3882|FOXG_09027T0:8-1770	-4.96681	0	0	0
Wall stability						
DGG	1,4-alpha-D-glucan glucohydrolase	jgi|Fusox2|10676|FOXG_13566T0:41-2337	-3.27065	0	0	0
BGL	Beta-glucanase	jgi|Fusox2|19455|FOXG_16869T0:62-845	3.46867	0	0	0
BGL1b	Beta-glucosidase 1B	jgi|Necha2|37665|e_gw1.3.378.1:52-1391	0	0	-2.59421	0
EGI	Endo-1,3(4)-beta-glucanase	jgi|Fusox2|2797|FOXG_08301T0:4-2632	2.34544	0	0	0
EGID	Endo-beta-1,4-glucanase D	jgi|Fusox2|4848|FOXG_09647T0:248-1628	5.04242	0	0	0
EGID	Endo-beta-1,4-glucanase D	jgi|Fusox2|7895|FOXG_11698T0:1-1073	5.40299	0	0	0
EGID	Endo-beta-1,4-glucanase D	jgi|Fusox2|2629|FOXG_08211T0:3-1532	2.44869	0	0	0
EGID	Endo-beta-1,4-glucanase D	jgi|Necha2|105936|fgeneshN1_pm.sca_26_chr2_2_0000010:701-1035	0	0	-3.3245	0
EGI4	Endoglucanase-4	jgi|Fusox2|20139|FOXG_04120T0:4-2343	4.27851	0	0	0
EBGA1	Glucan endo-1,3-beta-glucosidase A1	jgi|Fusox2|6323|FOXG_10637T0:7-1489	3.05712	0	0	0
EGIB	Glucan endo-1,6-beta-glucosidase B	jgi|Fusox2|5355|FOXG_10041T0:13-1435	3.18842	0	0	0
BGBp	Periplasmic B-glucosidase B-xylosidase precursor	jgi|Fusox2|23700|FOXG_05772T0:595-1099	-3.20833	0	0	0
Oxidation - Reduction and stress response						
CYP53	Benzoate 4-monooxygenase	jgi|Fusox2|5051|FOXG_09802T0:144-1025	0	6.54621	0	0
CYP53	Benzoate 4-monooxygenase	jgi|Necha2|87276|fgenesh1_pg.sca_41_chr12_2_0000109:234-410	0	0	0	5.16206
SED1	Cell wall sed1	jgi|Necha2|99160|estExt_fgenesh1_pg.C_sca_18_chr2_1_00441:255-468	0	0	2.28821	4.96806
GAR	D/L-glyceraldehyde reductase	jgi|Necha2|100823|estExt_fgenesh1_pg.C_sca_20_chr6_4_00077:0-1049	0	0	0	3.42304
GLD	D-galactonate dehydratase	jgi|Fusox2|7924|FOXG_11724T0:3-1211	-2.32323	0	0	0
EPHXp	Putative epoxide hydrolase	jgi|Fusox2|8047|FOXG_11813T0:0-1262	-5.29403	0	0	0
HpaM	FAD-dependent monooxygenase sdnN	jgi|Fusox2|19392|FOXG_16822T0:28-1544	0	-5.23737	0	0
G5D	Gluconate 5-Dehydrogenase	jgi|Fusox2|16044|FOXG_15642T0:0-1153	-3.84802	0	0	0
G5D	Gluconate 5-Dehydrogenase	jgi|Necha2|67785|estExt_Genewise1Plus.C_sca_54_chr3_4_00066:117-250	0	0	0	5.16206
GST	Glutathione S-transferase	jgi|Necha2|105195|estExt_fgenesh1_pm.C_sca_27_chr9_1_00003:49-831	0	0	-5.61346	0
GST	Glutathione S-transferase	jgi|Fusox2|20845|FOXG_04571T0:222-330	5.71096	0	0	0
GSTp	Glutathione S-transferase probable	jgi|Fusox2|20845|FOXG_04571T0:394-729	3.60812	0	0	0
GPD	Glycerol-3-phosphate dehydrogenase	jgi|Fusox2|5305|FOXG_10002T0:17-3122	-3.38508	0	0	0
HSP16	Heat shock protein 16	jgi|Necha2|74848|fgenesh1_pg.sca_1_chr1_3_0000459:0-980	0	0	-2.59147	-4.78838
LDH	L-lactate dehydrogenase	jgi|Fusox2|7884|FOXG_11687T0:50-1457	-3.68038	0	0	0
MDH	Malate dehydrogenase	jgi|Fusox2|25736|FOXG_07483T0:114-1263	3.36454	0	0	0
NAD-DAD	NAD-dependent aldehyde dehydrogenase	jgi|Fusox2|16587|FOXG_02858T0:89-1682	3.39606	0	0	0
NADH-D	NADH dehydrogenase	jgi|Fusox2|3565|FOXG_08787T0:4-1486	-4.06892	0	0	0
NOX	NADPH oxidase	jgi|Fusox2|2064|FOXG_01070T0:3-2711	2.59252	0	0	0
OXR	Oxidoreductase	jgi|Necha2|90089|fgenesh1_pm.sca_1_chr1_3_0000222:391-730	0	0	0	4.14059
ACOX1	Peroxisomal acyl-coenzyme A oxidase 1	jgi|Fusox2|19824|FOXG_03887T0:30-2306	-2.56662	0	0	0
PALI	pH-response regulator protein palI	jgi|Fusox2|22548|FOXG_05258T0:0-723	-3.54573	0	0	0
SFA1	S-(hydroxymethyl)glutathione dehydrogenase	jgi|Necha2|43302|e_gw1.20.192.1:1026-1175	0	0	0	5.16206
SDR	Short-chain dehydrogenase TIC 32	jgi|Fusox2|6968|FOXG_11118T1:72-1290	0	-3.49168	0	0
SDR	Short-chain dehydrogenase	jgi|Fusox2|20783|FOXG_04516T0:18-1157	4.01616	0	0	0
SDR	Short-chain dehydrogenase	jgi|Necha2|25451|gw1.20.1344.1:243-443	0	0	0	-6.22869
SUOX	Sulfite oxidase	jgi|Necha2|66028|estExt_Genewise1Plus.C_sca_18_chr2_1_00150:10-119	0	0	3.26775	0
TRX	Thioredoxin 1	jgi|Fusox2|6403|FOXG_10698T0:35-1907	-3.6411	0	0	0
TRR	Thioredoxin reductase gliT	jgi|Fusox2|12626|FOXG_02467T0:325-1292	-3.44956	0	0	0
TRXC	Thioredoxin-like protein Clot	jgi|Fusox2|16480|FOXG_02779T0:0-320	-4.28927	0	0	0
Tri4	Trichodiene oxygenase	jgi|Fusox2|14217|FOXG_14591T0:1127-1504	-6.38819	0	0	0

Gene Name	GoTerm Blast2Go	Location	*F.oxysporum* FMR 9788		*N. solani* FMR 4291	
			AMB	PSC	AMB	PSC
Transport and efflux pumps						
AQP1	Aquaporin	jgi|Necha2|90431|fgenesh1_pm.sca_1_chr1_3_0000564:23-641	0	0	0	3.81411
ABC.atrF	ABC multidrug transporter atrF	jgi|Fusox2|17789|FOXG_03541T0:66-2397	0	-3.4518	0	0
MDR1	ABC multidrug transporter mdr1	jgi|Necha2|96330|fgenesh1_pm.sca_6_chr9_2_0000288:2631-3813	0	0	-3.88845	0
ABC.C	ABC multidrug transporter C	jgi|Fusox2|16214|FOXG_15760T0:1-1797	-2.26369	0	0	0
ABC.B	ABC multidrug transporter B	jgi|Fusox2|20171|FOXG_18750T0:88-1794	-4.05211	0	0	0
ABC.fe3	ABC-type fe3+ transport system	jgi|Fusox2|16404|FOXG_02711T0:49-1251	-3.36618	0	0	0
CDR4	ABC transporter CDR4	jgi|Necha2|63187|estExt_Genewise1.C_sca_11_chr8_3_00512:4-277	0	0	0	-6.22869
CDR4	ABC transporter CDR5	jgi|Necha2|63187|estExt_Genewise1.C_sca_11_chr8_3_00512:1644-1894	0	0	0	-6.22869
AFLT	Aflatoxin efflux pump AFLT	jgi|Fusox2|4751|FOXG_09572T0:0-1161	2.8714	0	0	0
himE	Efflux pump himE	jgi|Fusox2|22796|FOXG_05386T0:14-3348	3.14279	0	0	0
himE	Efflux pump himE	jgi|Fusox2|24077|FOXG_06034T0:4-1340	-2.55841	0	0	0
himE	Efflux pump himE	jgi|Fusox2|12341|FOXG_02285T0:2-1567	0	-4.35307	0	0
RDC3	Efflux pump rdc3	jgi|Necha2|48247|e_gw1.27.37.1:630-848	0	0	-2.71246	0
MirAr	Major facilitator MirA related	jgi|Fusox2|16469|FOXG_02767T0:1207-1835	5.71096	0	0	0
MFSB	Major facilitator superfamily multidrug transporter mfsB	jgi|Fusox2|3249|FOXG_08562T0:0-2855	-4.76834	0	0	0
MDRA	Major facilitator superfamily multidrug transporter mdrA	jgi|Fusox2|5001|FOXG_09760T0:8-2201	-4.2846	0	0	0
ataA	MFS acetylaranotin efflux transporter ataA	jgi|Fusox2|4751|FOXG_09572T0:0-1161	0	-4.4166	0	0
MDRr	MFS multidrug-resistance transporter related	jgi|Fusox2|22637|FOXG_05310T0:214-585	5.71096	0	0	0
asaE	MFS transporter asaE	jgi|Fusox2|25642|FOXG_07399T0:78-1457	0	-4.94552	0	0
asaE	MFS transporter asaE	jgi|Fusox2|21965|FOXG_04915T0:272-1685	2.80736	0	0	0
prl	MFS transporter prl	jgi|Fusox2|7440|FOXG_11412T0:0-2787	-2.94551	0	0	0
Umfs	Uncharacterized MFS-type transporter	jgi|Fusox2|15508|FOXG_15460T0:15-2472	-2.79417	0	0	0
MDR3	Multidrug resistance protein 3	jgi|Necha2|43313|e_gw1.20.1298.1:75-380	0	0	0	-6.22869
MDR	Multidrug transporter	jgi|Necha2|49776|e_gw1.43.147.1:925-1447	0	0	0	-6.22869
MDRr	Multidrug resistant protein related	jgi|Fusox2|16153|FOXG_15713T0:1102-1512	0	6.54621	0	0
MirB	Siderophore iron transporter mirB	jgi|Fusox2|10432|FOXG_13371T0:46-2332	2.39967	0	0	0
estB	Siderophore triacetylfusarinine C esterase	jgi|Fusox2|5027|FOXG_09781T0:33-1122	3.2784	0	0	0
SFC1	Succinate/fumarate mitochondrial transporter	jgi|Fusox2|12532|FOXG_02391T0:0-138	0	6.54621	0	0
SFC1	Succinate/fumarate mitochondrial transporter	jgi|Necha2|79543|fgenesh1_pg.sca_19_chr4_3_0000051:0-969	0	0	-3.70686	0
SFC1	Succinate/fumarate mitochondrial transporter	jgi|Fusox2|12530|FOXG_02390T1:0-632	-2.84397	0	0	0
